# A Bayesian Semiparametric Regression Model for Joint Analysis of Microbiome Data

**DOI:** 10.3389/fmicb.2018.00522

**Published:** 2018-03-26

**Authors:** Juhee Lee, Marilou Sison-Mangus

**Affiliations:** ^1^Department of Applied Mathematics and Statistics, University of California, Santa Cruz, Santa Cruz, CA, United States; ^2^Department of Ocean Sciences, University of California, Santa Cruz, Santa Cruz, CA, United States

**Keywords:** count data, Laplace prior, metagenomics, microbiome, regularizing prior, process convolution, negative binomial model, 16S ribosomal RNA sequencing

## Abstract

The successional dynamics of microbial communities are influenced by the synergistic interactions of physical and biological factors. In our motivating data, ocean microbiome samples were collected from the Santa Cruz Municipal Wharf, Monterey Bay at multiple time points and then 16S ribosomal RNA (rRNA) sequenced. We develop a Bayesian semiparametric regression model to investigate how microbial abundance and succession change with covarying physical and biological factors including algal bloom and domoic acid concentration level using 16S rRNA sequencing data. A generalized linear regression model is built using the Laplace prior, a sparse inducing prior, to improve estimation of covariate effects on mean abundances of microbial species represented by operational taxonomic units (OTUs). A nonparametric prior model is used to facilitate borrowing strength across OTUs, across samples and across time points. It flexibly estimates baseline mean abundances of OTUs and provides the basis for improved quantification of covariate effects. The proposed method does not require prior normalization of OTU counts to adjust differences in sample total counts. Instead, the normalization and estimation of covariate effects on OTU abundance are simultaneously carried out for joint analysis of all OTUs. Using simulation studies and a real data analysis, we demonstrate improved inference compared to an existing method.

## 1. Introduction

Microbial communities are influenced by several factors whether they live in the host's guts or other occupied niches. Their successional dynamics could further change in response to perturbations of the host or of the surrounding environments (Turnbaugh et al., 2009; Needham and Fuhrman, 2016). Understanding how abiotic and biotic factors influence the dynamics of microbial communities is of great interest in the field of microbiome studies. Recent revolutionary advances in next-generation sequencing (NGS) technologies along with rapidly decreasing costs, have facilitated the accumulation of large datasets of 16S ribosomal RNA (rRNA) amplicon sequences across various disciplines such as medicine, biology, ecology, and environmental sciences (Woo et al., 2008). Sequencing data is usually pre-treated for quality filtering, noise removal and chimera checking through bioinformatics algorithms and the filtered sequences are clustered into Operational Taxonomic Units (OTUs), which represent similar organisms (microbial species) based on sequence homology (called OTU picking). An OTU abundance table is generated, recording counts for OTUs in samples. Further statistical data analyses are then performed using the OTU table to answer biological and ecological questions.

**Figure 1:**
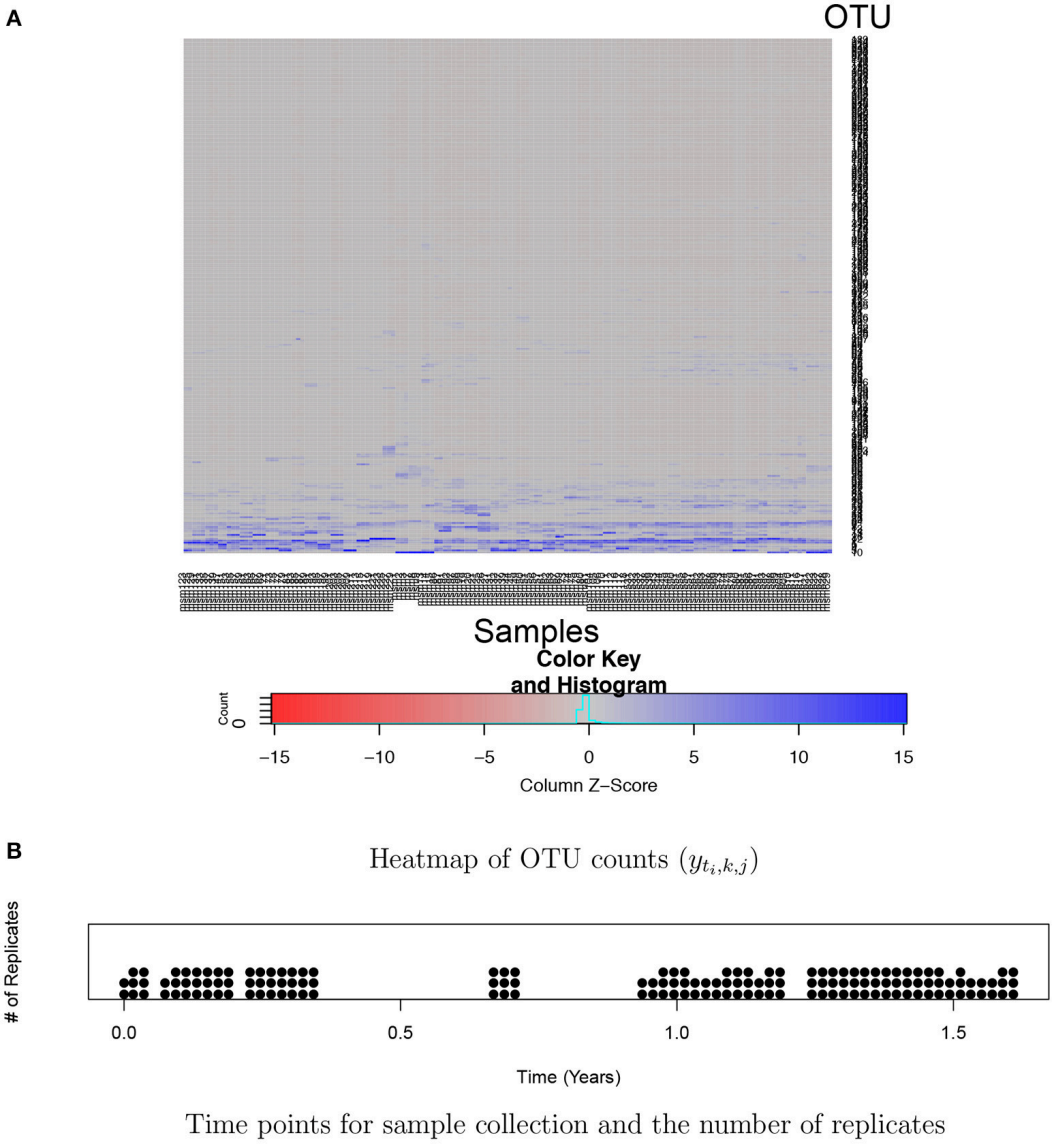
Ocean microbiome data. **(A)** Heatmap of OTU counts (*y*_*t*_*i*_,*k, j*_). OTU and samples are in rows and columns, respectively. OTU counts are rescaled within a sample for better illustration. **(B)** 55 time points where ocean microbiome samples were collected are marked on the X-axis and the number of dots at a time point represents the number of replicates (*K*_*i*_) at the time point.

Analysis of huge NGS data is computationally expensive and challenging. One of the key challenges is the normalization of counts across samples. Total counts (often called library size or sequencing depth) may vastly vary across different samples due to technical reasons. Thus, observed counts are not directly comparable across samples and cannot be used as a measure of the abundance of an OTU. Normalized counts through rarefaction or relative frequencies are commonly used for easy comparison of OTU abundance across samples. However, such *ad hoc* normalization procedures have been criticized from a statistical perspective since using pre-normalized quantities may undermine the performance of downstream analysis (McMurdie and Holmes, 2014). Another challenge is high dimensionality and sparsity in OTU count data. A dataset typically includes hundreds or thousands of OTUs and a majority of them has zero or very low frequencies in most of samples. For example, Figure 1A illustrates a heatmap of OTU counts in our motivating dataset described in section 2.3. It shows that a majority of OTUs has very low counts (gray) in a sample, and the set of OTUs having large counts (blue) vary across samples. Due to such sparsity in data, borrowing strength across OTUs through joint analysis of all OTUs is crucial for improved inference. Recently, various statistical methods including Romero et al. (2014), Chen and Li (2016), Gibbons et al. (2017), and Zhang et al. (2017) have been developed for microbiome studies using NGS data. For example, Zhang et al. (2017) used a negative binomial mixed regression model to study interactions between the microbiome and host environmental/clinical factors. Random effects are used to induce correlation among samples from a group. Common to most of recent methods including Zhang et al. (2017) is separately analyzing each OTU at a time.

We develop a Bayesian semiparametric generalized linear regression model to study the effects of physical and biological factors on abundance of microbes. The proposed method performs mode-based normalization through a hierarchical model, which enables direct modeling of OTU counts. Furthermore, the hierarchical model facilitates borrowing strength between OTUs, between samples, and between time points through joint analysis and improves inference on the effects of covariates **X** on OTU abundance which are the parameters of our primary interest. Specifically, a negative binomial (NB) distribution parameterized by a mean parameter μ and an overdispersion parameter *s* is assumed for OTU counts. The NB distribution flexibly accommodates overdispersion often seen in NGS data and is commonly used as a robust alternative to a Poisson distribution (Anders and Huber, 2010). The expected count μ of an OTU is decomposed as a product of factors, a baseline mean count *g* and a nonnegative function η(**X**) of covariates that describes their effects on the mean count. We use the log link function for η(**X**) and assume that change in a covariate has a multiplicative effect on mean count, where the associated coefficient quantifies the size and direction of the effect. We consider a Laplace prior for the coefficients, a shrinkage prior that is essential in a high dimensional regression setting. Shrinkage priors in regression yield sparse point estimates of the coefficients, where many of the coefficients have values close to zero and few have large values. The sparse estimates improve out-of-sample prediction and produce more interpretable models (Park and Casella, 2008). In addition, shrinkage priors such as a Laplace prior in a regression problem mitigate potential problems by multicollinearity and yield improved coefficient estimates when covariates are high-dimensional and potentially highly correlated (Polson and Scott, 2012). For baseline mean counts, we develop a nonparametric model to combine all OTUs for joint analysis. Baseline mean counts may vary across samples and OTUs. Also, as in our motivating data for which samples were taken over time, there may be temporal dependence in baseline mean counts. To tackle the problem, we further decompose the baseline count *g* into sample size factor (*r*), OTU size factor (α_0_), and OTU and time factor (α_*t*_), that is, *g* = *r* × α_0_ × α_*t*_. Due to the overparametrization of the baseline mean abundance, individual factors are not identifiable. To avoid identifiability issues, we place the regularizing priors with mean constraints (Li et al., 2017) for sample size factor *r* and OTU size factor α_0_. In addition, we model a temporal dependence structure between the baseline expected counts for an OTU through a convolutional Gaussian process (Higdon, 1998). The process convolution approach is often used as an alternative approach of the Gaussian process to construct a dependent process due to its efficient computation (Lee et al., 2005; Liang and Lee, 2014). Through simulation studies, we show that estimates of individual parameters *r*, α_0_, and α_*t*_ are not fully interpretable under the proposed model, but baseline mean counts *g* are identifiable. The model also provides a posterior distribution of *g* for uncertainty quantification.

The rest of the paper is organized as follows. In section 2 we describe the proposed model and discuss the prior formulations and the resulting posterior inference. We perform simulation studies to assess the proposed model and perform comparison with an existing method that analyzes one OTU at a time. We then apply the proposed model to an ocean microbiome dataset. Section 3 presents the performance of the proposed model from the simulation experiment and the ocean microbime data. Section 4 concludes the paper with a discussion on limitations and possible extensions.

## 2. Materials and methods

### 2.1. Bayesian semiparametric regression model

Suppose that samples are taken at *n* different time points, 0 ≤ *t*_*i*_ ≤ *T*, *i* = 1, …, *n*, and with *K*_*i*_ replicates at time point *t*_*i*_. We consider count *y*_*t*_*i*_,*k, j*_ of OTU *j* in replicate *k* taken at time *t*_*i*_, where *i* = 1, …, *n*, *k* = 1, …, *K*_*i*_, and *j* = 1, …, *J*. A sample is thus indexed by *t*_*i*_ and *k*. We let the total number of samples $N=\overset{n}{\underset{i=1}{\sum }}K_{i}$. Let **Y** = [*y*_*t*_*i*_,*k, j*_] denote the *N*×*J* matrix of counts, where *y*_*t*_*i*_,*k, j*_ is integer-valued and nonnegative. Also, suppose that covariates ${\text{X}}_{t_{i}}={\left(X_{t_{i},1},\ldots ,X_{t_{i},P}\right)}^{\prime }$ are recorded at time *t*_*i*_. For example, covariates are physical and biological factors in our motivating data.

#### 2.1.1. Sampling model

Count data by NGS methods is often modeled through a Poisson distribution. The assumption under the Poisson distribution that the variance is equal to the mean is often too restrictive to accommodate overdispersion that variation in data exceeds the mean. The negative binomial (NB) distribution is a popular and convenient alternative to address the overdispersion problem and is widely recognized as a model that provides improved inference to NGS count data (for example, see Robinson and Smyth, 2007; Anders and Huber, 2010). A NB distribution can be characterized by mean and overdispersion parameters. We suppress index *i* for simpler notation and assume a NB model for count *y*_*t,k,j*_ of OTU *j* in replicate *k* at time *t*,

(1) $$y_{t,k,j}\overset{indep}{\sim }\text{NB}(\mu_{t,k,j},\;s_{j}),$$

where mean count μ_*t,k,j*_ > 0 and overdispersion parameter *s*_*j*_ > 0. The model in Equation (1) implies that count of OTU *j* in replicate *k* at time *t* has mean E(*y*_*t,k,j*_ ∣ μ_*t,k,j*_) = μ_*t,k,j*_ and variance $\text{Var}\left(y_{t,k,j}|\mu_{t,k,j},s_{j}\right)=\mu_{t,k,j}+\mu_{t,k,j}^{2}s_{j}$. The model allows different dispersion levels across OTUs through OTU-specific overdispersion parameters *s*_*j*_. In the limit as *s*_*j*_ → 0, the model in Equation (1) yields the Poisson distribution with mean μ_*t,k,j*_. We assume a gamma distribution for a prior distribution of *s*_*j*_, $s_{j}\overset{iid}{\sim }\text{Ga}\left(a_{s},b_{s}\right)$, *j* = 1, …, *J*, with fixed *a*_*s*_ and *b*_*s*_.

#### 2.1.2. Model for regression

We next model the mean count μ_*t,k,j*_ of *y*_*t,k,j*_. We decompose the mean count into factors, a baseline mean count and a function of covariates, μ_*t,k,j*_ = *g*_*t,k,j*_η_*j*_(**X**_*t*_). Here parameter *g*_*t,k,j*_ denotes the baseline mean abundance of OTU *j* in sample (*t, k*) and η_*j*_(**X**_*t*_) is a function of covariates **X**_*t*_ for OTU *j* to model the covariate effects. We construct a generalized regression model by letting $\log\left(\eta_{j}\left({\text{X}}_{t}\right)\right)={\text{X}}_{t}^{\prime }\beta_{j}$, where $\beta_{j}={\left(\beta_{j1},\ldots ,\beta_{jP}\right)}^{\prime }$ is a *P*-dimensional vector of regression coefficients of OTU *j* (Lawless, 1987; McCullagh and Nelder, 1989). The coefficient β_*j, p*_ quantifies the effect of covariate *p* *X*_*p*_ on the mean abundance of OTU *j*. A vector β_*j*_ close to the zero vector produces a value of η_*j*_(**X**_*t*_) close to 1, and the mean count remains similar to the baseline mean count *g*_*t,k,j*_, implying insignificant covariate effects. A negative (positive) of β_*j, p*_ implies a negative (positive) association between mean counts and the *p*-th covariate, and a larger value of *X*_*j,p*_ decreases (increases) the mean count, while holding the other covariates constant. We consider a Laplace prior on β_*j*_. Specifically, we express the Laplace distribution as a scale mixture of normals and assume for *j* = 1, …, *J* and *p* = 1, …, *P*,

(2) $$\begin{array}{l} \beta_{j,p}|\sigma_{j}^{2},\phi_{j,p}\overset{indep}{\sim }\text{N}(0,\sigma_{j}^{2}\phi_{j,p}),\;\phi_{j,p}\overset{indep}{\sim }\text{Exp}(\frac{\lambda_{j}^{2}}{2}),\\ \;\lambda_{j}^{2}\overset{iid}{\sim }\text{Ga}(a_{\lambda },b_{\lambda }),\;\sigma_{j}^{2}\overset{iid}{\sim }\text{IG}(a_{\sigma },b_{\sigma }), \end{array}$$

where *a*_λ_, *b*_λ_, *a*_σ_, and *b*_σ_ are fixed. $\sigma_{j}^{2}$ and ϕ_*j,p*_ denote the global and local shrinkage parameters, respectively, for OTU *j*. After integrating ϕ_*j,p*_ out, the prior distribution of β_*j,p*_ is the Laplace distribution with location parameter 0 and scale parameter $\sqrt{\sigma_{j}^{2}}/\lambda_{j}$, that is, $p\left(\beta_{j,p}|\lambda_{j}^{2},\sigma_{j}^{2}\right)\propto$ $\exp\left(-\lambda_{j}|\beta_{j,p}|/\sqrt{\sigma_{j}^{2}}\right)$. Compared to a normal distribution that is a common choice for the prior of β_*j,p*_, the Laplace distribution has more concentration around zero but allows heavier tails. The regularized regression through the Laplace prior more shrinks the coefficients of insignificantly related covariates into zero and less pulls the coefficients of important covariates toward zero. Shrinkage of β estimates through the model in Equation (2) mitigates possible issues due to multicollinearity and efficiently improves estimation of β in a high dimensional setting (Polson and Scott, 2012).

#### 2.1.3. Model for baseline mean count

We next build a prior probability model for the baseline mean count *g*_*t,k,j*_ of OTU *j* in sample (*t, k*). We assume *g*_*t,k,j*_ = *r*_*t,k*_α_0, *j*_α_*t, j*_ to separate sample (*r*_*t,k*_), OTU (α_0, *j*_), and OTU-time (α_*t, j*_) factors. Sample total counts $y_{t,k,\cdot }=\overset{J}{\underset{j=1}{\sum }}y_{t,k,j}$ may greatly differ for different samples possibly due to experimental artifacts. For example, counts of an OTU even in the replicates taken at a time point may vastly differ. Sample specific size factors *r*_*t,k*_ account for different total counts in different samples and expected counts normalized by *r*_*t,k*_ are comparable across samples. Factor α_0, *j*_ explains variabilities in baseline mean abundances of OTUs and α_*t,j*_ models temporal dependence of the mean counts for an OTU, respectively. Factors α_0, *j*_ and α_*t,j*_ are not indexed by replicate *k* and account for stochastic change over time in normalized baseline expected counts of OTU *j*. Collecting all, we write the mean count as

(3) $$\mu_{t,k,j}=g_{t,k,j}\eta_{j}(X_{t})=r_{t,k}\alpha_{0,j}\alpha_{t,j}\eta_{j}(X_{t}),$$

The model for *g*_*t,k,j*_ in Equation (3) is overparameterized and the individual parameters are not identifiable. To avoid potential identifiability issues, many of NB models rely on some form of approximation for the baseline mean counts. For example, one may find the maximum likelihood estimates (MLEs) of baseline mean abundance under some constraints and plug in those estimates to infer the mean abundance levels μ_*t*_*i*_, *j*_ of OTUs (Witten, 2011). Plugging in MLEs is simple but may not be robust. In particular, the inference is greatly affected by a small change in a few OTUs that have large counts. Moreover, the errors introduced in the baseline mean count estimation will not be reflected in the inference. Several approaches to robustify the estimates are proposed (for example, see Anders and Huber, 2010; Witten, 2011). To circumvent the identifiability issue and provide uncertainty quantification for estimation of *g*_*t,k,j*_, we take an alternative in Li et al. (2017) by imposing regularizing priors with mean constraints for *r*_*t,k*_ and α_0, *j*_. We let the logarithm of the factors ${\tilde{r}}_{t,k}=\log\left(r_{t,k}\right)$ and ${\tilde{\alpha }}_{0,j}=\log\left(\alpha_{0,j}\right)$, and assume the regularizing prior distribution with mean constraints,

(4) $$\begin{array}{l} {\tilde{r}}_{t_{i},k}|\psi^{r},\eta^{r},w^{r},v_{r}^{2},c_{r}\overset{iid}{\sim }\;\sum_{\ell =1}^{L^{r}}\psi_{\ell }^{r}\left\{w_{\ell }^{r}\phi (\eta_{\ell }^{r},v_{r}^{2})+(1-w_{\ell }^{r})\phi \left(\frac{c_{r}-w_{\ell }^{r}\eta_{\ell }^{r}}{1-w_{\ell }^{r}},v_{r}^{2}\right)\right\},\\ {\tilde{\alpha }}_{0,j}|\psi^{\alpha },\eta^{\alpha },w^{\alpha },v_{\alpha }^{2},c_{\alpha }\;\overset{iid}{\sim }\;\sum_{\ell =1}^{L^{\alpha }}\psi_{\ell }^{\alpha }\left\{w_{\ell }^{\alpha }\phi (\eta_{\ell }^{\alpha },v_{\alpha }^{2})+(1-w_{\ell }^{\alpha })\phi \left(\frac{c_{\alpha }-w_{\ell }^{\alpha }\eta_{\ell }^{\alpha }}{1-w_{\ell }^{\alpha }},v_{\alpha }^{2}\right)\right\}, \end{array}$$

where ϕ(η, *v*^2^) is the probability density function of the normal distribution with mean η and variance *v*^2^, constraints for the mixture weights $\overset{L^{r}}{\underset{\ell =1}{\sum }}\psi_{\ell }^{r}=\overset{L^{\alpha }}{\underset{\ell =1}{\sum }}\psi_{\ell }^{\alpha }=1$ with $0<\psi_{\ell }^{r}<1$ and $0<\psi_{\ell }^{\alpha }<1$, $0<w_{\ell }^{r}<1$, and $0<w_{\ell }^{\alpha }<1$ for all ℓ. Mixture models as in Equation (4) are often used as a basis to approximate any distribution. Each component in Equation (4) is further composed of a mixture of two normals, $\text{N}\left(\eta_{\ell },v^{2}\right)$ and $\text{N}\left(\frac{\left(c-w_{\ell }\eta_{\ell }\right)}{\left(1-w_{\ell }\right)},v^{2}\right)$ with weights *w*_ℓ_ and 1 − *w*_ℓ_, respectively, and the mean of the component is *c*. In consequence, the prior and posterior of $\tilde{r}$ and $\tilde{\alpha }$ under the model in Equation (4) satisfy their prespecified mean constraints *c*_*r*_ and *c*_α_, respectively. Li et al. (2017) showed that the model in Equation (4) flexibly accommodates various features in a distribution such as skeweness or multi-modality while satisfying the constraints. Furthermore, the model based normalization through Equation (4) enables joint analysis of all OTUs and can further improve estimation of the covariate effects. With the regularizing priors, baseline mean counts *g*_*t,k,j*_ are identifiable, while *r*_*t,k*_, α_0, *j*_, and α_*t,j*_ are not directly interpretable. More importantly, the parameters of primary interest η_*j*_(**X**_*t*_) can be uniquely estimated and β_*j,p*_'s keep their interpretation as parameters that quantify the effects of covariates on mean counts. We used an empirical approach to fix the mean constraints *c*_*r*_ and *c*_α_. Sensitivity analyses were conducted to assess the robustness to the specification of *c*_*r*_ and *c*_α_ and show that the model provides reasonable estimates of *g*_*t,k,j*_ and moderate changes in the values of *c*_*r*_ and *c*_α_ minimally change the estimates. More details of the specification of *c*_*r*_ and *c*_α_ are discussed in section 3.1. We fix the numbers of mixture components, *L*^*r*^ and *L*^α^ and variances $v_{r}^{2}$ and $v_{\alpha }^{2}$. We let $\eta_{\ell }^{r}\overset{iid}{\sim }\text{N}\left(c_{r},\omega_{r}^{2}\right)$ and $\eta_{\ell }^{\alpha }\overset{iid}{\sim }\text{N}\left(c_{\alpha },\omega_{\alpha }^{2}\right)$, where $\omega_{r}^{2}$ and $\omega_{\alpha }^{2}$ are fixed. We assume $\psi^{r}=\left(\psi_{1}^{r},\ldots ,\psi_{L^{r}}^{r}\right)\sim \text{Dir}\left(d_{r},\ldots ,d_{r}\right)$ and $\psi^{\alpha }=\left(\psi_{1}^{\alpha },\ldots ,\psi_{L^{\alpha }}^{\alpha }\right)\sim \text{Dir}\left(d_{\alpha },\ldots ,d_{\alpha }\right)$, with fixed *d*_*r*_ and *d*_α_. We let $w_{\ell }^{r}\overset{iid}{\sim }\text{Be}\left(a_{r},b_{r}\right)$, ℓ = 1, …, *L*^*r*^ and $w_{\ell }^{\alpha }\overset{iid}{\sim }\text{Be}\left(a_{\alpha },b_{\alpha }\right)$, ℓ = 1, …, *L*^α^ with fixed *a*_*r*_, *b*_*r*_, *a*_α_, and *b*_α_.

Recall that samples are collected over time points *t*_1_, …, *t*_*n*_ in [0, *T*] and α_*t,j*_ accounts for temporal dependence in the baseline mean count for an OTU. We let ${\tilde{\alpha }}_{t,j}=\log\left(\alpha_{t,j}\right)$ a function in time *t* and use a stochastic process to model temporal dependence among μ_*t,k,j*_. The Gaussian process (GP) is one of the most popular stochastic models for the underlying process in spatial and spatio-temporal data (for example, see Cressie, 1992; Banerjee et al., 2014 among many others). The GP effectively represents the underlying phenomenon in a variety of applications, but it has some drawbacks such as a complex computation that requires a matrix decomposition and problematic estimation of the parameters in its covariance function, potentially leading to difficulties in exploring the posterior distribution (Lee et al., 2005; Liang and Lee, 2014). To alleviate such difficulties of GP models while still maintaining their flexibility and adaptiveness, we use a convolution approach with a kernel function developed in Higdon (1998, 2002). For each OTU, we specify the latent process **θ**_*j*_(*t*) to be nonzero only at the time points *u*_1_, …, *u*_*M*_ in [0, *T*]. Specifically, we consider the GP convolution model,

$${\tilde{\alpha }}_{t,j}=\sum_{m=1}^{M}Z(t-u_{m})\theta_{m,j},$$

where {*u*_1_, …, *u*_*M*_} a set of basis points in $\left[-t_{1}^{\prime },T+t_{2}^{\prime }\right]$ with $t_{1}^{\prime },t_{2}^{\prime }>0$, and *Z*(*t* − *u*_*m*_) a Gaussian kernel centered at *u*_*m*_, $Z\left(t-u_{m}\right)=\frac{1}{\sqrt{2\pi \gamma^{2}}}\exp\left\{\frac{-{\left(t-u_{m}\right)}^{2}}{2\gamma^{2}}\right\}$. The number of basis points *M*, their locations *u*_*m*_ and the range parameter γ can be treated as random variables by placing prior distributions, e.g., consider a gamma prior for γ. For simplicity, we fix them as follows. We first choose a value for *M* and let *u*_*m*_ evenly spaced over time $\left[-t_{1}^{\prime },T+t_{2}^{\prime }\right]$. Following Xiao (2015), we let the range parameter $\gamma^{2}={\left(\left(2T+t_{1}^{\prime }+t_{2}^{\prime }\right)/M\right)}^{2}$, that is, the range parameter depends on the value of *M*. Through simulations, we studied the impact of different values of *M* on the posterior inference of *g*_*t,k,j*_. A discussion is included in section 3.1. Given the number of basis points *M*, we assume $\theta_{m,j}|\tau_{j}^{2}\overset{indep}{\sim }\text{N}\left(0,\tau_{j}^{2}\right)$ and $\tau_{j}^{2}\overset{iid}{\sim }\text{IG}\left(a_{\tau },b_{\tau }\right)$, *m* = 1, …, *M* and *j* = 1, …, *J*.

We implement posterior inference on the parameters $\tilde{\theta }=(\beta_{j},\sigma_{j}^{2},\lambda_{j}^{2},\phi_{j,p},{\tilde{r}}_{t,k},\psi^{r},w_{\ell }^{r},\eta_{\ell }^{r},{\tilde{\alpha }}_{0,j},\;{\tilde{\alpha }}_{t,j},\psi^{\alpha },w_{\ell }^{\alpha },\eta_{\ell }^{\alpha },\theta j,\tau_{j}^{2},s_{j})$ via a Markov chain Monte Carlo (MCMC) method based on Metropolis-Hastings algorithm and Gibbs sampling. Each of the parameters is iteratively updated conditional on the currently computed values of all other parameters to simulate a sample from the posterior distribution. The parameters $\tilde{r}$ and ${\tilde{\alpha }}_{0}$ jointly determine baseline mean counts and joint updating of $\tilde{r}$ and ${\tilde{\alpha }}_{0}$ may greatly improve the mixing. In our ocean microbiome data, some discretized covariates are missing. We treat them as random variables by assuming a uniform distribution over possible categories, and impute their values in MCMC simulation. Full details of our MCMC algorithm are given in Supplementary section 1. We diagnose convergence and mixing of the described posterior MCMC simulation using trace plots and autocorrelation plots of imputed parameters. For the upcoming simulation examples and the data analysis, we found no evidence of practical convergence problems. An R package of the code used for simulations and the analysis of the ocean microbiome dataset in the following sections is available from the authors website https://users.soe.ucsc.edu/~juheelee/.

### 2.2. Simulation experiment: data generation and comparative study

**Figure 2:**
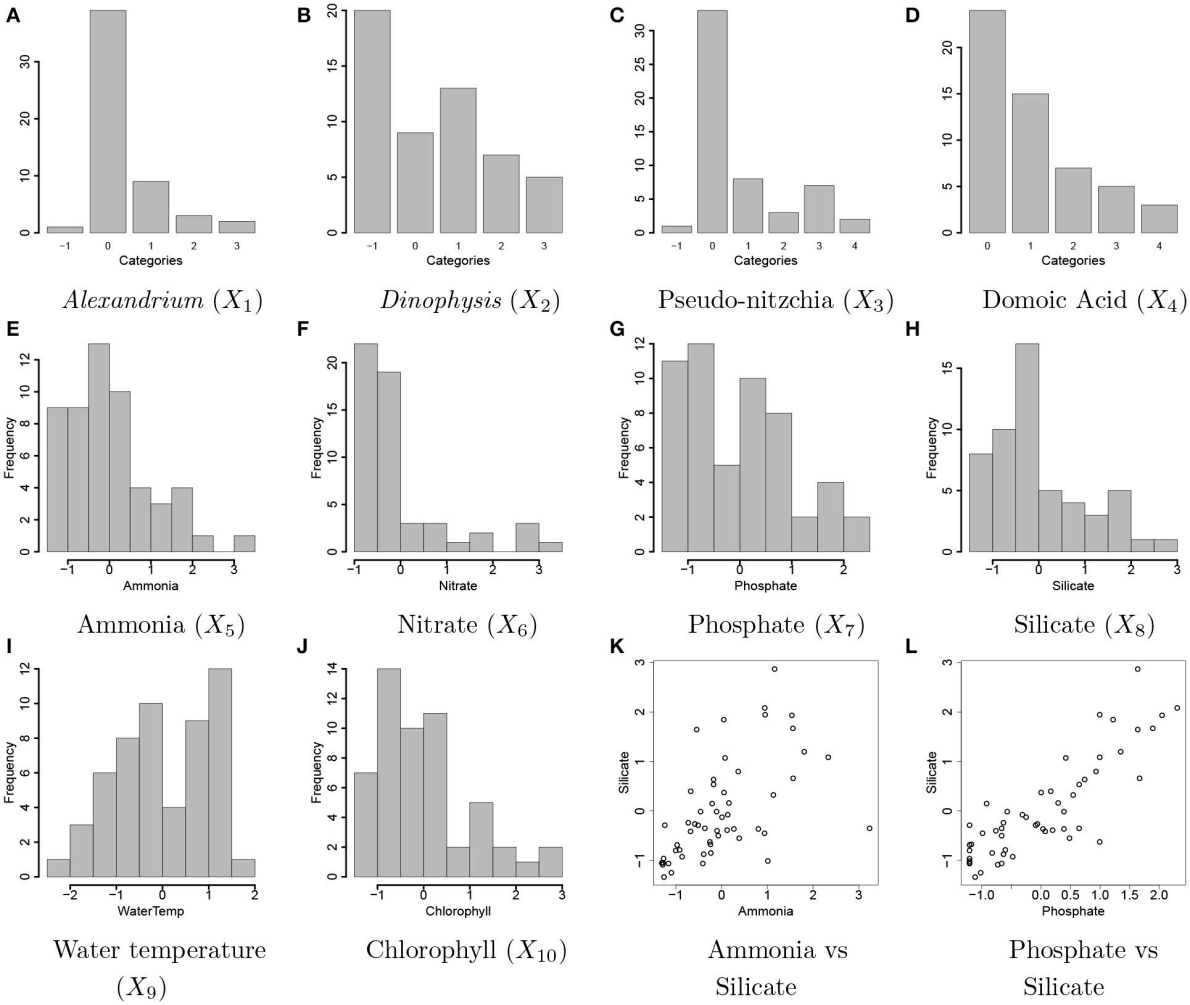
Ocean microbiome data. Bar plots of discretized covariates, concentration levels of *Alexandrium* (Ax, *X*_1_) and *Dinophysis* (Dp, *X*_2_), Pseudo-nitzchia (Pn, *X*_3_), domoic acid (DA, *X*_4_) in **(A–D)**. The values of −1, 0, 1, 2, 3, and 4 represent a missing value, none and low, medium, high, and highest concentration levels, respectively. Histograms of continuous covariates, concentration levels of ammonia (NH_4_, *X*_5_), nitrate (N, *X*_6_), phosphate (P, *X*_7_), silicate (Si, *X*_8_), water temperature (T, *X*_9_), concentration level of chlorophyll (Chl, *X*_10_) in **(E–J)**. The variables are standardized to have mean 0 and variance 1 prior to analysis. A scatterplot of the concentrations of ammonia and silicate and a scatterplot of the concentrations of phosphate and silicate are shown in **(K,L)**, respectively.

We conducted simulation studies to assess the performance of our model. We compared the model to an alternative model, the negative binomial mixed model (NBMM) in Zhang et al. (2017). We assumed a sample of *J* = 200 OTUs. We used the same time points (*t*_*i*_) and numbers of replicates (*K*_*i*_) of our ocean microbiome data as shown in Figure 1B. We let $\beta_{j,p}^{\text{TR}}=0$ with probability 0.85. For $\beta_{j,p}^{\text{TR}}\neq 0$ we simulated $\beta_{j,p}^{\text{TR}}$ from either of N(−1.5, 0.05^2^) or N(1.5, 0.05^2^) with equal probability, where N(*a, b*^2^) denotes the normal distribution with mean *a* and variance *b*^2^. It implies that a covariate has no effect on OTU abundance with probability 0.85 or may significantly affect mean abundance with the remaining probability 0.15. To specify $r_{t,k}^{\text{TR}}$ and $\alpha_{0,j}^{\text{TR}}$, we did not assume any distribution and used their classical estimates from our ocean microbiome data; following Witten (2011), we first computed estimates of sample size factors $r_{t_{i},k}^{\prime }$ and OTU size factors $\alpha_{0,j}^{\prime }$ using the ocean microbiome data, $r_{t_{i},k}^{\prime }=y_{t_{i},k,\cdot }/y_{\cdot \cdot \cdot }$ and $\alpha_{0,j}^{\prime }=\frac{1}{N}\overset{n}{\underset{i=1}{\sum }}\overset{K_{i}}{\underset{k=1}{\sum }}y_{t_{i},k,j}/r_{t_{i},k}^{\prime }$ where $y_{t_{i},k,\cdot }=\overset{J}{\underset{j=1}{\sum }}y_{t_{i},k,j}$ and $y_{\cdot \cdot \cdot }=\overset{J}{\underset{j=1}{\sum }}y_{\cdot \cdot ,j}$. We then randomly sampled from the pool of $r_{t,k}^{\prime }$ and $\alpha_{0,j}^{\prime }$ to specify the true values. To simulate temporal dependence in OTU abundance, we let ${\tilde{\alpha }}_{t_{i},j}^{\text{TR}}=a_{t_{i},j}\cos\left(2\pi \left({\tilde{t}}_{i}-b_{t_{i},j}\right)\right)+c_{t_{i},j}{\left({\tilde{t}}_{i}-{\tilde{t}}^{⋆}\right)}^{2}.$ Here ${\tilde{t}}_{i}$ denotes time *t*_*i*_ in year and ${\tilde{t}}^{⋆}$ the median of ${\tilde{t}}_{i}$. We let $a_{t,j}\overset{iid}{\sim }\text{N}\left(0.15,0.1^{2}\right)$, $b_{t,j}\overset{iid}{\sim }\text{N}\left(0,0.5^{2}\right)$, and $c_{t,j}\overset{iid}{\sim }\text{N}\left(0.1,0.1^{2}\right)$ to have different patterns for OTUs. For some OTUs, ${\tilde{\alpha }}_{t_{i},j}^{\text{TR}}$ are illustrated in red squares in **Figures 4E–G**. We generated $s_{j}^{\text{TR}}\overset{iid}{\sim }\text{Ga}\left(1,10\right)$. We used the covariate matrix of the ocean microbiome data illustrated in Figure 2 for the simulation study. For the missing covariates in the data, we generated a value of possible categories with equal probability. We finally simulated OTU counts *y*_*t*_*i*_,*k, j*_ from the negative binomial distribution $y_{t_{i},k,j}\overset{indep}{\sim }\text{NB}\left(\mu_{t_{i},k,j}^{\text{TR}},s_{j}^{\text{TR}}\right),$ where $\mu_{t_{i},k,j}^{\text{TR}}=r_{t_{i},k}^{\text{TR}}\alpha_{0,j}^{\text{TR}}\exp\left({\tilde{\alpha }}_{t_{i},j}^{\text{TR}}+{\text{X}}_{t}^{\text{TR}}\beta_{j}^{\text{TR}}\right)$.

For comparison, we used the negative binomial mixed model (NBMM) in Zhang et al. (2017). Similar to the proposed model, the NBMM uses a negative binomial distribution with mean μ^NBMM^ and shape parameter θ^NBMM^ to model OTU counts and assumes $\log(\mu_{t,k,j}^{\text{NBMM}})=\log(yt,k,\cdot )+\beta_{0,j}^{\text{NBMM}}+t\beta_{j}^{\text{NBMM}}+Z_{t,k}b_{j}^{\text{NBMM}}$ where **X**_*t*_ and ***Z***_*t,k*_ are the covariate matrices for fixed effects and random effects, respectively. It assumes random effects $b_{j}^{\text{NBMM}}\sim \text{N}\left(0,\Psi \right)$. By letting the replicates at a time point share the same random effect, OTU abundances in the replicates at a time point are correlated. The NBMM normalizes OTU counts by sample total counts. That is, sample total counts *y*_*t,k*, **·**_ are used as an offset to adjust for the variability in total counts across samples. Similar to other existing methods, the NBMM performs separate analyses of OTUs. An iterative weighted least squares algorithm is developed to produce the MLEs under the NBMM and implemented in a R function *glmm* in R package *BhGLM*.

### 2.3. Ocean microbiome data: data description and preprocessing

We applied the proposed statistical method to ocean microbiome data. Seawater samples were collected weekly at the end of Santa Cruz Municipal Wharf (SCW), Monterey Bay (36.958 ^o^N, −122.017 ^o^W), with an approximate depth of 10 m. SCW is one of the ocean observing sites in Central and Northern California (CenCOOS), where harmful algal bloom species [HAB species: *Alexandrium* (Ax), *Dinophysis* (Dp), *Pseudo-nitzschia* (Pn) etc.] are monitored weekly along with nutrient measurements [ammonia (NH_4_), silicate (Si), nitrate (N), phosphate (P)], temperature (T), domoic acid (DA) concentration, and chlorophyll (Chl). Details of phytoplankton net tow sampling of measuring phytoplankton abundance, measurement of physical (nutrients and temperature) and biological parameters (chlorophyll α and DA concentration) are described in Sison-Mangus et al. (2016). *Pseudo-nitzschia, Dinophysis*, and *Alexandrium* cells were counted with a Sedgewick rafter counter under the microscope. Data for physical and biological factors are available from the website link http://www.sccoos.org/query/?project=Harmful%20Algal%20Blooms&study[]=Santa%20Cruz%20Wharf. Among the 10 variables, the concentration levels of *Alexandrium, Dinophysis, Pseudo-nitzschia*, and domoic acid have highly right-skewed distributions and are discretized into categories based on their biological properties for our analysis. The ranges of the concentration levels for the discretization are in Supplementary Table 1 and Figures 2A–J illustrates all covariates included for analysis. The values of −1, 0, 1, 2, 3, and 4 represent missing values and the categories of None, Low, Medium, High, and Very High for the discretized variables, respectively. Due to high right skeweness, categories corresponding to high concentration levels have low frequencies. Values of the *Dinophysis* concentration level are missing at 20 time points among 55 points used for analysis. Sample correlations between the factors are relatively strong. Figures 2K,L shows scatterplots for some selected pairs of the factors.

For bacterial RNA samples, three depth-integrated (0, 5, and 10 ft) water samples were collected at a total of 55 time points between April 2014 and November 2015. Two or three samples are sequenced at each time point. The numbers of replicates at the time points are illustrated in Figure 1B. Microbial RNA in the samples was extracted for 16S rRNA sequencing. Post-processing of sequences was performed using the Quantitative Insights Into Microbial Ecology (QIIME 1.9.1) pipeline. A total of nearly 39,823 OTUs were obtained in data after removing singletons. We restricted our attention to OTUs that have greater than or equal to five counts on average. The rule leaves in the end *J* = 263 OTUs for the 150 samples for the analysis. A heatmap of the counts in the filtered data is shown in Figure 1. The primary goal of the study is to investigate the effects of physical and biological factors on abundance levels of OTUs, while accounting for baseline abundance levels of OTUs in samples.

## 3. Results

### 3.1. Simulation experiment: model fitting and comparison

To fit the proposed model for the simulated data designed in section 2.2, we specified hyperparameter values of the model as follows; for the Laplace prior of β_*j,p*_, we let *a*_λ_ = *b*_λ_ = 0.5 for a gamma prior of $\lambda_{j}^{2}$ (with mean *a*_λ_/*b*_λ_ and variance $a_{\lambda }/b_{\lambda }^{2}$) and *a*_σ_ = *b*_σ_ = 0.3 for an inverse gamma prior for common variances $\sigma_{j}^{2}$. For the regularizing priors of ${\tilde{r}}_{t_{i},k}$ and ${\tilde{\alpha }}_{0,j}$, we fixed *d*_α_ = *d*_*r*_ = 10, *a*_*r*_ = *b*_*r*_ = *a*_α_ = *b*_α_ = 1, $\omega_{r}^{2}=\omega_{\alpha }^{2}=1.0$, $v_{r}^{2}=1$, and $v_{\alpha }^{2}=2.0$. We also fixed the number of mixture components for the regularizing priors *L*_*r*_ = 30 and *L*_α_ = 50. To specify values of the mean constraints *c*_*r*_ and *c*_α_, we took an empirical approach. We used the simulated *y*_*t*_*i*_,*k, j*_, computed estimates of *r*_*t*_*i*_,*k, j*_ and α_*j*, 0_ as described in section 2.2 and fixed the mean constraints at the means of the logarithm of the estimates, respectively. Note that the specified values of *c*_*r*_ and *c*_α_ were very different from the means of their true values. For the process convolution prior of OTU-time factor ${\tilde{\alpha }}_{t_{i},j}$, we chose a value of *M* such that the kernel function at a basis point is not entirely located in a place where no sample is obtained. We let the number of basis *M* = 13 and basis *u*_*m*_, *m* = 1, …, *M* evenly spaced between −10 and *T*_*i*_ + 10. For overdispersion parameter *s*_*j*_ we let *a*_*s*_ = 1 and *b*_*s*_ = 2. To run MCMC simulation, we initialized the parameters by simulating with their prior distributions. We then implemented posterior inference using MCMC simulation over 25,000 iterations, discarding the first 10,000 iterations as burn-in and choosing every other sample as thinning.

**Figure 3:**
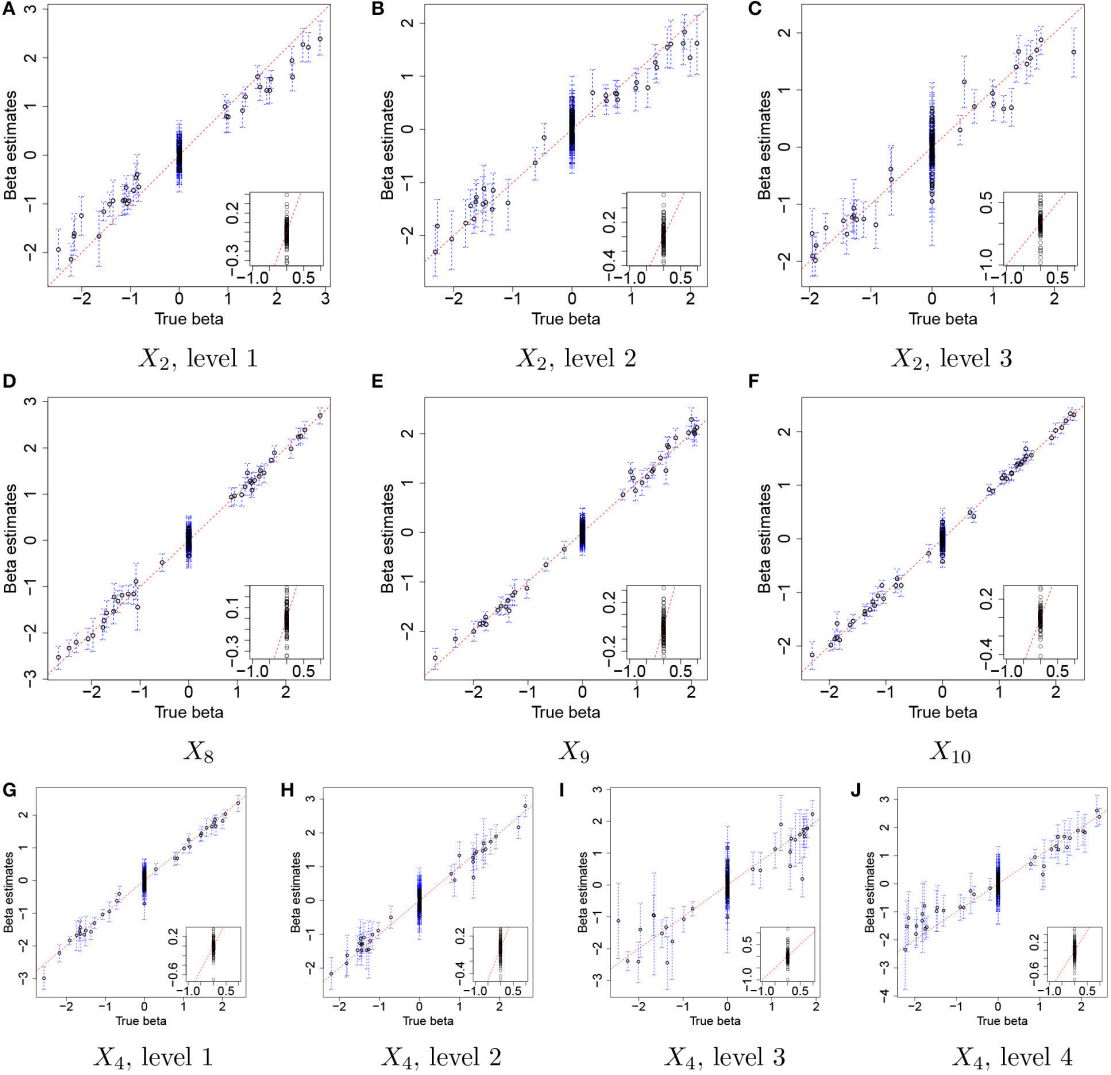
Simulation 1—proposed model. Comparison of the true values $\beta_{j,p}^{\text{TR}}$ and the posterior estimates of β_*j,p*_ under the proposed model for some selected covariates. Dots and blue dashed lines represent estimates of posterior means ${\hat{\beta }}_{j,p}$ of β_*j,p*_ and 95% credible intervals (CIs) of β_*j,p*_, respectively. The insert plot in each panel is a scatter plot of ${\hat{\beta }}_{j,p}$ and $\beta_{j,p}^{\text{TR}}$ for (*j,p*) with $\beta_{j,p}^{\text{TR}}=0$.

Figure 3 illustrates the comparison of posterior estimates of β_*j,p*_ to their true values $\beta_{j,p}^{\text{TR}}$ for some selected covariates. In the figure, dots and blue dashed lines represent posterior means ${\hat{\beta }}_{j,p}$ of β_*j,p*_ and their 95% credible intervals, respectively. ${\hat{\beta }}_{j,p}$s are around the 45 degree line (red dotted line) for most of (*j,p*) and most of the interval estimates captures the true values. It implies that the proposed model reasonably recovers $\beta_{j,p}^{\text{TR}}$. For categories 3 and 4 of *X*_4_ in Figures 3I,J, the credible intervals tend to be wider due to their low frequencies in the data as shown in Figure 2D. The insert plot in each panel illustrates a scatter plot of ${\hat{\beta }}_{j,p}$ for (*j,p*) with $\beta_{j,p}^{\text{TR}}=0$. It shows that the proposed regression model with the Laplace prior effectively shrinks β_*j,p*_ with $\beta_{j,p}^{\text{TR}}=0$ to zero, as is desired in our simulation setup. Supplementary Figure 1 has plots for all covariates.

**Figure 4:**
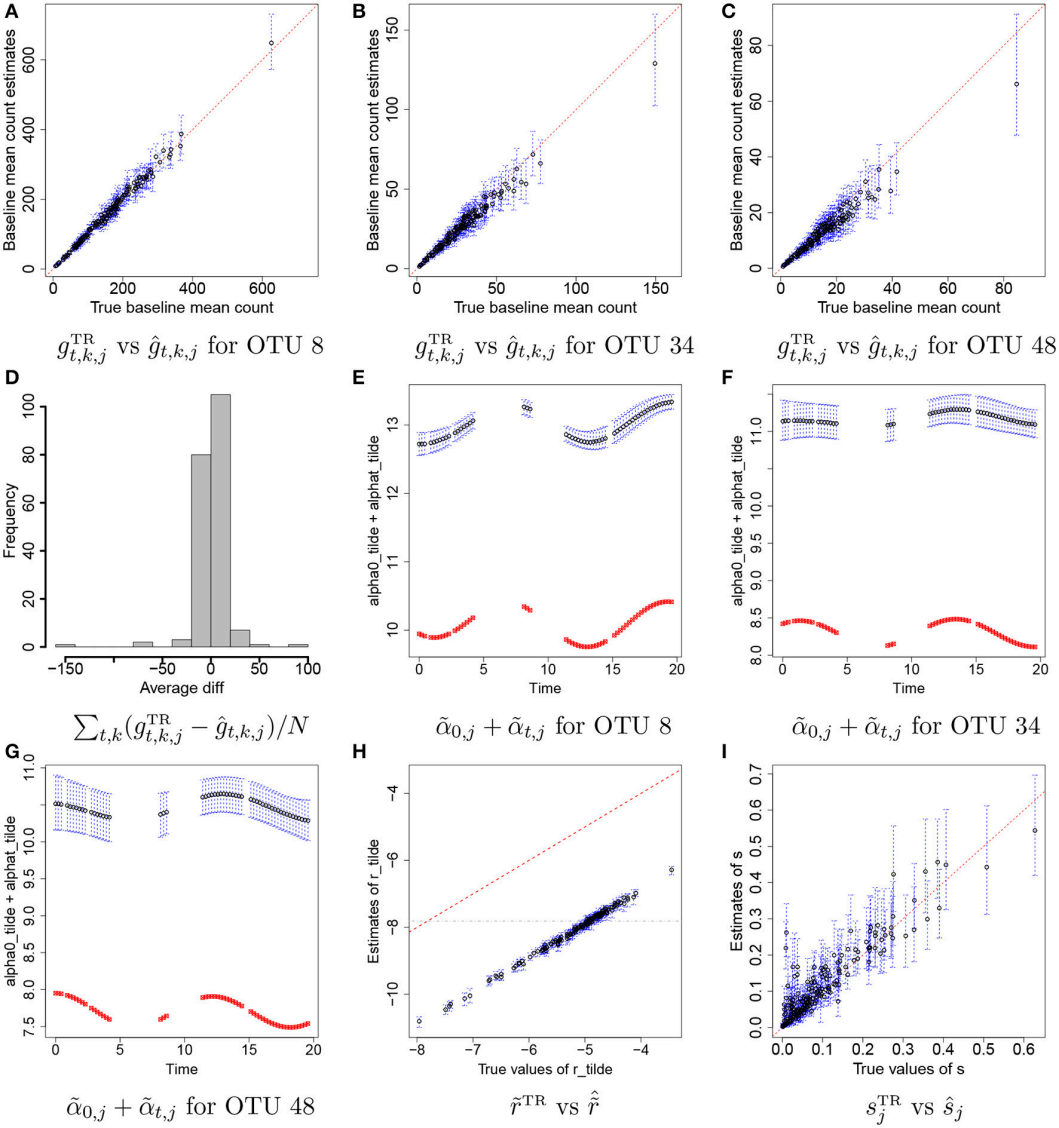
Simulation 1—proposed model. Panels **(A–C)** illustrate plots of the true baseline mean counts $g_{t,k,j}^{\text{TR}}$ vs their estimates ĝ_*t,k,j*_ for some selected OTUs *j* = 8, 34, 48. Panel **(D)** shows a histogram of averaged differences between $g_{t,k,j}^{\text{TR}}$ and ĝ_*t,k,j*_ for each OTU. Panels **(E–G)** show plots of estimates of ${\tilde{\alpha }}_{0,j}+{\tilde{\alpha }}_{t,j}$ over time for OTUs *j* = 8, 34, 48. Panel **(H)** has a scatterplot of ${\hat{\tilde{r}}}_{t,k}$ vs. ${\tilde{r}}^{\text{TR}}$. Panel **(I)** has a scatterplot of ŝ vs. *s*^TR^. Dots represent posterior mean estimates and blue vertical dotted lines 95% credible intervals. Red squares represent the true values.

Figures 4A–C illustrate plots of $g_{t,k,j}^{\text{TR}}$ vs estimates of *g*_*t,k,j*_ with their means (black dots) and 95% credible intervals (blue vertical lines) for some selected OTUs, *j* = 8, 34, and 48. Recall that we do not attempt to recover the true values of individual *r*_*t*_*i*_,*k*_, α_0, *j*_, and α_*t,j*_, but we rather aim to reasonably recover the true baseline mean counts, $g_{t_{i},k,j}^{\text{TR}}=r_{t_{i},k}^{\text{TR}}\alpha_{0,j}^{\text{TR}}\exp\left({\tilde{\alpha }}_{t_{i},j}^{\text{TR}}\right)$. In the figure the estimates are tightly around the 45 degree line, providing evidence that reasonable estimates of baseline mean counts are obtained under the proposed model. Figure 4D has a histogram of averaged differences between baseline mean count estimates and their true values, $D_{j}=\overset{n}{\underset{i=1}{\sum }}\overset{K_{i}}{\underset{k=1}{\sum }}\left({ĝ}_{t_{i},k,j}-g_{t_{i},k,j}^{\text{TR}}\right)/N$. The averaged differences are around zero, implying that the proposed model provides reasonable estimates of baseline mean counts for most of OTUs. We further examined individual parameters. Figures 4E–G shows the comparison of estimates of ${\tilde{\alpha }}_{0,j}+{\tilde{\alpha }}_{t,j}$ to their true values over time for the same OTUs in Figures 4A–C. Black dots and blue vertical lines represent estimates of posterior means of ${\tilde{\alpha }}_{0,j}+{\tilde{\alpha }}_{t,j}$ and their 95% credible intervals, respectively. Red squares represent their true values. From the figure, the estimates of ${\tilde{\alpha }}_{0,j}+{\tilde{\alpha }}_{t,j}$ are consistently greater than their true values at all time points, but capture their overall temporal trend. Figure 4H illustrates a scatterplot of ${\tilde{r}}_{t,k}^{\text{TR}}$ and their posterior estimates of ${\tilde{r}}_{t,k}$, where dots and blue vertical intervals denote estimates of posterior means and 95% credible intervals, respectively, and the gray horizontal line is at *c*_*r*_ used for analysis. Different from the estimates of ${\tilde{\alpha }}_{0,j}+{\tilde{\alpha }}_{t,j}$, the estimates of ${\tilde{r}}_{t,k}$ fall below the 45 degree line approximately by the same distance for all OTUs. It shows that estimates of ${\tilde{\alpha }}_{0,j}+{\tilde{\alpha }}_{t,j}$ and ${\tilde{r}}_{t,k}$ have discrepancies from their true values but in the opposite direction and the model can produce reasonable estimates of *g*_*t,k,j*_ as seen in Figures 4A–D. The true overdispersion parameters $s_{j}^{\text{TR}}$ are reasonably well estimated as shown in Figure 4I. We check the posterior predictive distribution of *Y*_*t,k,j*_. The posterior predicted values of *Y*_*t*_*i*_,*k, j*_ with their 95% predictive intervals for OTUs *j* = 8, 34, and 48 are compared to their observed values in Supplementary Figure 2. The figure indicates a reasonable model fit.

In addition, we conducted a sensitivity analysis to the specification of mean constraints *c*_*r*_ and *c*_α_ for the priors of $\tilde{r}$ and ${\tilde{\alpha }}_{0}$. We used different values for *c*_*r*_ and *c*_α_ and compared the estimates of *g*_*t,k,j*_ to their truth. Supplementary Figures 3a–c has histograms of averaged differences *D*_*j*_ between ĝ_*t*_*i*_,*k, j*_ and $g_{t_{i},k,j}^{\text{TR}}$ for different specification of *c*_*r*_ and *c*_α_. The histograms show minor change in estimates of *g*_*t*_*i*_,*k,j*_ under different specifications of *c*_*r*_ and *c*_α_. An sensitivity analysis to the specification of the number *M* of basis points in the GP convolution model for ${\tilde{\alpha }}_{t,j}$ was also performed. We used *M* = 8, 13, and 18 and examined estimates of the baseline mean counts, *g*_*t,k,j*_. Supplementary Figures 3a,d,e has histograms of averaged differences *D*_*j*_ for each of *M*. The results indicate that the baseline mean counts are reasonably estimated for a range of values of *M* in the simulation study.

**Figure 5:**
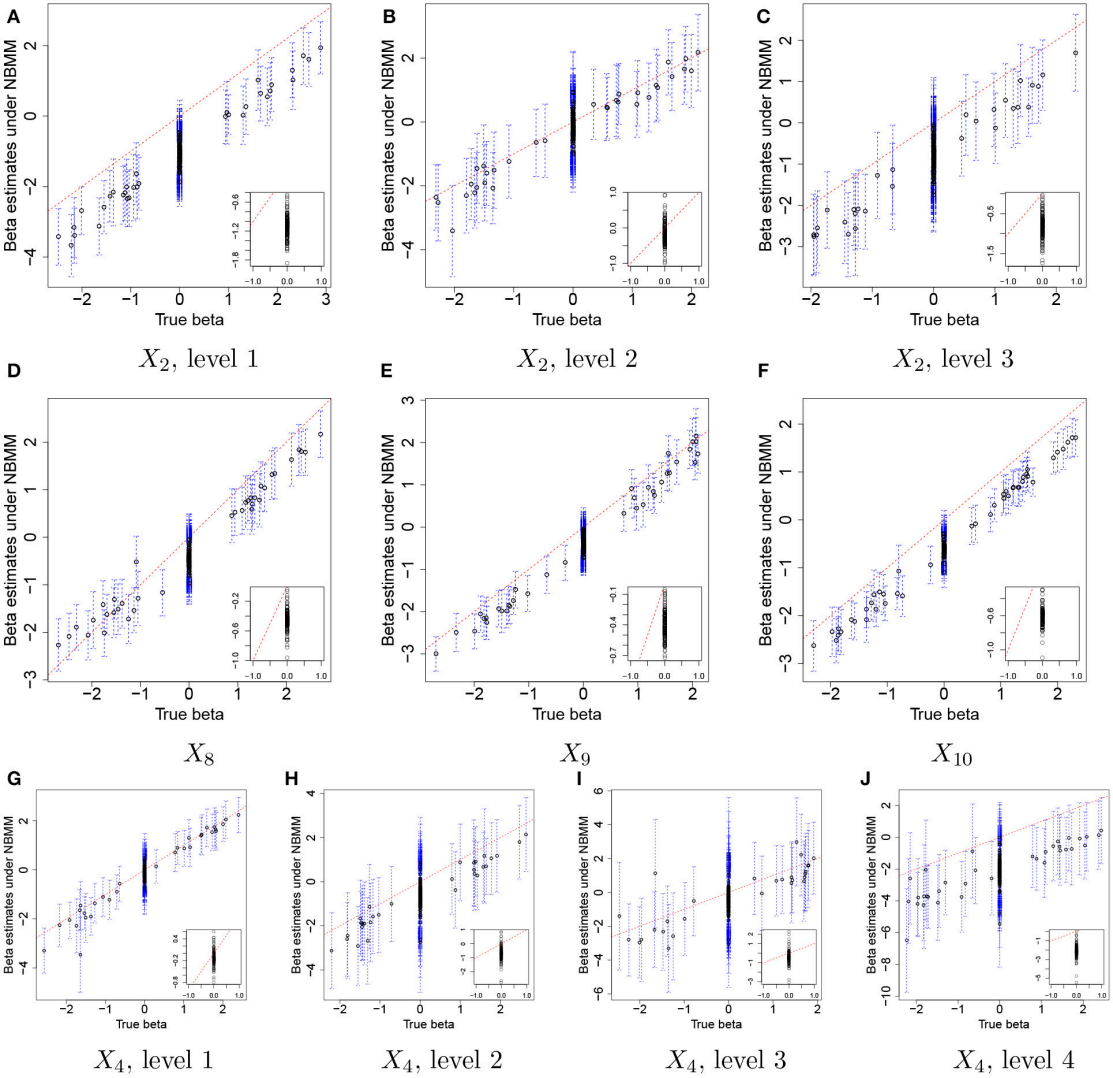
Simulation 1—NBMM. Comparison of the true values $\beta_{j,p}^{\text{TR}}$ and maximum likelihood estimates ${\hat{\beta }}_{j,p}^{\text{NBMM}}$ of β_*j,p*_ under the negative binomial mixed model (NBMM) for some selected covariates. Dots and blue dashed lines represent ${\hat{\beta }}^{\text{NBMM}}$ and their 95% confidence intervals, respectively. The insert plot in each panel is a scatter plot of ${\hat{\beta }}_{j,p}^{\text{NBMM}}$ and $\beta_{j,p}^{\text{TR}}$ for (*j,p*) with $\beta_{j,p}^{\text{TR}}=0$.

For comparison, we used the NBMM to the simulated data. Since the NBMM does not accommodate missing covariates, we used **X**^TR^ to fit the NBMM. Figure 5 compares the MLEs ${\hat{\beta }}_{j,p}^{\text{NBMM}}$ of β_*j,p*_ to the true values for the same covariates used in Figure 3. Dots and blue vertical lines represent the MLEs under the NBMM and their 95% confidence intervals, respectively. Comparing Figure 5 to Figure 3, the NBMM produces poor estimates. The MLEs are biased for some covariates (e.g., Figure 5A). Also, confidence intervals under the NBMM often fail to capture the true values and their interval estimates under the NBMM tend to be much wider than those under the proposed model. Normalization through observed sample total counts and inducing correlation in replicates through iid (independent and identically distributed) random effects under the NBMM may lead to poor estimation of the baseline mean abundance for the simulated data, resulting in deterioration of coefficient estimation. In addition, separate analyses of OTUs under the NBMM do not allow to strengthen estimates through combining information across OTUs. Comparing the insert plots in Figure 5 to those in Figure 3, ${\hat{\beta }}_{j,p}^{\text{NBMM}}$ with $\beta_{j,p}^{\text{TR}}=0$ tends to more widely spread out from zero and often their confidence intervals fail to capture zero. Supplementary Figure 4 has plots of β_*j,p*_ for all covariates. Supplementary Figures 4,5 shows the comparison of the estimates ${\hat{\theta }}^{\text{NBMM}}$ of overdispersion parameters under the NBMM to their true values. Note that θ^NBMM^ is the inverse of *s* in our model. The NBMM underestimates *s*_*j*_ for many OTUs, and yields poor predicted values, implying the lack of a fit.

We further examined the performance of the proposed model through additional simulation studies, Simulations 2 and 3 in Supplementary section 2. In these simulations, we studied the robustness of the model when different simulation setups are used to simulate data. In Simulation 2, we assumed no temporal dependence among OTU abundance and generated independent samples from a normal distribution for ${\tilde{\alpha }}_{t,j}$. We assumed that all $\beta_{j,p}^{\text{TR}}$ has nonzero effects for all OTUs and simulated β_*j,p*_ from a mixture of normals. The performance of our model is almost the same as in Simulation 1 (see Supplementary Figures 6–8). Interestingly, the NBMM that assumes iid random effects performs poorly for β estimation. In Simulation 3, we simulated ${\tilde{\alpha }}_{t,j}^{\text{TR}}$ from a discontinuous function. The results show that when the temporal dependence pattern is not smooth as assumed for the GP, estimates of the baseline mean counts under the proposed model are slightly deteriorated but the model produces reasonable inference on β_*j,p*_ (see Supplementary Figures 9–11). A more detailed summary of the additional simulations is given in Supplementary section 2.

### 3.2. Ocean microbiome data: model fitting and comparison

**Figure 6:**
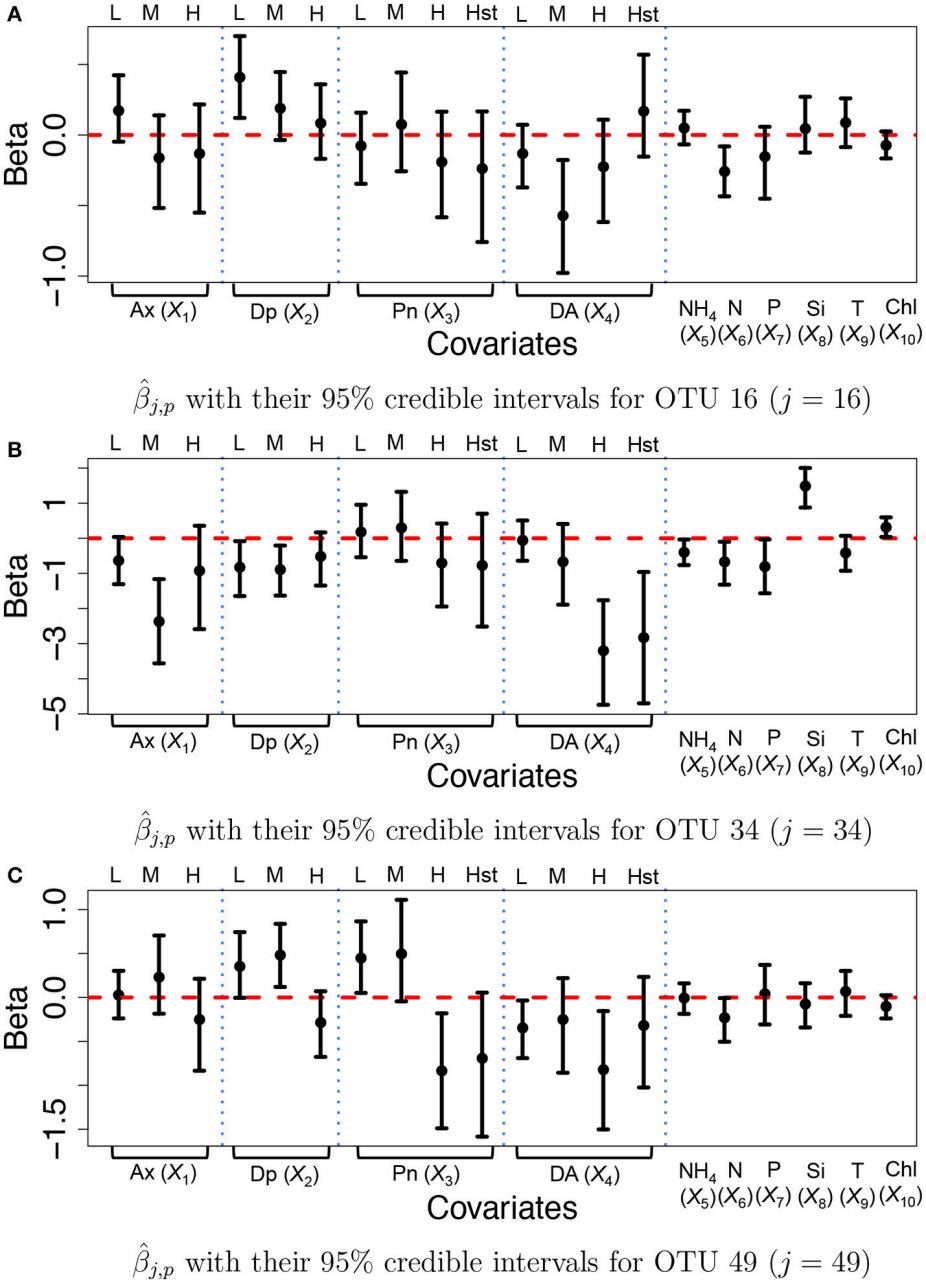
Ocean microbiome data—proposed model. Posterior Inference on β_*j*_ for some selected OTUs (*j* = 16, 34, 49). Dots represent the posterior means ${\hat{\beta }}_{j,p}$ of β_*j,p*_. Each vertical line connects the lower bounds and the upper bounds of 95% credible intervals.

We specified hyperparameters similar to those in the simulations and analyzed the microbiome data in section 2.3. The MCMC simulation was run over 25,000 iterations. The first 15,000 iterations were discarded as burn-in and every other sample was kept as thinning and used for inference. Figure 6 illustrates inference on covariate effects for some selected OTUs, *j* = 16, 34, and 49, taxonomically belonging to *Alteromonadales, Halomonas* sp., and *Alteromonadales* in the Gamma-proteobacteria phyla, respectively. Dots and vertical solid lines represent the posterior mean ${\hat{\beta }}_{j,p}$ and 95% credible interval estimates, respectively. Similar to the results of the simulation study, the credible intervals for high and highest levels of the discretized covariates tend to be wider due to their low frequencies in the data. From Figure 6A, on average the medium concentration level of domoic acid (DA, *X*_4_) and the concentration level of nitrate (N, *X*_6_) significantly decrease the mean abundance of OTU 16 by a multiplicative factor of exp(−0.572) = 0.564 and exp(−0.260) = 0.771, respectively. One may infer that the medium concentration level of domoic acid is significantly associated with lower expected counts for the OTU compared to those with category none of the domoic acid concentration level. A similar argument can be applied to the inference on the nitrate concentration level. Interestingly, we observed statistically significant reduction in abundance from many OTUs belonging to Gamma-proteobacteria including those OTUs for increasing domoic acid concentration (not shown). The resulting inference was further validated through a lab experiment. Most notably, four bacterial cultured isolates belonging to Gamma-proteobacteria (three among them are *Alteromonadales*) were observed to be severely retarded in growth after 2 days of exposure to increasing domoic acid of 0 to 150 μg/ml in the experiment (Sison-Mangus, unpublished data). This demonstrates that the proposed model successfully identifies important OTUs in ocean bacterial community dynamics for further investigation. More results are presented in Supplementary Section 3. Supplementary Figures 12a–c illustrates the posterior estimates of baseline expected counts ${\tilde{\alpha }}_{0,j}+{\tilde{\alpha }}_{t,j}$ normalized by sample size factors for the OTUs. From the figure, the baseline expected counts vary over time for those OTUs and the temporal pattern of OTU *j* = 34 is different from those of OTUs *j* = 16 and 49. Histograms of the posterior mean estimates ${\hat{\beta }}_{j,p}$ of β_*j,p*_, are illustrated in Supplementary Figure 13. The figure does not show clear overall tendency in the direction of association between covariates and OTU counts. Posterior inference on sample size factors *r*_*t*_*i*_,*k*_ and OTU specific overdispersion parameters *s*_*j*_ is illustrated in Supplementary Figures 12d,e.

**Figure 7:**
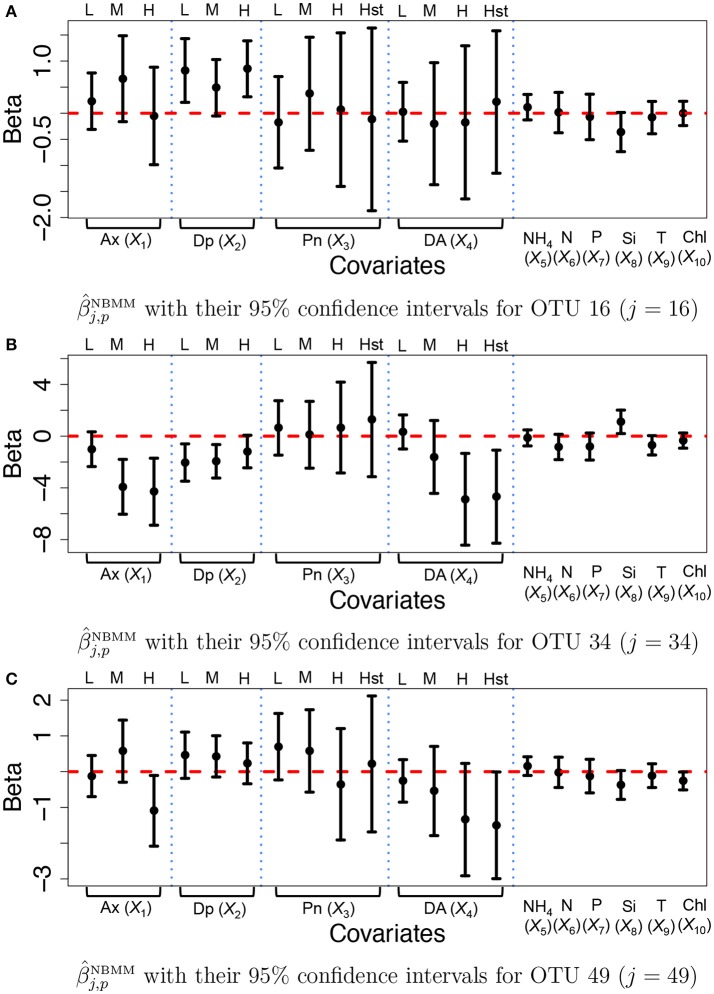
Ocean microbiome data—NBMM. Inference on β_*j*_ for some selected OTUs (*j* = 16, 34, 49) under the negative binomial mixed model (NBMM). Dots represent the maximum likelihood estimates ${\hat{\beta }}_{j,p}^{\text{NBMM}}$ of β_*j,p*_. Each vertical line connects the lower bounds and the upper bounds of 95% confidence intervals.

For comparison, we fitted the NBMM to the data. Since the NBMM does not account for missing values, we use the maximum a posteriori estimates of the missing values under the proposed for the NBMM. We used the R function *glmm* and the algorithm produced warning messages on convergence for 32 OTUs. Figure 7 illustrates ${\hat{\beta }}_{j,p}^{\text{NBMM}}$ (dots) with their 95% confidence intervals (solid vertical lines) for OTUs *j* = 16, 34, and 49. Inference on the covariate effects is different from that under the proposed. For example, domoic acid (DA) levels do not have significant effects on the mean counts for OTU *j* = 16 and 49 from Figures 7A,C. Comparing Figures 7A,C to Figure 7, the NBMM produces wider interval estimates for β_*j,p*_. Histograms of the MLEs of β_*j,p*_, ${\hat{\beta }}_{j,p}^{\text{NBMM}}$ under the NBMM are shown in Supplementary Figure 14. The histograms are much dispersed than those under the proposed model shown in Supplementary Figure 13. Estimates ${\hat{\beta }}_{j,p}$ and ${\hat{\beta }}_{j,p}^{\text{NBMM}}$ for all covariates are also compared in Supplementary Figure 15. From the figure, the NBMM yields extremely large or small values for ${\hat{\beta }}_{j,p}$ for some OTUs, possibly due to the convergence problem. The insert plots show that for regions of small values of ${\hat{\beta }}_{j,p}$, the estimates under the proposed are more shrunken toward zero than those under the NBMM, similar to the results in section 3.1. The overdispersion parameter estimates under the NBMM tend to be smaller than those under the proposed (shown in Supplementary Figure 12f), which may lead to different predictive distributions of OTU counts.

## 4. Discussion and conclusions

In this paper, we developed a Bayesian semiparametric regression model for joint analysis of microbiome data. We formulated the mean counts of OTUs as a product of factors and built models for the factors. We utilized the regularizing priors with mean constraints to avoid possible idenfiability issues, and the process convolution model to capture the temporal dependence structure in the baseline mean abundance of an OTU. The flexible model developed for baseline abundance enables joint analysis of all OTUs in the data and allows borrowing information across OTUs, across samples, and across time points. The model produces accurate estimates of the baseline mean counts and yields improved estimates of the effects of the covariates. We incorporated the Laplace distribution, a sparsity inducing shrinkage prior for the coefficients and the proposed model produces sparse estimates that is more desirable when the problem is high-dimensional and covariates are highly correlated. We compared the proposed model to a comparable frequentist model that does separate analyses for individual OTUs. The comparisons through the simulation study and real data analysis show better performance of the proposed model.

Although we focused on the analysis of NGS count data, the proposed model is quite general and can be applied for analyses of any count data. Future work will explore alternative approaches to model the effects of covariates on the mean counts. For example, one may consider a nonparametric model using linear combinations of basis functions (Kohn et al., 2001) to flexibly capture shape in the response function. In such a case, an elaborate development of the prior model may be needed to produce a robust inference since both the baseline mean counts and the covariate effects are nonparametrically modeled. Other possible extensions are to include a variable selection method such as a stochastic search variable selection (George and McCulloch, 1993) if it is reasonable to assume that some covariate effects are exactly zero, and to let coefficients vary over time if covariate effects evolve with time. For time varying coefficients, we may use the random walk process in Leybourne (1993) to induce relationship between β_*j,p,t*−1_ and β_*j,p,t*_. Considering the high dimensionality in OTU data, posterior computation may need to be carefully handled. Also, prior information may be needed to produce sensible inference due to sparsity in data.

## Author contributions

JL developed the statistical model and conducted simulation studies and data analysis. She also prepared the first draft and led the collaboration with MS-M for statistical analysis. MS-M provided the ocean microbiome data, participated the statistical model development, provided biological interpretation of the resulting inference and edited the manuscript.

### Conflict of interest statement

The authors declare that the research was conducted in the absence of any commercial or financial relationships that could be construed as a potential conflict of interest.
