# Supplementary material for: A Bayesian Semiparametric Regression Model for Joint Analysis of Microbiome Data
Source: Front Microbiol. 2018 Mar 26;9:522. doi: 10.3389/fmicb.2018.00522 (PMC5879107; doi:10.3389/fmicb.2018.00522)
Supplement: Supplementary file 1 [file Presentation1.PDF]

Supplementary Materials: A Bayesian Semiparametric Regression Model for Joint Analysis of Microbiome Data

1 Posterior Computation

Recall that $\tilde{\boldsymbol{\theta}} = (\beta, \sigma, \lambda^2, \phi, \tilde{r}, \psi^r, w^r, \eta^r, \tilde{\alpha}, \psi^\alpha, w^\alpha, \eta^\alpha, \theta, \tau^2, s)$ denotes the vector of all random parameters. We use the Markov chain Monte Carlo (MCMC) simulation to draw a sample of random parameters $\tilde{\boldsymbol{\theta}}$ from the following posterior distribution.

$$\begin{aligned} p(\tilde{\boldsymbol{\theta}} \mid \boldsymbol{y}) &\propto \prod_{i=1}^{n}\prod_{k=1}^{K_i}\prod_{j=1}^{J} p(y_{t_i,k,j} \mid \mu_{t_i,k,j}, s_j) p(\tilde{\boldsymbol{\theta}}) \\ &= \prod_{i=1}^{n}\prod_{k=1}^{K_i}\prod_{j=1}^{J} \frac{\Gamma(y_{t_i,k,j} + s_j^{-1})}{y_{t_i,k,j}!\Gamma(s_j^{-1})} \left(\frac{\mu_{t_i,k,j} s_j}{1+\mu_{t_i,k,j} s_j}\right)^{y_{t_i,k,j}} \left(\frac{1}{1+\mu_{t_i,k,j} s_j}\right)^{s_j^{-1}} p(\tilde{\boldsymbol{\theta}}), \end{aligned}$$

where $\boldsymbol{y} = \{y_{t_i,k,j}\}$, $i = 1, \ldots, n$, $k = 1, \ldots, K_i$ and $j = 1, \ldots, J$. Recall that the parameters $\tilde{r}_{t_i,k}$ and $\tilde{\alpha}_{0,j}$ follow the mixture distributions with mean constraints;

$$\begin{aligned} \tilde{r}_{t_i,k} \mid \psi^r, \eta^r, w^r, v_r^2, c_r &\overset{iid}{\sim} \sum_{\ell=1}^{L^r} \psi_\ell^r \left\{ w_\ell^r \phi(\eta_\ell^r, v_r^2) + (1 - w_\ell^r) \phi\left(\frac{c_r - w_\ell^r \eta_\ell^r}{1 - w_\ell^r}, v_r^2\right)\right\}, \\ \tilde{\alpha}_{0,j} \mid \psi^\alpha, \eta^\alpha, w^\alpha, v_\alpha^2, c_\alpha &\overset{iid}{\sim} \sum_{\ell=1}^{L^\alpha} \psi_\ell^\alpha \left\{ w_\ell^\alpha \phi(\eta_\ell^\alpha, v_\alpha^2) + (1 - w_\ell^\alpha) \phi\left(\frac{c_\alpha - w_\ell^\alpha \eta_\ell^\alpha}{1 - w_\ell^\alpha}, v_\alpha^2\right)\right\}, \end{aligned}$$

where $\phi(\eta, v^2)$ is the probability density function of the normal distribution with mean η and variance v^2. The vectors $\boldsymbol{\psi}^r = (\psi^r_1, \ldots, \psi^r_{L^r})$ and $\boldsymbol{\psi}^\alpha = (\psi^\alpha_1, \ldots, \psi^\alpha_{L^r})$ are mixture weights with $\sum_{\ell=1}^{L^r} \psi^r_\ell = \sum_{\ell=1}^{L^\alpha} \psi^\alpha_\ell = 1$ where $0 < \psi^r_\ell < 1$ and $0 < \psi^\alpha_\ell < 1$. We also have constraints $0 < w^r_\ell < 1$ and $0 < w^\alpha_\ell < 1$ for all ℓ. For easy posterior computation, we introduce vectors of mixture component indicators for sample size factor $\tilde{r}_{t_i,k}$ and OTU baseline factor $\tilde{\alpha}_{0,j}$. We let $\boldsymbol{\delta}^r_{t_i,k} = (\delta^r_{t_i,k,1}, \delta^r_{t_i,k,2})$ where $\delta^r_{t_i,k,1} = \ell$ with probability ψ^r_ℓ for $\delta^r_{t_i,k,1} \in \{1, \ldots, L^r\}$ and $\delta^r_{t_i,k,2} = 0$ with probability w^r_ℓ for $\delta^r_{t_i,k,2} \in \{0, 1\}$. Conditional on the indicator vector $\boldsymbol{\delta}^r_{t_i,k}$, we can write the prior distribution of $\tilde{r}_{t_i,k}$ as follows;

$$\tilde{r}_{t_i,k} \mid \eta^r, v^2_r, c_r, \delta^r_{t_i,k,1} = \ell \overset{indep}{\sim} \begin{cases} \mathrm{N}(\eta^r_\ell, v^2_r) & \text{if } \delta^r_{t_i,k,2} = 0, \\ \mathrm{N}\left(\frac{c_r - w^r_\ell \eta^r_\ell}{1 - w^r_\ell}, v^2_r\right) & \text{if } \delta^r_{t_i,k,2} = 1, \end{cases}$$

where $\mathrm{N}(a, b^2)$ is the normal distribution with mean a and variance b^2. Similarly, we define $\boldsymbol{\delta}^\alpha_j = (\delta^\alpha_{j,1}, \delta^\alpha_{j,2})$ where $\delta^\alpha_{j,1} = \ell$ with probability ψ^α_ℓ for $\delta^\alpha_{j,1} \in \{1, \ldots, L^\alpha\}$ and $\delta^\alpha_{j,2} = 0$ with probability w^α_ℓ for $\delta^\alpha_{j,2} \in \{0, 1\}$. Conditional on the indicator vector $\boldsymbol{\delta}^\alpha_{0,j}$, we write the prior distribution of $\tilde{\alpha}_{0,j}$ as follows;

$$\tilde{\alpha}_{0,j} \mid \eta^\alpha, v^2_\alpha, c_\alpha, \delta^\alpha_{j,1} = \ell \overset{indep}{\sim} \begin{cases} \mathrm{N}(\eta^\alpha_\ell, v^2_\alpha) & \text{if } \delta^\alpha_{j,2} = 0, \\ \mathrm{N}\left(\frac{c_\alpha - w^\alpha_\ell \eta^\alpha_\ell}{1 - w^\alpha_\ell}, v^2_\alpha\right) & \text{if } \delta^\alpha_{j,2} = 1. \end{cases}$$

We augment the vector of all random parameters including $\boldsymbol{\delta}^r$ and $\boldsymbol{\delta}^\alpha$, $\tilde{\boldsymbol{\theta}}' = \{\tilde{\boldsymbol{\theta}}, \boldsymbol{\delta}^r, \boldsymbol{\delta}^\alpha\}$. The parameters are estimated by iteratively drawing samples from the full conditional distributions given data and the other parameters.

1. Full conditional of s_j

Let $\tilde{\boldsymbol{\theta}}'_{-s_j}$ denote a vector of all random parameters but s_j. For $j = 1, \ldots, J$,

$$\begin{aligned} p(s_j \mid \boldsymbol{y}, \boldsymbol{u}, \tilde{\boldsymbol{\theta}}'_{-s_j}) &\propto \prod_{i=1}^{n} \prod_{k=1}^{K_i} \frac{\Gamma(y_{t_i,k,j} + s_j^{-1})}{\Gamma(s_j^{-1})} \left(\frac{\mu_{t_i,k,j} s_j}{1 + \mu_{t_i,k,j} s_j} \right)^{y_{t_i,k,j}} \left(\frac{1}{1 + \mu_{t_i,k,j} s_j} \right)^{s_j^{-1}} \\ &\quad \times p(s_j \mid a_s, b_s) \\ &\propto \prod_{i=1}^{n} \prod_{k=1}^{K_i} \frac{\Gamma(y_{t_i,k,j} + s_j^{-1})}{\Gamma(s_j^{-1})} \left(\frac{\mu_{t_i,k,j} s_j}{1 + \mu_{t_i,k,j} s_j} \right)^{y_{t_i,k,j}} \left(\frac{1}{1 + \mu_{t_i,k,j} s_j} \right)^{s_j^{-1}} \\ &\quad \times s_j^{a_j - 1} \exp(-b_s s_j). \end{aligned}$$

2. Full conditional of $\boldsymbol{\delta}^r_{t_i,k}$

   Let $\tilde{\boldsymbol{\theta}}'_{-\boldsymbol{\delta}^r_{t_i,k}}$ denote a vector of all random parameters but $\boldsymbol{\delta}^r_{t_i,k}$. For $i = 1, \ldots, n$ and $k = 1, \ldots, K_i$,

$$p(\boldsymbol{\delta}^r_{t_i,k} \mid \boldsymbol{y}, \tilde{\boldsymbol{\theta}}'_{-\boldsymbol{\delta}^r_{t_i,k}}) = p(\boldsymbol{\delta}^r_{t_i,k} \mid \tilde{\boldsymbol{\theta}}'_{-\boldsymbol{\delta}^r_{t_i,k}}).$$

   We thus have

$$\begin{aligned} p(\delta^r_{t_i,k,1} = \ell, \delta^r_{t_i,k,2} = 0 \mid \boldsymbol{y}, \tilde{\boldsymbol{\theta}}'_{-\boldsymbol{\delta}^r_{t_i,k}}) &\propto \psi^r_\ell w^r_\ell \exp\left(-\frac{(\tilde{r}_{t_i,k} - \eta^r_\ell)^2}{2v_r^2} \right), \\ p(\delta^r_{t_i,k,1} = \ell, \delta^r_{t_i,k,2} = 1 \mid \boldsymbol{y}, \tilde{\boldsymbol{\theta}}'_{-\boldsymbol{\delta}^r_{t_i,k}}) &\propto \psi^r_\ell (1 - w^r_\ell) \exp\left(-\frac{(\tilde{r}_{t_i,k} - \frac{c_r - w^r_\ell \eta^r_\ell}{1 - w^r_\ell})^2}{2v_r^2} \right), \end{aligned}$$

   where $\ell = 1, \ldots, L^r$.

3. Full conditional of $\tilde{r}_{t_i,k}$

   Let $\tilde{\boldsymbol{\theta}}'_{-\tilde{r}_{t_i,k}}$ denote a vector of all random parameters but $\tilde{r}_{t_i,k}$. For $i = 1, \ldots, n$ and

$k = 1, \ldots, K_i,$

$$
\begin{aligned}
p(\tilde{r}_{t_i,k} \mid \boldsymbol{y}, \tilde{\boldsymbol{\theta}}'_{-\tilde{r}_{t_i,k}}) &\propto \prod_{j=1}^{J} \left(\frac{\mu_{t_i,k,j} s_j}{1 + \mu_{t_i,k,j} s_j} \right)^{y_{t_i,k,j}} \left(\frac{1}{1 + \mu_{t_i,k,j} s_j} \right)^{s_j^{-1}} \times p(\tilde{r}_{t_i,k} \mid \tilde{\boldsymbol{\theta}}'_{-\tilde{r}_{t_i,k}}) \\
&\propto \prod_{j=1}^{J} \left(\frac{\mu_{t_i,k,j} s_j}{1 + \mu_{t_i,k,j} s_j} \right)^{y_{t_i,k,j}} \left(\frac{1}{1 + \mu_{t_i,k,j} s_j} \right)^{s_j^{-1}} \\
&\qquad \times \exp\Bigg\{ - \frac{(\tilde{r}_{t_i,k} - \eta^r_{\delta^r_{t_i,k,1}})^2}{2v_r^2} \times (1 - \delta^r_{t_i,k,2}) \\
&\qquad - \frac{(\tilde{r}_{t_i,k} - \frac{c_r - w^r_{\delta^r_{t_i,k,1}} \eta^r_{\delta^r_{t_i,k,1}}}{1 - w^r_{\delta^r_{t_i,k,1}}})^2}{2v_r^2} \times \delta^r_{t_i,k,2} \Bigg\}.
\end{aligned}
$$

4. Full conditional of $\boldsymbol{\psi}^r$

Let $\tilde{\boldsymbol{\theta}}'_{-\boldsymbol{\psi}^r}$ denote a vector of all random parameters but $\boldsymbol{\psi}^r$.

$$
\begin{aligned}
p(\boldsymbol{\psi}^r \mid \boldsymbol{y}, \tilde{\boldsymbol{\theta}}'_{-\boldsymbol{\psi}^r}) &= p(\boldsymbol{\psi}^r \mid \tilde{\boldsymbol{\theta}}'_{-\boldsymbol{\psi}^r}) \\
&\propto \prod_{\ell=1}^{L^r} (\psi^r_\ell)^{d^r_\ell + \sum_{i,k} 1(\delta^r_{t_i,k,1} = \ell) - 1},
\end{aligned}
$$

where $1(\cdot)$ is the indicator function,

$$
1(\delta^r_{t,k,1} = \ell) = \begin{cases} 1 & \text{if } \delta^r_{t,k,1} = \ell, \\ 0 & \text{otherwise.} \end{cases}
$$

5. Full conditional of w^r_ℓ

Let $\tilde{\boldsymbol{\theta}}'_{-w_\ell^r}$ denote a vector of all random parameters but w_ℓ^r. For $\ell = 1, \ldots, L^r$,

$$\begin{aligned} p(w_\ell^r \mid \boldsymbol{y}, \tilde{\boldsymbol{\theta}}'_{-w_\ell^r}) &= p(w_\ell^r \mid \tilde{\boldsymbol{\theta}}'_{-w_\ell^r}) \\ &\propto (w_\ell^r)^{a_w^r + \sum_{i,k} 1(\boldsymbol{\delta}^r_{t_i,k} = (\ell,0)) - 1} (1 - w_\ell^r)^{b_w^r + \sum_{i,k} 1(\boldsymbol{\delta}^r_{t_i,k} = (\ell,1)) - 1} \\ &\quad \times \prod_{i=1}^n \prod_{k=1}^{K_i} 1(\boldsymbol{\delta}^r_{t_i,k} = (\ell, 1)) \times \exp\left(- \frac{(\tilde{r}_{t_i,k} - \frac{c_r - w_\ell^r \eta_\ell^r}{1 - w_\ell^r})^2}{2v_r^2} \right). \end{aligned}$$

6. Full conditional of η_ℓ^r

Let $\tilde{\boldsymbol{\theta}}'_{-\eta_\ell^r}$ denote a vector of all random parameters but η_ℓ^r. For $\ell = 1, \ldots, L^r$,

$$\begin{aligned} p(\eta_\ell^r \mid \boldsymbol{y}, \tilde{\boldsymbol{\theta}}'_{-\eta_\ell^r}) &= p(\eta_\ell^r \mid \tilde{\boldsymbol{\theta}}'_{-\eta_\ell^r}) \\ &\propto \exp\left(- \frac{(\eta_\ell^r)^2}{2\omega_r^2} \right) \times \prod_{i=1}^n \prod_{k=1}^{K_i} \exp\Big\{ - \frac{(\tilde{r}_{t_i,k} - \eta^r_{\delta^r_{t_i,k,1}})^2}{2v_r^2} \times 1(\boldsymbol{\delta}^r_{t_i,k} = (\ell, 0)) \\ &\quad - \frac{(\tilde{r}_{t_i,k} - \frac{c_r - w^r_{\delta_{t_i,k}} \eta^r_{\delta^r_{t_i,k,1}}}{1 - w^r_{\delta^r_{t_i,k,1}}})^2}{2v_r^2} \times 1(\boldsymbol{\delta}^r_{t_i,k} = (\ell, 1)) \Big\}. \end{aligned}$$

7. Full conditional of $\boldsymbol{\delta}_j^\alpha$

Let $\tilde{\boldsymbol{\theta}}'_{-\boldsymbol{\delta}_j^\alpha}$ denote a vector of all random parameters but $\boldsymbol{\delta}_j^\alpha$. For $j = 1, \ldots, J$,

$$p(\boldsymbol{\delta}_j^\alpha \mid \boldsymbol{y}, \tilde{\boldsymbol{\theta}}'_{-\boldsymbol{\delta}_{0,j}^\alpha}) = p(\boldsymbol{\delta}_j^\alpha \mid \tilde{\boldsymbol{\theta}}'_{-\boldsymbol{\delta}_j^\alpha}).$$

We thus have

$$\begin{aligned} p(\delta_{j,1}^\alpha = \ell, \delta_{j,2}^\alpha = 0 \mid \boldsymbol{y}, \tilde{\boldsymbol{\theta}}'_{-\boldsymbol{\delta}_j^\alpha}) &\propto \psi_\ell^\alpha w_\ell^\alpha \exp\left(- \frac{(\tilde{\alpha}_{0,j} - \eta_\ell^\alpha)^2}{2v_\alpha^2} \right), \\ p(\delta_{j,1}^\alpha = \ell, \delta_{j,2}^\alpha = 1 \mid \boldsymbol{y}, \tilde{\boldsymbol{\theta}}'_{-\boldsymbol{\delta}_j^\alpha}) &\propto \psi_\ell^\alpha (1 - w_\ell^\alpha) \exp\left(- \frac{(\tilde{\alpha}_{0,j} - \frac{c_\alpha - w_\ell^\alpha \eta_\ell^\alpha}{1 - w_\ell^\alpha})^2}{2v_\alpha^2} \right), \end{aligned}$$

where $\ell = 1, \dots, L^\alpha$.

8. Full conditional of $\tilde{\alpha}_{0,j}$

   Let $\tilde{\boldsymbol{\theta}}'_{-\tilde{\alpha}_{0,j}}$ denote a vector of all random parameters but $\tilde{\alpha}_{0,j}$. For $j = 1, \dots, J$,

$$\begin{aligned} p(\tilde{\alpha}_{0,j} \mid \boldsymbol{y}, \tilde{\boldsymbol{\theta}}'_{-\tilde{\alpha}_{0,j}}) &\propto \prod_{i=1}^{n}\prod_{k=1}^{K_i}\left(\frac{\mu_{t_i,k,j}s_j}{1+\mu_{t_i,k,j}s_j}\right)^{y_{t_i,k,j}}\left(\frac{1}{1+\mu_{t_i,k,j}s_j}\right)^{s_j^{-1}} \\ &\quad \times p(\tilde{\alpha}_{0,j} \mid \tilde{\boldsymbol{\theta}}'_{-\tilde{\alpha}_{0,j}}) \\ &\propto \prod_{j=1}^{J}\left(\frac{\mu_{t_i,k,j}s_j}{1+\mu_{t_i,k,j}s_j}\right)^{y_{t_i,k,j}}\left(\frac{1}{1+\mu_{t_i,k,j}s_j}\right)^{s_j^{-1}} \\ &\quad \times \exp\left(-\frac{(\tilde{\alpha}_{0,j}-\eta^r_{\delta^\alpha_{j,1}})^2}{2v_\alpha^2}\times(1-\delta^\alpha_{j,2}) - \frac{(\tilde{\alpha}_{0,j}-\frac{c_\alpha - w^\alpha_{\delta^\alpha_{j,1}}\eta^r_{\delta^\alpha_{j,1}}}{1-w^\alpha_{\delta^\alpha_{j,1}}})^2}{2v_\alpha^2}\times\delta^\alpha_{j,2}\right). \end{aligned}$$

9. Full conditional of $\boldsymbol{\psi}^\alpha$

   Let $\tilde{\boldsymbol{\theta}}'_{-\boldsymbol{\psi}^\alpha}$ denote a vector of all random parameters but $\boldsymbol{\psi}^\alpha$.

$$\begin{aligned} p(\boldsymbol{\psi}^\alpha \mid \boldsymbol{y}, \tilde{\boldsymbol{\theta}}'_{-\boldsymbol{\psi}^\alpha}) &= p(\boldsymbol{\psi}^\alpha \mid \tilde{\boldsymbol{\theta}}'_{-\boldsymbol{\psi}^\alpha}) \\ &\propto \prod_{\ell=1}^{L^\alpha}(\psi^\alpha_\ell)^{d^\alpha_\ell + \sum_j 1(\delta^\alpha_{j,1}=\ell)-1}. \end{aligned}$$

10. Full conditional of w^α_ℓ

    Let $\tilde{\boldsymbol{\theta}}'_{-w^r_\ell}$ denote a vector of all random parameters but w^r_ℓ. For $\ell = 1, \dots, L^r$,

$$\begin{aligned} p(w^\alpha_\ell \mid \boldsymbol{y}, \boldsymbol{\theta}''_{-w^\alpha_\ell}) &= p(w^\alpha_\ell \mid \tilde{\boldsymbol{\theta}}'_{-w^\alpha_\ell}) \\ &\propto (w^\alpha_\ell)^{a^\alpha_w + \sum_{j=1}^J 1(\boldsymbol{\delta}^\alpha_j=(\ell,0))-1}(1-w^\alpha_\ell)^{b^\alpha_w+\sum_{j=1}^J 1(\boldsymbol{\delta}^\alpha_j=(\ell,1))-1} \\ &\quad \times \prod_{j=1}^{J} 1(\boldsymbol{\delta}^\alpha_j = (\ell,1)) \times \exp\left(-\frac{(\tilde{\alpha}_{0,j}-\frac{c_\alpha - w^\alpha_\ell\eta^\alpha_\ell}{1-w^\alpha_\ell})^2}{2v_\alpha^2}\right). \end{aligned}$$

11. Full conditional of η_ℓ^α

Let $\tilde{\boldsymbol{\theta}}'_{-\eta_\ell^\alpha}$ denote a vector of all random parameters but η_ℓ^α. For $\ell = 1, \ldots, L^\alpha$,

$$\begin{aligned} p(\eta_\ell^\alpha \mid \boldsymbol{y}, \tilde{\boldsymbol{\theta}}'_{-\eta_\ell^\alpha}) &= p(\eta_\ell^\alpha \mid \tilde{\boldsymbol{\theta}}'_{-\eta_\ell^\alpha}) \\ &\propto \exp\left(-\frac{(\eta_\ell^\alpha)^2}{2\omega_\alpha^2}\right) \times \prod_{j=1}^{J} \exp\Big\{ -\frac{(\tilde{\alpha}_{0,j} - \eta^\alpha_{\delta^\alpha_{j,1}})^2}{2v_\alpha^2} \times 1(\boldsymbol{\delta}_j^\alpha = (\ell, 0)) \\ &\qquad -\frac{(\tilde{\alpha}_{0,j} - \frac{c_\alpha - w^\alpha_{\delta^\alpha_{j,1}} \eta^\alpha_{\delta^\alpha_{j,1}}}{1 - w^\alpha_{\delta^\alpha_{j,1}}})^2}{2v_\alpha^2} \times 1(\boldsymbol{\delta}_j^\alpha = (\ell, 1))\Big\}. \end{aligned}$$

12. Full conditional of $\beta_{j,p}$

Let $\tilde{\boldsymbol{\theta}}'_{-\beta_{j,p}}$ denote a vector of all random parameters but $\beta_{j,p}$. For $j = 1, \ldots, J$ and $p = 1, \ldots, P$,

$$\begin{aligned} p(\beta_{j,p} \mid \boldsymbol{y}, \tilde{\boldsymbol{\theta}}'_{-\beta_{j,p}}) &\propto \prod_{i=1}^{n} \prod_{k=1}^{K_i} \left(\frac{\mu_{t_i,k,j} s_j}{1 + \mu_{t_i,k,j} s_j}\right)^{y_{t_i,k,j}} \left(\frac{1}{1 + \mu_{t_i,k,j} s_j}\right)^{s_j^{-1}} \times p(\beta_{j,p} \mid \sigma_j^2, \phi_{j,p}) \\ &\propto \prod_{i=1}^{n} \prod_{k=1}^{K_i} \left(\frac{\mu_{t_i,k,j} s_j}{1 + \mu_{t_i,k,j} s_j}\right)^{y_{t_i,k,j}} \left(\frac{1}{1 + \mu_{t_i,k,j} s_j}\right)^{s_j^{-1}} \exp\left(-\frac{\beta_{j,p}^2}{2\sigma_j^2, \phi_{j,p}}\right). \end{aligned}$$

13. Full conditional of σ_j^2

Let $\tilde{\boldsymbol{\theta}}'_{-\sigma_j^2}$ denote a vector of all random parameters but σ_j^2. For $j = 1, \ldots, J$,

$$\begin{aligned} p(\sigma_j^2 \mid \boldsymbol{y}, \tilde{\boldsymbol{\theta}}'_{-\sigma_j^2}) &= p(\sigma_j^2 \mid \tilde{\boldsymbol{\theta}}'_{-\sigma_j^2}) \\ &\propto \left(\sigma_j^2\right)^{-\frac{P}{2} - a_\sigma - 1} \exp\left(-\frac{1}{\sigma_j^2}\Big(\sum_{p=1}^{P} \frac{\beta_{j,p}^2}{2\phi_{j,p}} + b_\sigma\Big)\right). \end{aligned}$$

We thus sample σ_j^2 from $\mathrm{IG}(\frac{P}{2}+a_\sigma, \sum_{p=1}^{P}\frac{\beta_{j,p}^2}{2\phi_{j,p}}+b_\sigma)$.

14. Full conditional of λ_j^2

Let $\tilde{\boldsymbol{\theta}}'_{-\lambda_j^2}$ denote a vector of all random parameters but λ_j^2. For $j=1,\ldots,J$,

$$\begin{aligned} p(\lambda_j^2 \mid \boldsymbol{y}, \tilde{\boldsymbol{\theta}}'_{-\lambda_j}) &= p(\lambda_j^2 \mid \tilde{\boldsymbol{\theta}}'_{-\lambda_j^2}) \\ &\propto (\lambda_j^2)^{-P-a_\lambda-1}\exp\left(-\lambda_j^2(\sum_{p=1}^{P}\frac{\phi_{j,p}^2}{2}+b_\lambda)\right). \end{aligned}$$

We thus sample λ_j^2 from $\mathrm{Ga}(P+a_\lambda, \sum_{p=1}^{P}\frac{\phi_{j,p}^2}{2}+b_\lambda)$.

15. Full conditional of $\phi_{j,p}$

Let $\tilde{\boldsymbol{\theta}}'_{-\phi_{j,p}}$ denote a vector of all random parameters but $\phi_{j,p}$. For $j=1,\ldots,J$ and $p=1,\ldots,P$,

$$\begin{aligned} p(\phi_{j,p} \mid \boldsymbol{y}, \tilde{\boldsymbol{\theta}}'_{-\phi_{j,p}}) &= p(\phi_{j,p} \mid \tilde{\boldsymbol{\theta}}'_{-\phi_{j,p}}) \\ &\propto \phi_{j,p}^{-1/2}\exp\left(-\frac{\beta_{j,p}^2}{2\phi_{j,p}\sigma_j^2}-\frac{\lambda_j^2\phi_{j,p}}{2}\right). \end{aligned}$$

Let $\phi'_{j,p}=1/\phi_{j,p}$ and find that the full conditional distribution of $\phi'_{j,p}$ is the inverse Gaussian distribution with mean $\mu'_{j,p}$ and shape parameter λ'_j where $\lambda'_j=\lambda_j^2$ and $\mu'_{j,p}=\sqrt{\lambda'_j\sigma_j^2/\beta_{j,p}^2}$ (Park and Casella, 2008).

16. Full conditional of $\theta_{m,j}$

Let $\tilde{\boldsymbol{\theta}}'_{-\theta_{m,j}}$ denote a vector of all random parameters but $\theta_{m,j}$. For $j=1,\ldots,J$ and $m=1,\ldots,M$,

$$\begin{aligned} p(\theta_{m,j} \mid \boldsymbol{y}, \boldsymbol{u}, \tilde{\boldsymbol{\theta}}'_{-\theta_{m,j}}) &\propto \prod_{i=1}^{n}\prod_{k=1}^{K_i}\left(\frac{\mu_{t_i,k,j}s_j}{1+\mu_{t_i,k,j}s_j}\right)^{y_{t_i,k,j}}\left(\frac{1}{1+\mu_{t_i,k,j}s_j}\right)^{s_j^{-1}}\times p(s_j \mid a_s, b_s) \\ &\propto \prod_{i=1}^{n}\prod_{k=1}^{K_i}\frac{\Gamma(y_{t_i,k,j}+s_j^{-1})}{\Gamma(s_j^{-1})}\left(\frac{\mu_{t_i,k,j}s_j}{1+\mu_{t_i,k,j}s_j}\right)^{y_{t_i,k,j}}\left(\frac{1}{1+\mu_{t_i,k,j}s_j}\right)^{s_j^{-1}}\exp\left(-\frac{\theta_{m,j}^2}{2\tau_j^2}\right). \end{aligned}$$

17. Full conditional of τ_j^2

Let $\tilde{\boldsymbol{\theta}}'_{-\tau_j^2}$ denote a vector of all random parameters but τ_j^2. For $j = 1, \ldots, J$,

$$\begin{aligned} p(\tau_j^2 \mid \boldsymbol{y}, \tilde{\boldsymbol{\theta}}'_{-\tau_j^2}) &= p(\tau_j^2 \mid \tilde{\boldsymbol{\theta}}'_{-\tau_j^2}) \\ &\propto \left(\tau_j^2\right)^{-\frac{M}{2}-a_\tau-1} \exp\left(-\frac{1}{\tau_j^2}\left(\sum_{m=1}^{M} \frac{\theta_{m,j}^2}{2} + b_\tau\right)\right). \end{aligned}$$

We thus sample τ_j^2 from $\mathrm{IG}(\frac{M}{2} + a_\tau, \sum_{m=1}^{M} \frac{\theta_{m,j}^2}{2} + b_\tau)$.

2 Simulations

2.1 Additional Results for Simulation 1

We present additional results from Simulation 1 in §3.1 of the main text. Figure 1 compares the posterior inference on $\boldsymbol{\beta}$ under the proposed model to their truth $\boldsymbol{\beta}^{\mathrm{TR}}$ for all covariates. The dots and blue vertical lines represent the posterior means $\hat{\beta}_{j,p}$ and 95% credible intervals, respectively. The dots are close to the 45 degree line, implying that the point estimates are reasonably close to their true values. The credible intervals well capture the true values. Better inference is obtained for the coefficients for continuous covariates (X_5-X_{10}) and categories of the discretized covariates with high observed frequencies such as level 1 (low concentration level) of X_1, X_3 and X_4. Figure 2 shows the posterior prediction under the proposed model for OTUs $j = 8, 34$ and 48, where the dots and blue vertical lines are the posterior predicted values and 95% prediction intervals. The proposed model achieves a good fit from the figure. The estimates of $\boldsymbol{\beta}$ under the negative binomial mixed model (NBMM) in Zhang *et al.* (2017) are compared to the truth in Figure 4. The NBMM yields biased estimates for some covariates such as all levels of X_2 and X_4 and X_{10}. Comparing to

Figure 1, the interval estimates under the NBMM are much wider than those under the proposed model. Estimates of the overdispersion parameters under the NBMM are compared to the truth in Figure 5. Note that due to different formulation, θ_j^{NBMM} is $1/s_j^{\text{TR}}$. From the figure the estimates tend to be slightly smaller than the true values for many OTUs.

2.2 Simulation 2

We conducted a simulation for further examination of the proposed model and compared it to the NBMM. We kept the most of the simulation set-up in Simulation 1 in §2.2 of the main text, expect the distributions used to generate $\beta_{j,p}^{\text{TR}}$ and $\tilde{\alpha}_{t_i,j}^{\text{TR}}$. We assumed that all covariates have nonzero effect for all OTUs and simulated $\beta_{j,p}^{\text{TR}}$ from a mixture distribution, $\beta_{j,p} \stackrel{iid}{\sim} 0.5\text{N}(0, 0.3^2) + 0.25\text{N}(-1.5, 0.3^2) + 0.25\text{N}(1.5, 0.3^2)$. We let $\tilde{\alpha}_{t_i,j}^{\text{TR}} \stackrel{iid}{\sim} \text{N}(0, 0.1^2)$, which implies that there is no temporal dependence. The simulation set up is similar to the assumptions used for the NBMM.

The results under the proposed model are illustrated in Figures 6-7. From Figure 6 the proposed model yields reasonable estimates of the covariate effects. The posterior means $\hat{\beta}_{j,p}$ of $\beta_{j,p}$ and their 95% credible intervals (dots and blue dotted lines in the figures, respectively) are closely aligned along the 45 degree line. Figure 7(a) shows a histogram of averaged differences between baseline mean count estimates and their true values $D_j = \sum_{i=1}^{n} \sum_{k=1}^{K_i} (\hat{g}_{t_i,k,j} - g_{t_i,k,j}^{\text{TR}})/N$, where $\hat{g}_{t_i,k,j}$ is the posterior mean estimate of $g_{t_i,k,j}$. Recall that the model may not recover individual parameters of $\tilde{r}_{t,k} = \log(r_{t,k})$, $\tilde{\alpha}_{0,j}$ and $\tilde{\alpha}_{t,j}$ due to some identifiability issue but the baseline mean count $g_{t,k,j} = r_{t,k} \exp(\tilde{\alpha}_{0,j} + \tilde{\alpha}_{t,j})$ are reasonably estimated due to the regularizing prior with mean constraints. Panels (b)-(d) illustrate the posterior mean estimates of $\tilde{\alpha}_{0,j} + \tilde{\alpha}_{t,j}$ for some selected OTUs $j = 8$, 34, 48 and panel (e) shows a scatterplot of the posterior means of $\tilde{r}_{t,k}$ versus their true values. Similar to the results of Simulation 1, the estimates of $\tilde{\alpha}_{0,j} + \tilde{\alpha}_{t,j}$ and $\tilde{r}_{t,k}$ are consistently different from their truth in the opposite directions. From panels (g)-(i), when there is no temporal

dependence, the estimates of $\tilde{\alpha}_{t,j}$ by the Gaussian process convolution model almost stay constant over time. The estimates of $\boldsymbol{\beta}$ under the NBMM are compared to the truth in Figure 8. Similar to the results of Simulation 1, the NBMM produces biased estimates for some covariates such as all levels of discrete covaraites X_2 and X_6 and continuous covariate X_{10}. It also produces wider interval estimates than the proposed model.

2.3 Simulation 3

A simulation study was conducted to examine the performance of the proposed model when the true temporal pattern is discontinuous. While keeping the most of the simulation set-up in Simulation 1 in §2.2 of the main text, we simulated $\tilde{\alpha}_{t,j}^{\text{TR}}$ as follows;

$$\tilde{\alpha}_{t_i,j}^{\text{TR}} = a_{t_i,j}\cos(3\pi(\tilde{t}_i - b_{t_i,j})) + c_{t_i,j}(\tilde{t}_i - \tilde{t}^{\star})^2 + d_{t_i,j}. \tag{1}$$

Here $\tilde{t}_i$ and $\tilde{t}^{\star}$ denote time t_i in year and the median of $\tilde{t}_i$, respectively. We let OTUs to have different patterns by generating $a_{t,j} \overset{iid}{\sim} \text{N}(0.15, 0.1^2)$, $b_{t,j} \overset{iid}{\sim} \text{N}(0, 0.5^2)$, and $c_{t,j} \overset{iid}{\sim} \text{N}(0.1, 0.1^2)$. Note that the true temporal pattern is assumed to have a shorter cycle than that in Simulation 1. Also, different from the set up in Simulation 1, discontinuous patterns over time in $\alpha_{t,j}^{\text{TR}}$ are induced through a step function $d_{t_i,j}$ in (1). In particular, let $d_{t_i,j} = d_j^{\star} m_{t_i,j}$, where $d_j^{\star} \overset{iid}{\sim} \text{Uniform}(0, 0.5)$,

$$m_{t_i,j} = \begin{cases} m_{1j}^{\star}, & \text{if } 0 \le t_i \le t_1', \\ m_{2j}^{\star}, & \text{if } t_1' < t_i \le t_2', \\ m_{3j}^{\star}, & \text{if } t_2' < t_i \le T, \end{cases}$$

$(m_{1j}^{\star}, m_{2j}^{\star}, m_{3j}^{\star})$ is a random permutation of $\{-1, 0, 1\}$ and (t_1', t_2') is a random sample of size 2 from the set $\{t_2, \ldots, t_{n-1}\}$. Simulated $\tilde{\alpha}_{t,j}^{\text{TR}}$'s are illustrated for some selected OTUs in

Figure 10(b)-(d) with red squares.

The results under the proposed model are illustrated in Figures 9-10. Comparison of the estimates of $\beta_{j,p}$ to their truth shows that the covariate effects are reasonably estimated. Figure 10(a) shows that the assumed convolution process well approximates the irregular patterns in the truth, although the estimates of $g_{t,k,j}$ is slightly deteriorated compared to those under Simulations 1 and 2. Panels (b)-(d) illustrate the estimates of $\tilde{\alpha}_{0,j} + \tilde{\alpha}_{t,j}$ for some selected OTUs, $j = 8$, 34, and 48. The assumed convolution process does not accurately capture the discontinuous patterns in the truth, but still produces reasonable estimates of $\tilde{\alpha}_{0,j} + \tilde{\alpha}_{t,j}$, so reasonable estimates of $g_{t,k,j}$. The estimates of sample specific scale factors $r_{t,k}$ and OTU specific dispersion parameters s_j are compared to their true values in panels (e) and (f), respecitively. Figure 11 illustrates comparisons of the estimates of $\boldsymbol{\beta}$ under the NBMM to the truth. Similar to the results of Simulations 1 and 2, the NBMM yields biased estimates for some covariates with wider interval estimates.

3 Additional Results for Ocean Microbime Data

We demonstrate additional results from the ocean microbime data analysis in §3.2 of the main text. Table 1 has the ranges of the concentration levels to discretize some covariates, the concentration levels of *Alexandrium*, *Dinophysis*, Pseudo-niztschia and domoic acid. Figure 12(a)-(c) illustrates the posterior inference on $\tilde{\alpha}_{0,j} + \tilde{\alpha}_{t,j}$ for selected OTUs, $j = 16$, 34 and 49. Parameters $\tilde{\alpha}_{0,j} + \tilde{\alpha}_{t,j}$ are the baseline expected counts over time adjusted by sample size factors $r_{t,k}$. The dots and blue vertical lines are the posterior means and 95% credible intervals, respectively. Panels (d) and (e) are histograms of the posterior means of sample size factors $\tilde{r}_{t_i,k}$ and overdispersion parameters s_j. The histogram shows left-skeweness of the distribution of $\tilde{r}_{t,k}$. Figures 13 and 14 show histograms of the posterior means $\hat{\beta}_{j,p}$ of $\beta_{j,p}$ under the proposed model and the MLEs $\hat{\beta}^{\text{NBMM}}_{j,p}$ under the NBMM for each covariate,

respectively. The comparison of $\hat{\beta}_{j,p}$ and $\hat{\beta}_{j,p}^{\text{NBMM}}$ for each covariate is shown in Figure 15. For some OTUs, the NBMM yields some greatly larger or smaller estimates than the proposed model. It may be due to convergence problem in the computation algorithm of the NBMM. From the insert plots, the estimates under the proposed model are more shrunken toward zero for more sparse solutions of $\boldsymbol{\beta}$.

References

Park, T. and Casella, G. (2008). The bayesian lasso. *Journal of the American Statistical Association* **103**, 482, 681–686.

Zhang, X., Mallick, H., Tang, Z., Zhang, L., Cui, X., Benson, A. K., and Yi, N. (2017). Negative binomial mixed models for analyzing microbiome count data. *BMC bioinformatics* **18**, 1, 4.

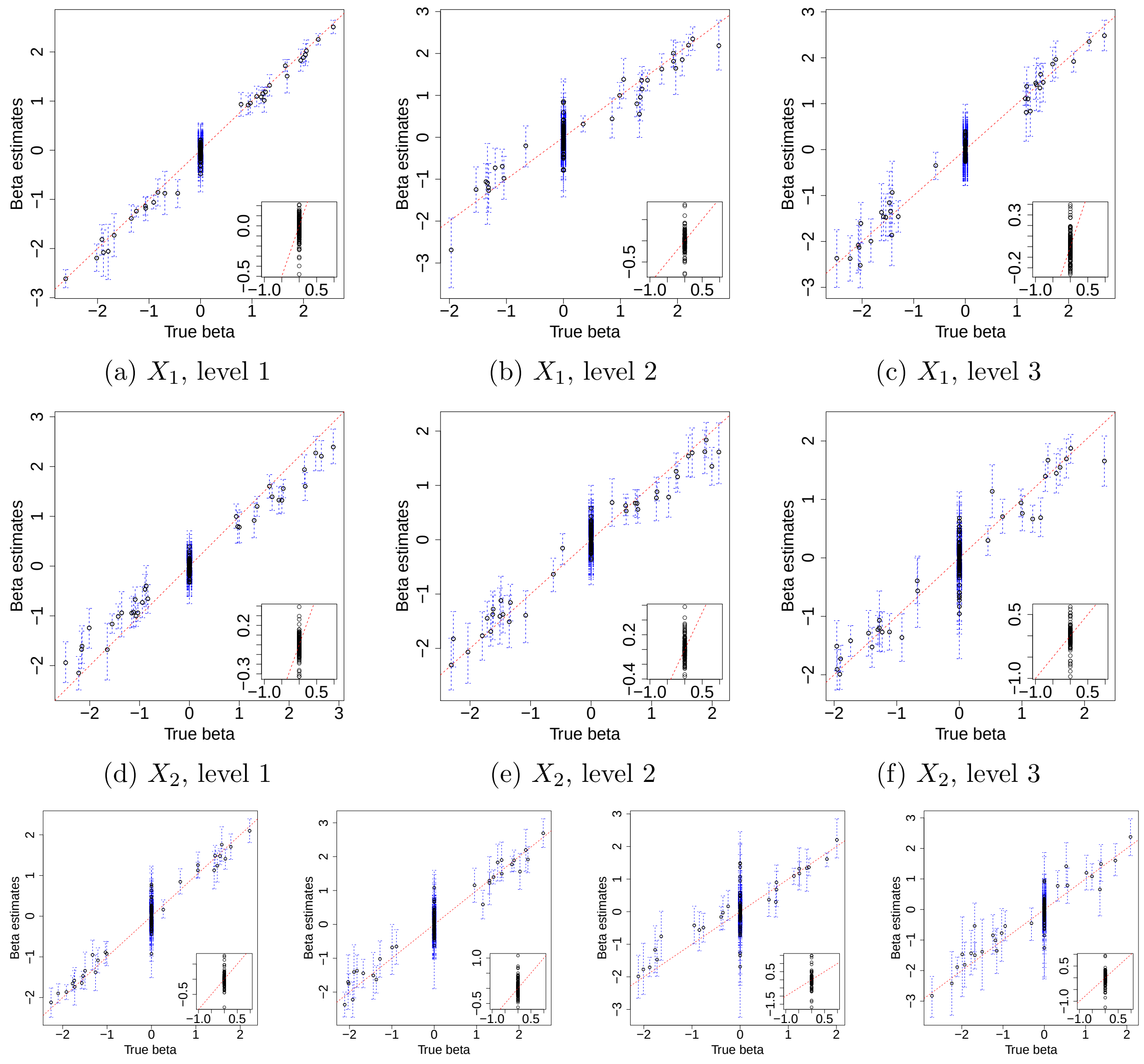

(a) X_1, level 1 (b) X_1, level 2 (c) X_1, level 3

(d) X_2, level 1 (e) X_2, level 2 (f) X_2, level 3

(k) X_3, level 1 (ℓ) X_3, level 2 (m) X_3, level 3 (n) X_3, level 4

Figure 1: [Simulation 1 - Proposed Model] Comparison of the true values $\beta_{j,p}^{\text{TR}}$ and their posterior estimates for each covariate (p). Dots and blue dashed lines represent estimates of posterior means $\hat{\beta}_{j,p}$ of $\beta_{j,p}$ and 95% credible intervals (CIs) of $\beta_{j,p}$, respectively. The insert plot in each panel is a scatter plot of $\hat{\beta}_{j,p}$ and $\beta_{j,p}^{\text{TR}}$ for (j,p) with $\beta_{j,p}^{\text{TR}} = 0$.

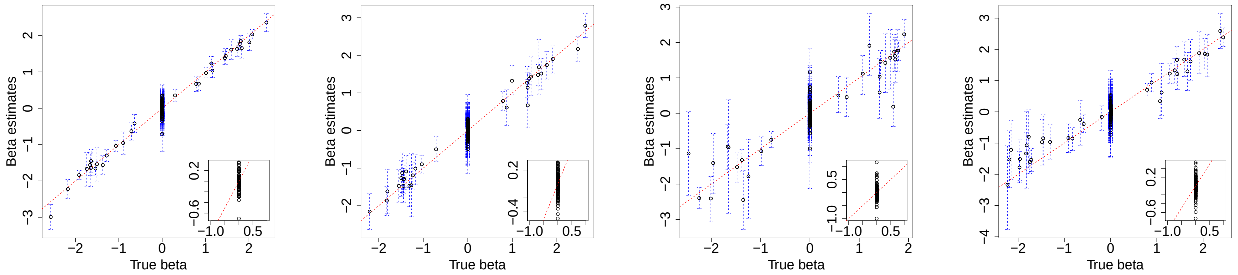

(k) X_4, level 1 (ℓ) X_4, level 2 (m) X_4, level 3 (n) X_4, level 4

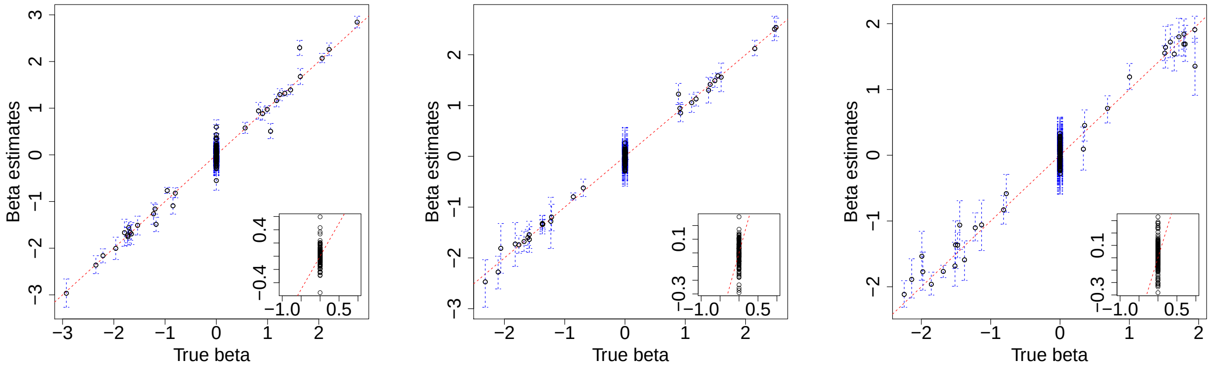

(o) X_5 (p) X_6 (q) X_7

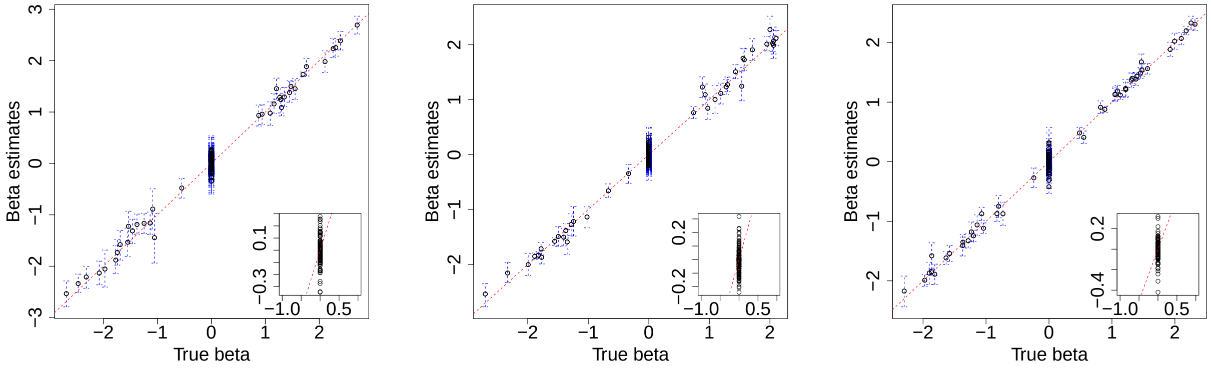

(r) X_8 (s) X_9 (t) X_{10}

Figure 1 (continued) [Simulation 1 - Proposed Model] Comparison of the true values $\beta_{j,p}^{\mathrm{TR}}$ and their posterior estimates for each covariate (p). Dots and blue dashed lines represent estimates of posterior means $\hat{\beta}_{j,p}$ of $\beta_{j,p}$ and 95% credible intervals (CIs) of $\beta_{j,p}$, respectively. The insert plot in each panel is a scatter plot of $\hat{\beta}_{j,p}$ and $\beta_{j,p}^{\mathrm{TR}}$ for (j,p) with $\beta_{j,p}^{\mathrm{TR}} = 0$.

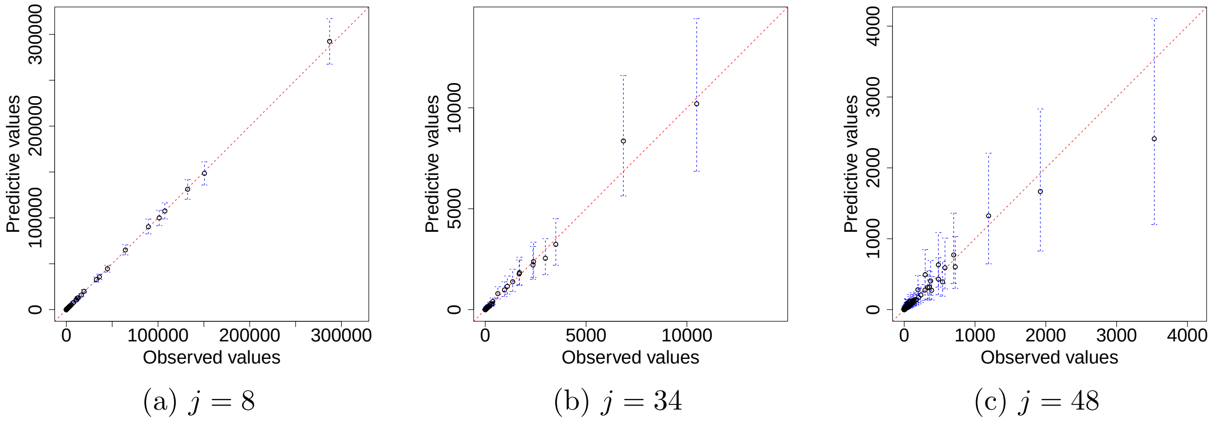

Figure 2: [Simulation 1 - Proposed Model] Scatterplots of observed and predicted values of $Y_{t_i,k,j}$ for OTUs $j = 8$, 34, 48. Blue vertical lines represents 95% posterior predictive intervals of $Y_{t_i,k,j}$.

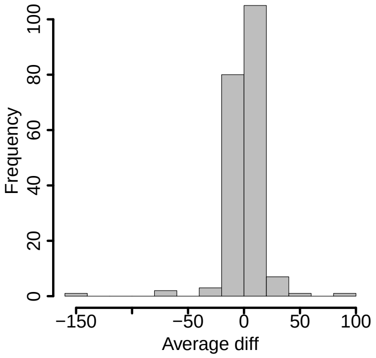

(a) $M = 13$
$(c_r, c_\alpha) = (-7.81, 12.49)$

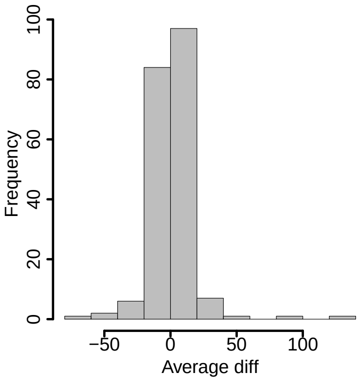

(b) $M = 13$
$(c_r, c_\alpha) = (-7.31, 11, 99)$

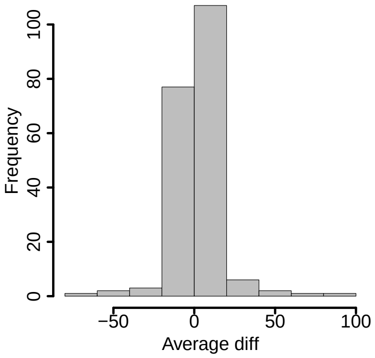

(c) $M = 13$
$(c_r, c_\alpha) = (-8.31, 12.99)$

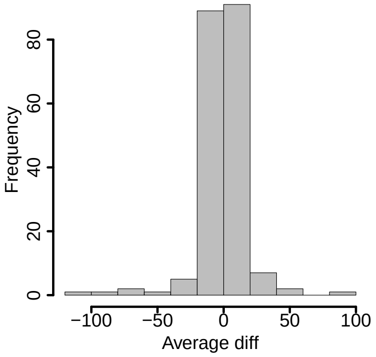

(d) $M = 8$
$(c_r, c_\alpha) = (-7.81, 12.49)$

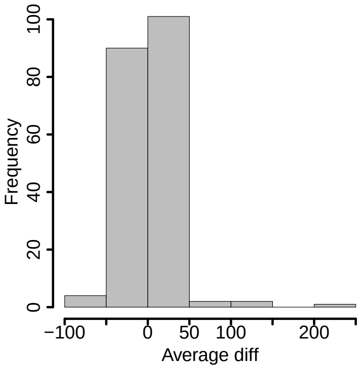

(e) $M = 18$
$(c_r, c_\alpha) = (-7.81, 12.49)$

Figure 3: [Simulation 1 - Proposed Model] Histograms of averaged differences between baseline mean count estimates and their true values, $D_j = \sum_{i=1}^{n} \sum_{k=1}^{K_i} (\hat{g}_{t_i,k,j} - g^{\text{TR}}_{t_i,k,j})/N$, where $\hat{g}_{t,k,j}$ are posterior mean estimates of $g^{\text{TR}}_{t,k,j} = r^{\text{TR}}_{t,k} \exp(\tilde{\alpha}^{\text{TR}}_{0,j} + \tilde{\alpha}^{\text{TR}}_{t,j})$. $M = 13$ and $(c_r, c_\alpha) = (-7.81, 12.49)$ are used for the analysis in the main text. For easy comparison, the histogram of D_j is repeated in (a). Different specifications of the mean constraints $(c_r, c_\alpha) = (-7.31, 11, 99)$ and $(c_r, c_\alpha) = (-8.31, 12.99)$ are used for (b) and (c), respectively, with fixed $M = 13$. The proposed model was fitted with $M = 8$ and 18, while keeping $(c_r, c_\alpha) = (-7.81, 12.49)$ as in (a). The histograms of D_j with $M = 8$ and 18 are illustrated in (d)-(e), respectively

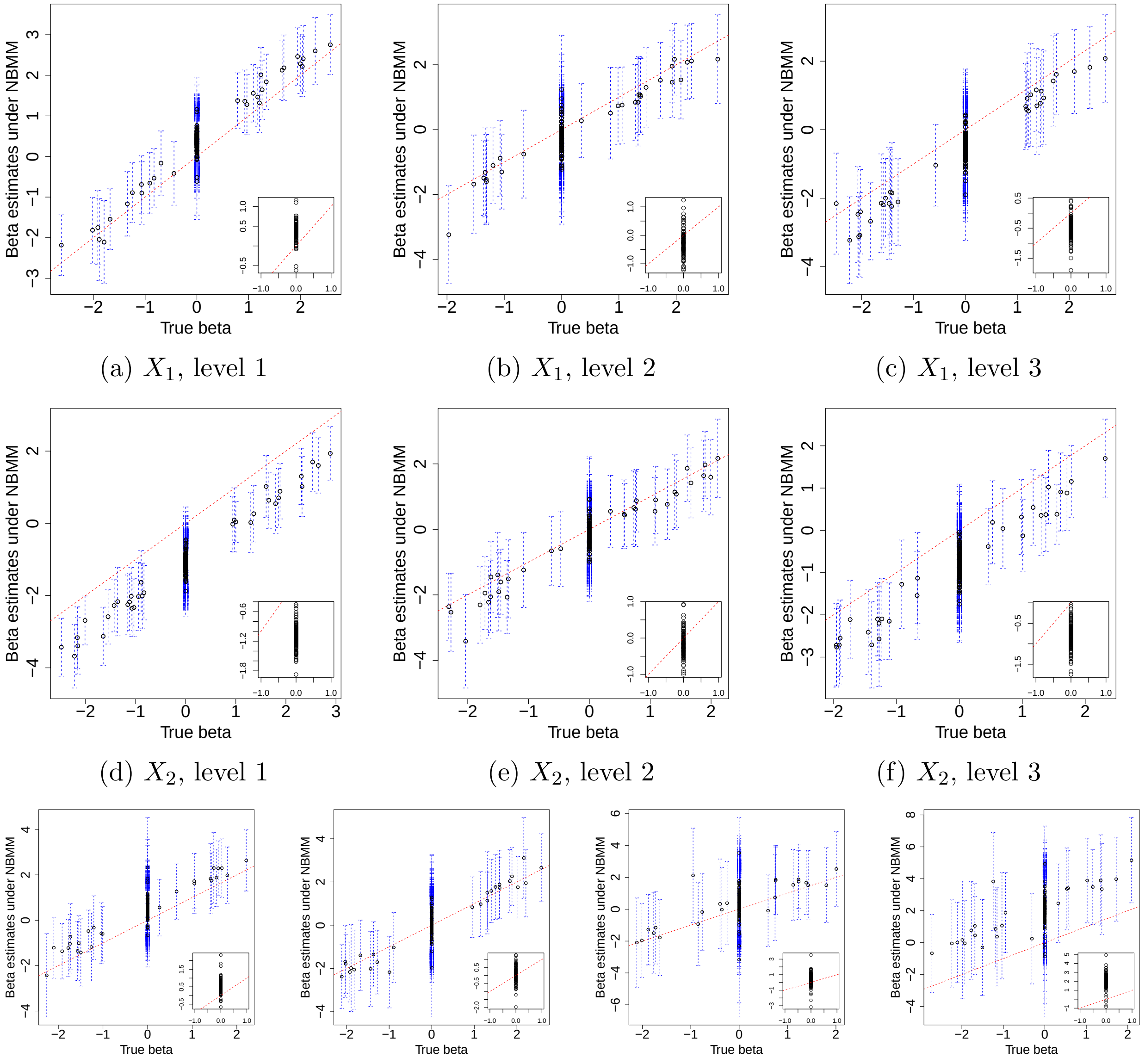

(a) X_1, level 1 (b) X_1, level 2 (c) X_1, level 3

(d) X_2, level 1 (e) X_2, level 2 (f) X_2, level 3

(g) X_3, level 1 (h) X_3, level 2 (i) X_3, level 3 (j) X_3, level 4

Figure 4: [Simulation 1 - NBMM] Comparison of the true values $\beta_{j,p}^{\mathrm{TR}}$ and their maximum likelihood estimates $\hat{\beta}_{j,p}^{\mathrm{NBMM}}$ for each covariate (p) under the negative binomial mixed model (NBMM). Dots and blue dashed lines represent $\hat{\boldsymbol{\beta}}^{\mathrm{NBMM}}$ and their 95% confidence intervals, respectively. The insert plot in each panel is a scatter plot of $\hat{\beta}_{j,p}^{\mathrm{NBMM}}$ and $\beta_{j,p}^{\mathrm{TR}}$ for (j,p) with $\beta_{j,p}^{\mathrm{TR}} = 0$.

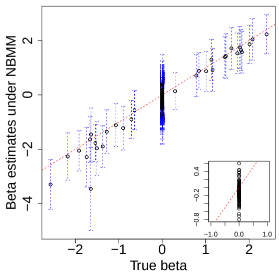

(k) X_4, level 1

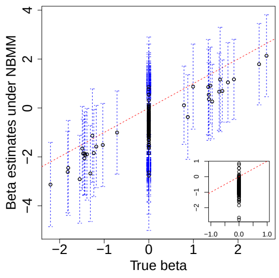

(ℓ) X_4, level 2

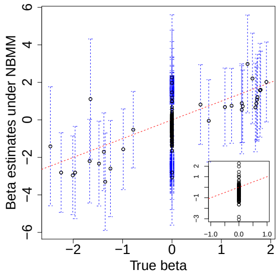

(m) X_4, level 3

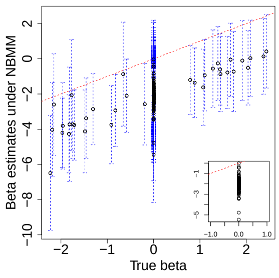

(n) X_4, level 4

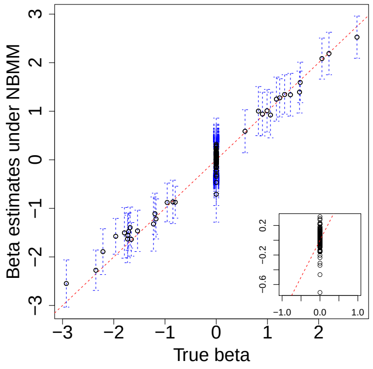

(o) X_5

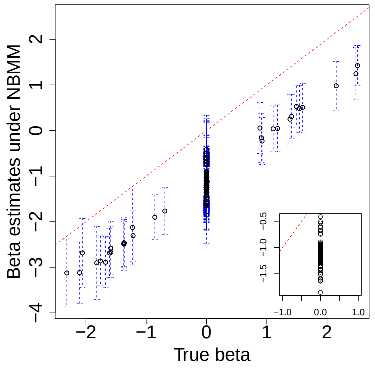

(p) X_6

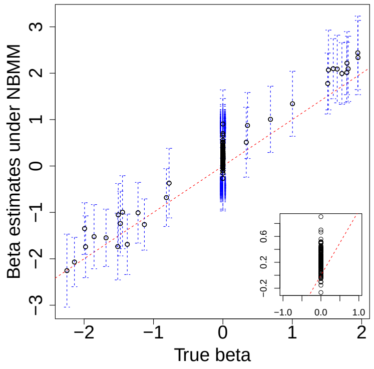

(q) X_7

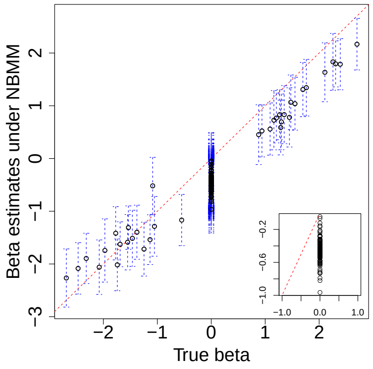

(r) X_8

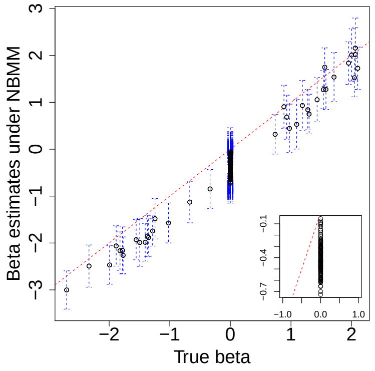

(s) X_9

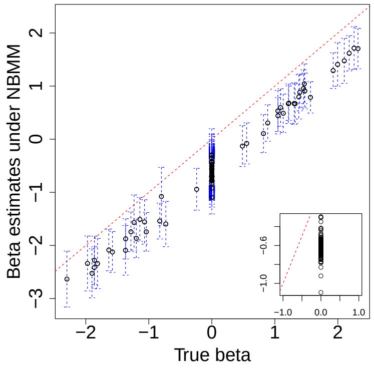

(t) X_{10}

Figure 4 (continued)[Simulation 1 - NBMM] Comparison of the true values $\beta_{j,p}^{\mathrm{TR}}$ and their maximum likelihood estimates $\hat{\beta}_{j,p}^{\mathrm{NBMM}}$ for each covariate (p) under the negative binomial mixed model (NBMM). Dots and blue dashed lines represent $\hat{\boldsymbol{\beta}}^{\mathrm{NBMM}}$ and their 95% confidence intervals, respectively. The insert plot in each panel is a scatter plot of $\hat{\beta}_{j,p}^{\mathrm{NBMM}}$ and $\beta_{j,p}^{\mathrm{TR}}$ for (j,p) with $\beta_{j,p}^{\mathrm{TR}} = 0$.

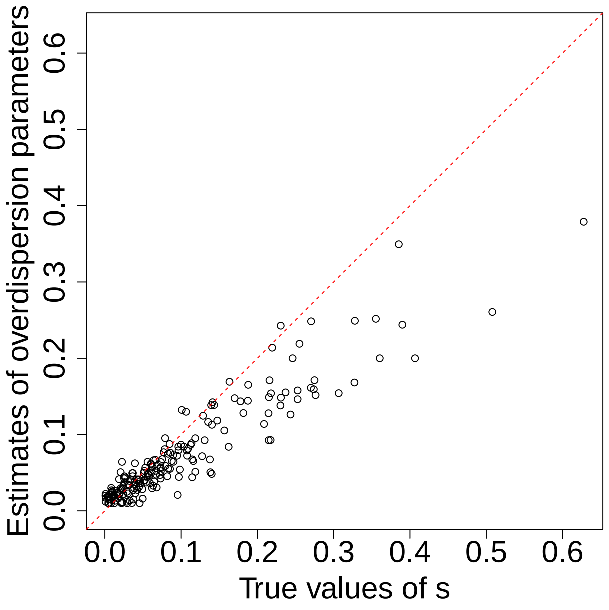

Figure 5: [Simulation 1 - NBMM] Scatterplot of s_j^{TR} vs $1/\hat{\theta}_j^{NBMM}$ where $\hat{\theta}_j^{NBMM}$'s are the estimates of OTU specific dispersion parameters under the negative binomial mixed model (NBMM).

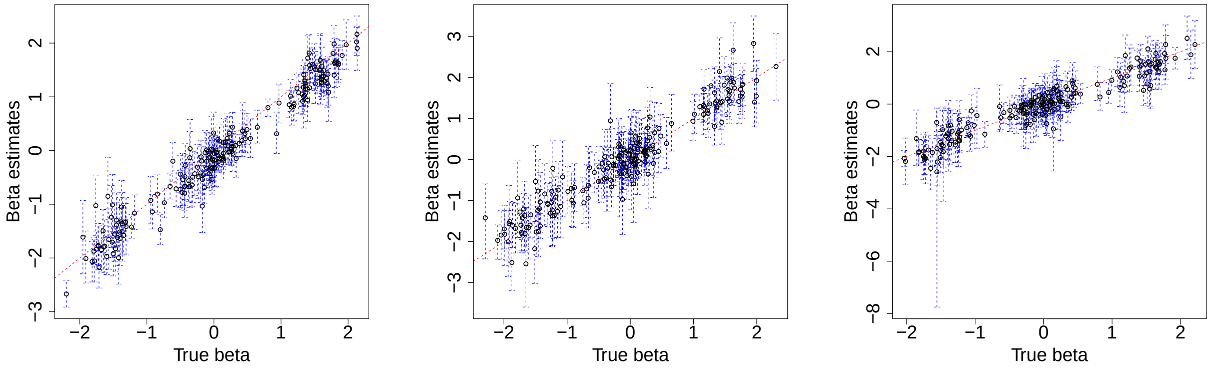

(a) X_1, level 1 (b) X_1, level 2 (c) X_1, level 3

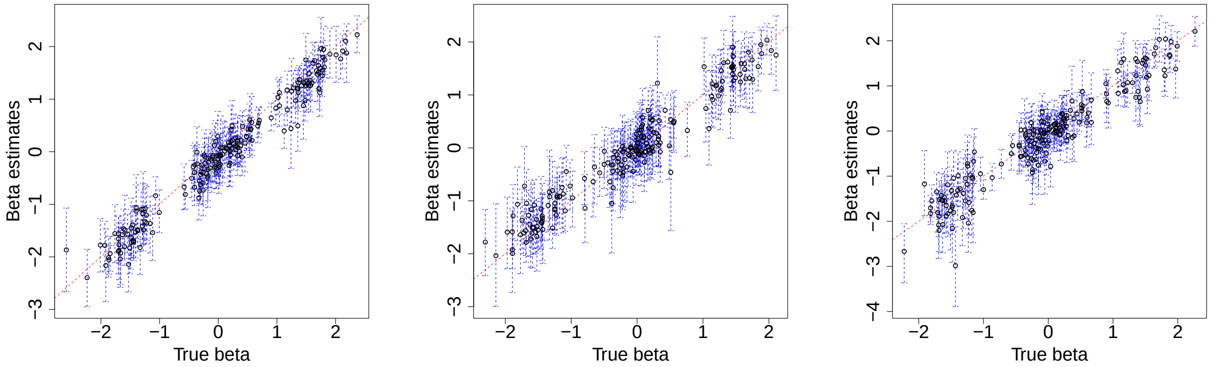

(d) X_2, level 1 (e) X_2, level 2 (f) X_2, level 3

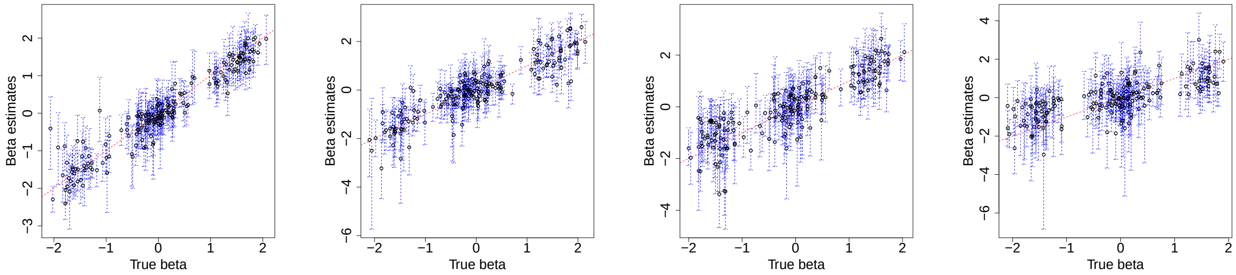

(k) X_3, level 1 (ℓ) X_3, level 2 (m) X_3, level 3 (n) X_3, level 4

Figure 6: [Simulation 2 - Proposed Model] Comparison of the true values $\beta_{j,p}^{\text{TR}}$ and their posterior estimates for each covariate (p). Dots and blue dashed lines represent estimates of posterior means $\hat{\beta}_{j,p}$ of $\beta_{j,p}$ and 95% credible intervals (CIs) of $\beta_{j,p}$, respectively.

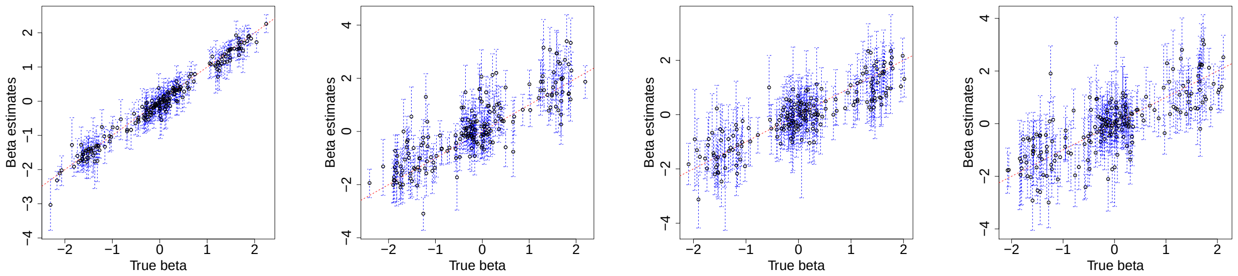

(k) X_4, level 1 (ℓ) X_4, level 2 (m) X_4, level 3 (n) X_4, level 4

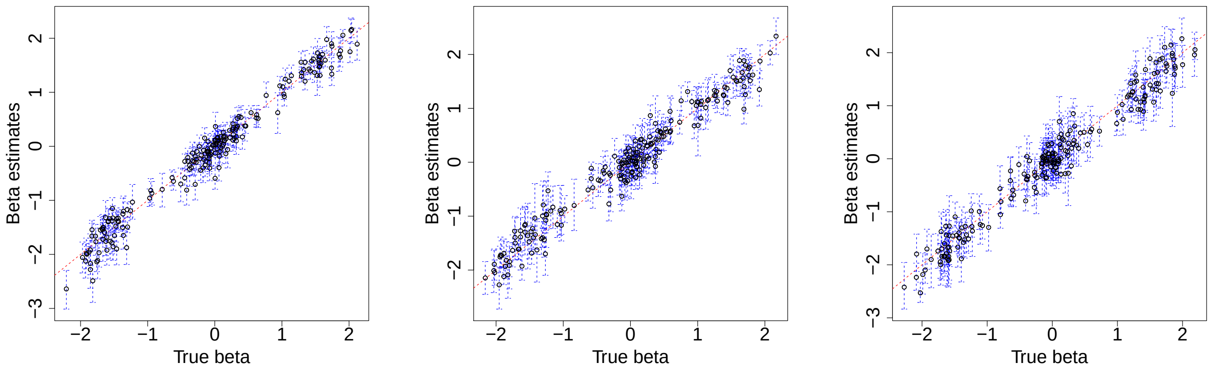

(o) X_5 (p) X_6 (q) X_7

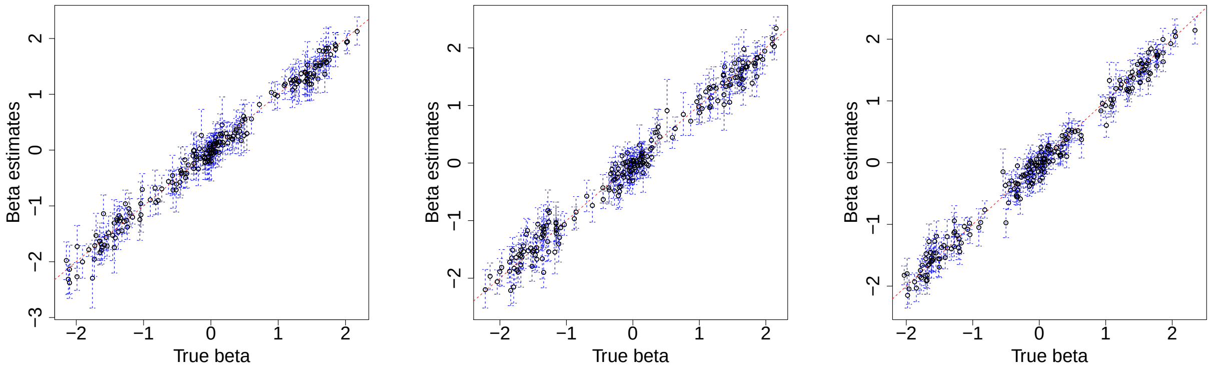

(r) X_8 (s) X_9 (t) X_{10}

Figure 6 (continued) [Simulation 2 - Proposed Model] Comparison of the true values $\beta_{j,p}^{\mathrm{TR}}$ and their posterior estimates for each covariate (p). Dots and blue dashed lines represent estimates of posterior means $\hat{\beta}_{j,p}$ of $\beta_{j,p}$ and 95% credible intervals (CIs) of $\beta_{j,p}$, respectively.

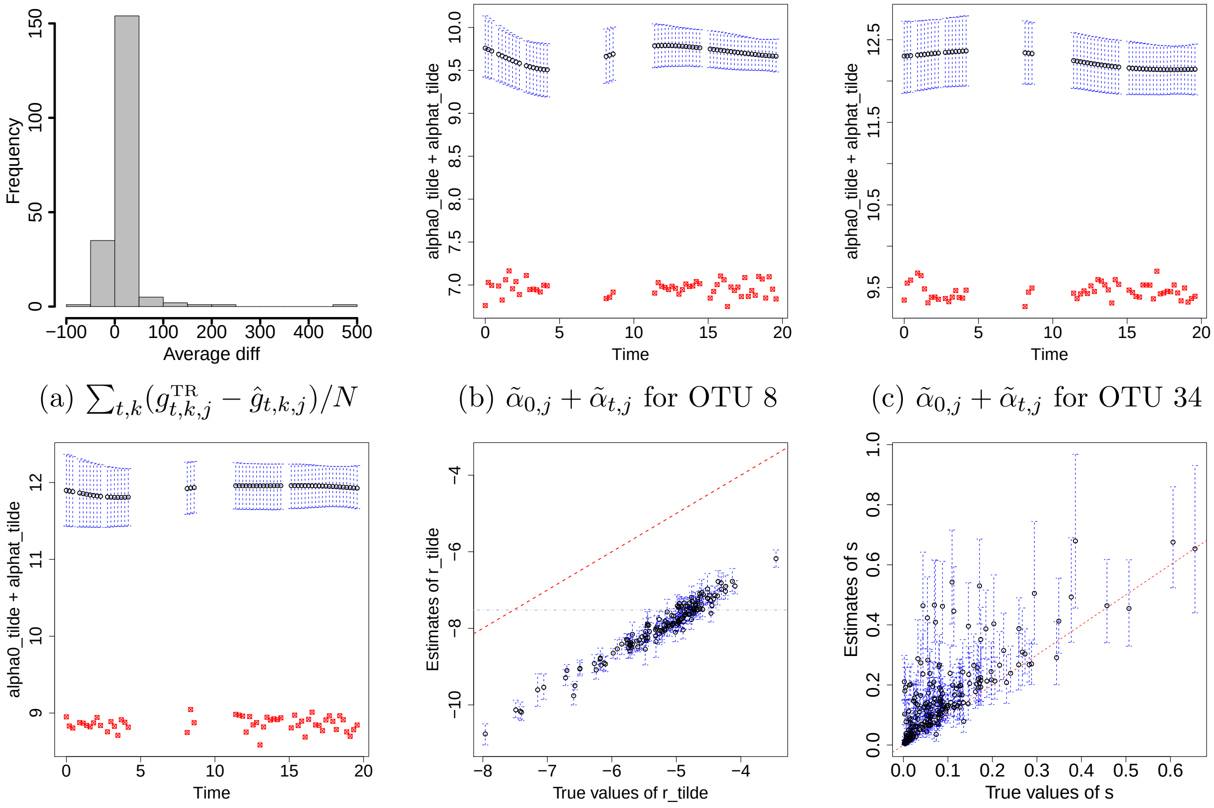

(a) $\sum_{t,k}(g^{\text{TR}}_{t,k,j} - \hat{g}_{t,k,j})/N$ (b) $\tilde{\alpha}_{0,j} + \tilde{\alpha}_{t,j}$ for OTU 8 (c) $\tilde{\alpha}_{0,j} + \tilde{\alpha}_{t,j}$ for OTU 34

(d) $\tilde{\alpha}_{0,j} + \tilde{\alpha}_{t,j}$ for OTU 48 (e) $\tilde{r}^{\text{TR}}$ vs $\hat{\tilde{r}}$ (f) s^{TR}_j vs $\hat{s}_j$

Figure 7: [Simulation 2 - Proposed Model] Panel (a) shows a histogram of averaged difference between $g^{\text{TR}}_{t,k,j}$ and $\hat{g}_{t,k,j}$ for each OTU. Panels (b)-(d) show plots of estimates of $\tilde{\alpha}_{0,j} + \tilde{\alpha}_{t,j}$ over time for some selected OTUs $j = 8, 34, 48$. Panel (e) has a scatterplot of $\hat{\tilde{r}}_{t,k}$ vs $\tilde{r}^{\text{TR}}$. Panel (f) has a scatterplot of $\hat{s}$ vs s^{TR}. Dotes represent posterior mean estimates and blue vertical dotted lines 95% credible intervals. Red squares represent the true values.

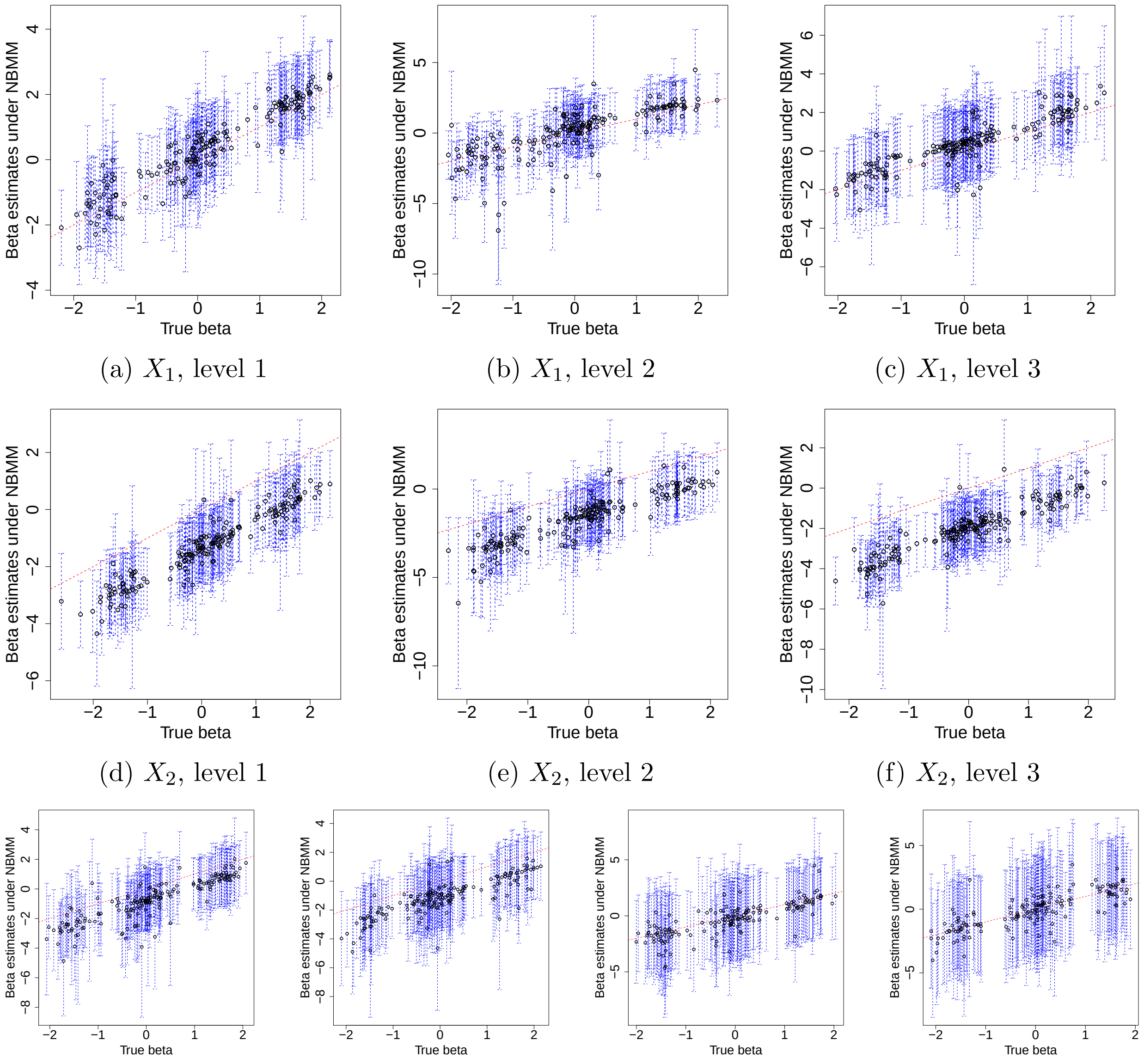

(a) X_1, level 1 (b) X_1, level 2 (c) X_1, level 3

(d) X_2, level 1 (e) X_2, level 2 (f) X_2, level 3

(g) X_3, level 1 (h) X_3, level 2 (i) X_3, level 3 (j) X_3, level 4

Figure 8: [Simulation 2 - NBMM] Comparison of the true values $\beta_{j,p}^{\text{TR}}$ and their maximum likelihood estimates $\hat{\beta}_{j,p}^{\text{NBMM}}$ for each covariate (p) under the negative binomial mixed model (NBMM). Dots and blue dashed lines represent $\hat{\boldsymbol{\beta}}^{\text{NBMM}}$ and their 95% confidence intervals, respectively.

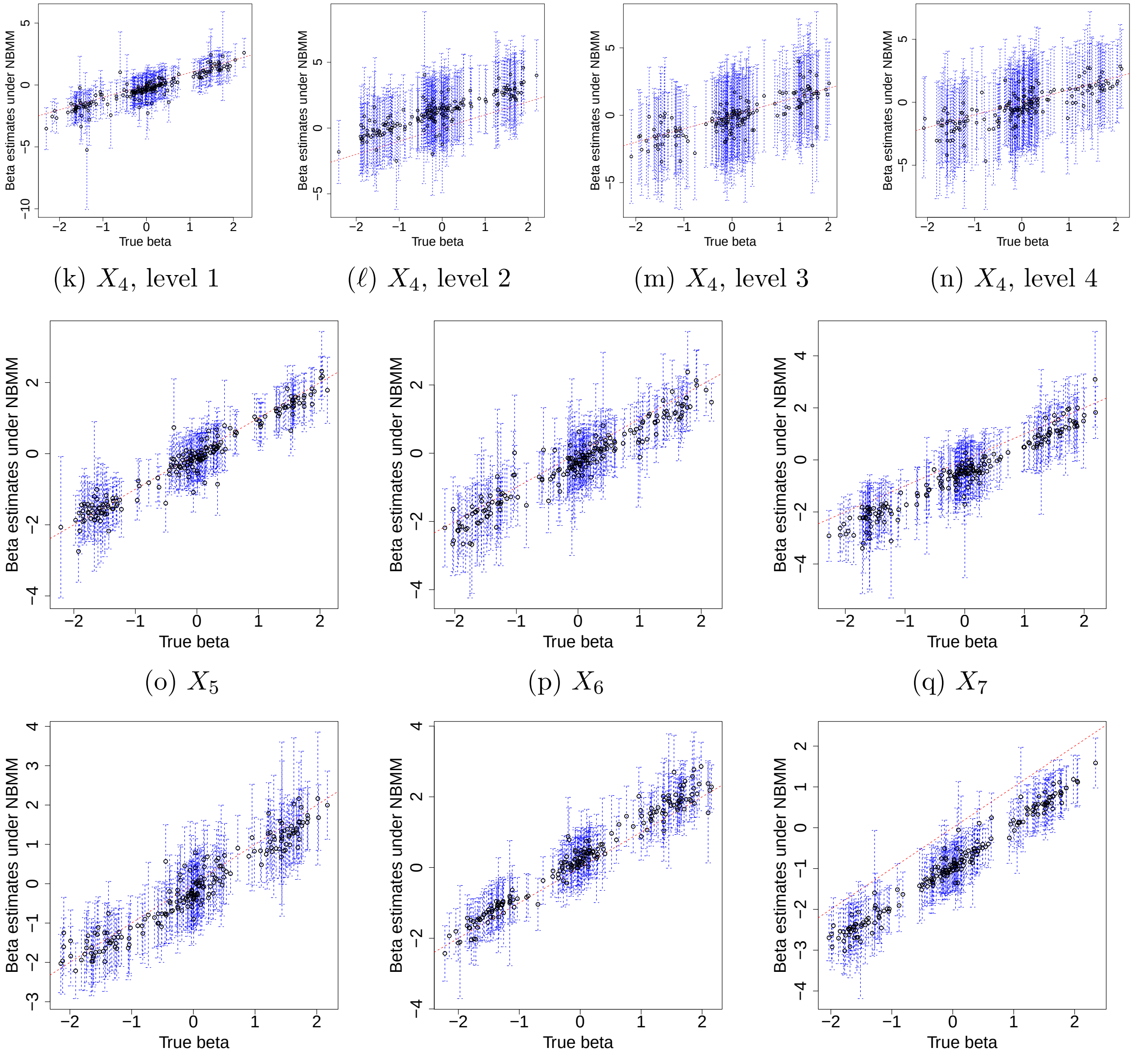

(k) X_4, level 1 (ℓ) X_4, level 2 (m) X_4, level 3 (n) X_4, level 4

(o) X_5 (p) X_6 (q) X_7

(r) X_8 (s) X_9 (t) X_{10}

Figure 8 (continued) [Simulation 2 - NBMM] Comparison of the true values $\beta_{j,p}^{\mathrm{TR}}$ and their maximum likelihood estimates $\hat{\beta}_{j,p}^{\mathrm{NBMM}}$ for each covariate (p) under the negative binomial mixed model (NBMM). Dots and blue dashed lines represent $\hat{\boldsymbol{\beta}}^{\mathrm{NBMM}}$ and their 95% confidence intervals, respectively.

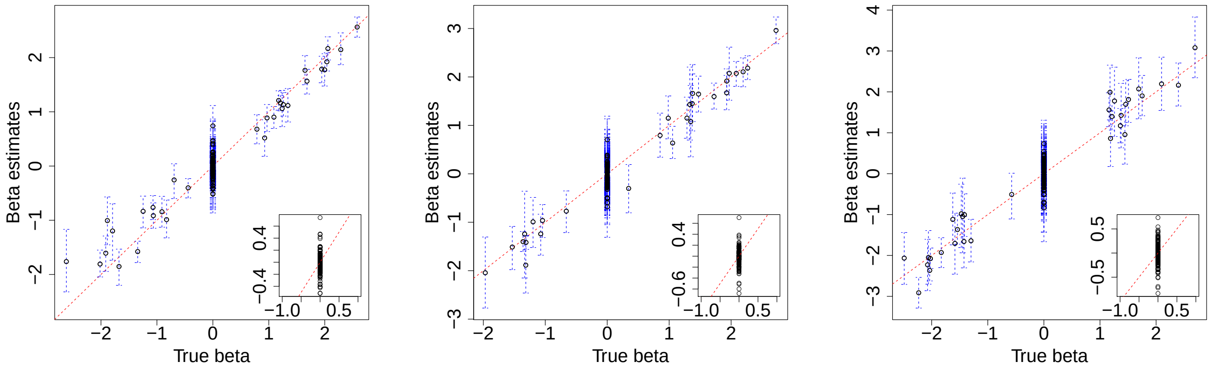

(a) X_1, level 1 (b) X_1, level 2 (c) X_1, level 3

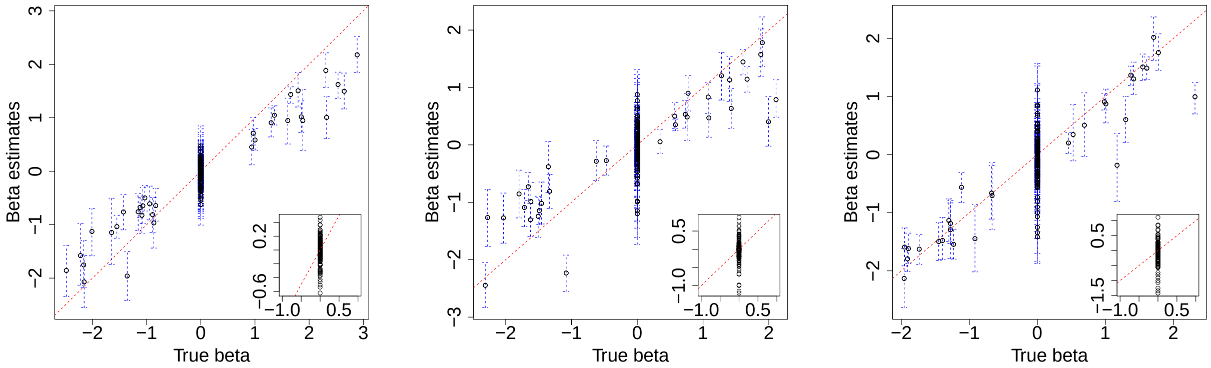

(d) X_2, level 1 (e) X_2, level 2 (f) X_2, level 3

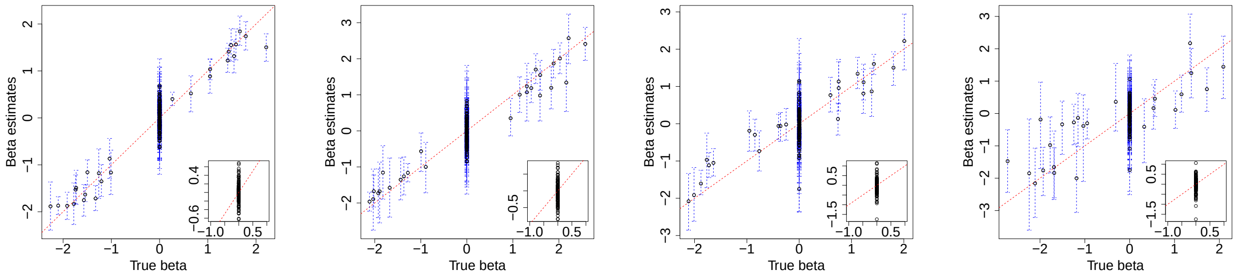

(k) X_3, level 1 (ℓ) X_3, level 2 (m) X_3, level 3 (n) X_3, level 4

Figure 9: [Simulation 3 - Proposed Model] Comparison of the true values $\beta_{j,p}^{\text{TR}}$ and their posterior estimates for each covariate (p). Dots and blue dashed lines represent estimates of posterior means $\hat{\beta}_{j,p}$ of $\beta_{j,p}$ and 95% credible intervals (CIs) of $\beta_{j,p}$, respectively. The insert plot in each panel is a scatter plot of $\hat{\beta}_{j,p}$ and $\beta_{j,p}^{\text{TR}}$ for (j,p) with $\beta_{j,p}^{\text{TR}} = 0$.

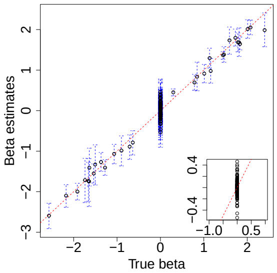

(k) X_4, level 1

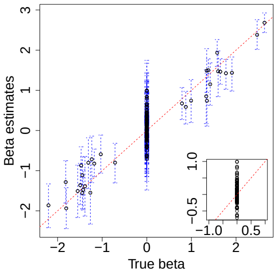

(ℓ) X_4, level 2

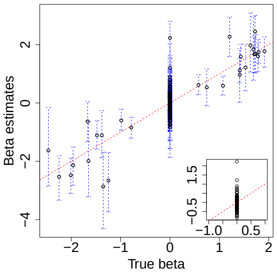

(m) X_4, level 3

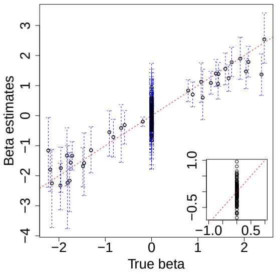

(n) X_4, level 4

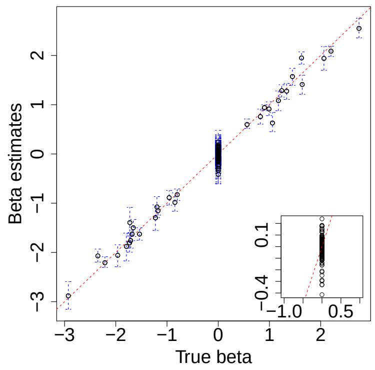

(o) X_5

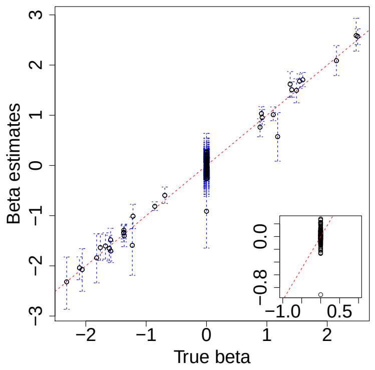

(p) X_6

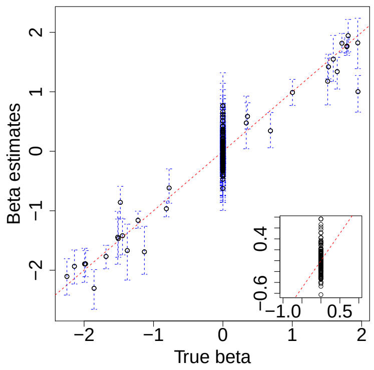

(q) X_7

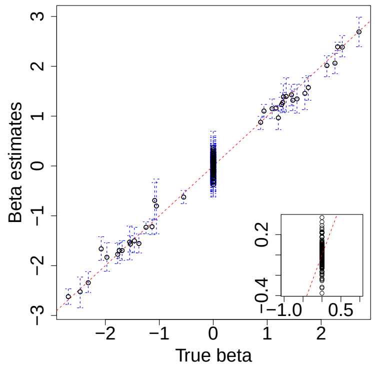

(r) X_8

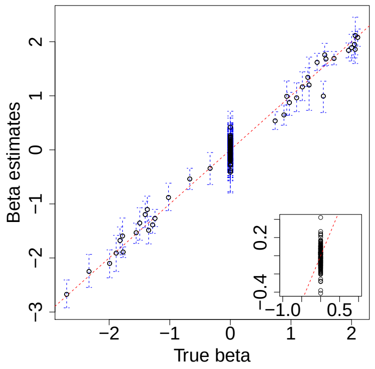

(s) X_9

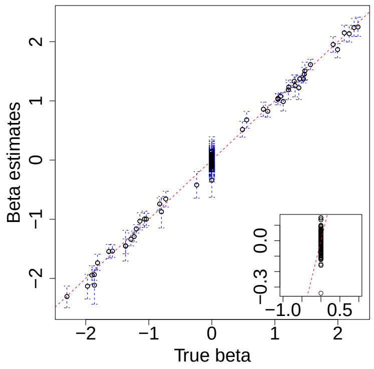

(t) X_{10}

Figure 9 (continued) [Simulation 3 - Proposed Model] Comparison of the true values $\beta_{j,p}^{\mathrm{TR}}$ and their posterior estimates for each covariate (p). Dots and blue dashed lines represent estimates of posterior means $\hat{\beta}_{j,p}$ of $\beta_{j,p}$ and 95% credible intervals (CIs) of $\beta_{j,p}$, respectively. The insert plot in each panel is a scatter plot of $\hat{\beta}_{j,p}$ and $\beta_{j,p}^{\mathrm{TR}}$ for (j,p) with $\beta_{j,p}^{\mathrm{TR}} = 0$.

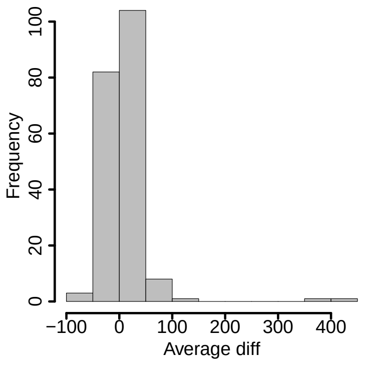

(a) $\sum_{t,k}(g^{\mathrm{TR}}_{t,k,j} - \hat{g}_{t,k,j})/N$

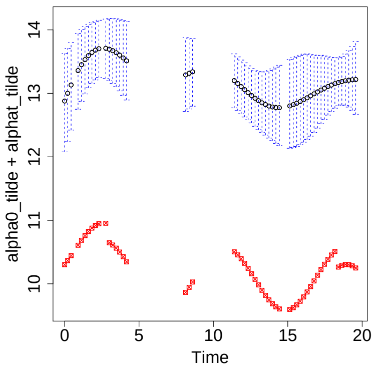

(b) $\tilde{\alpha}_{0,j} + \tilde{\alpha}_{t,j}$ for OTU 8

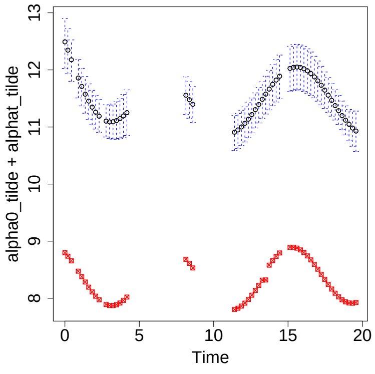

(c) $\tilde{\alpha}_{0,j} + \tilde{\alpha}_{t,j}$ for OTU 34

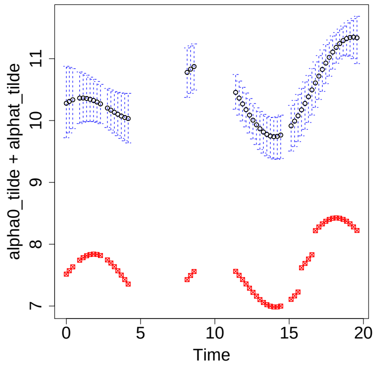

(d) $\tilde{\alpha}_{0,j} + \tilde{\alpha}_{t,j}$ for OTU 48

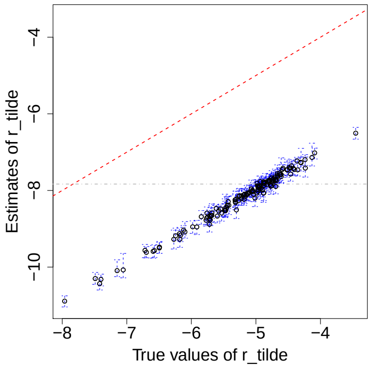

(e) $\tilde{r}^{\mathrm{TR}}$ vs $\hat{\tilde{r}}$

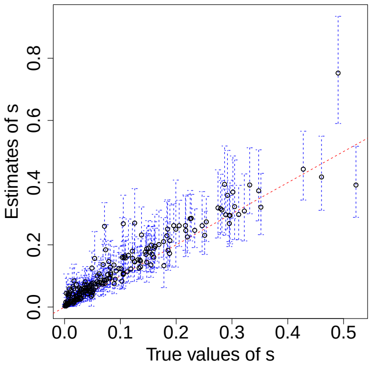

(f) s^{TR}_j vs $\hat{s}_j$

Figure 10: [Simulation 3 - Proposed Model] Panel (a) shows a histogram of averaged difference between $g^{\mathrm{TR}}_{t,k,j}$ and $\hat{g}_{t,k,j}$ for each OTU. Panels (b)-(d) show plots of estimates of $\tilde{\alpha}_{0,j}+\tilde{\alpha}_{t,j}$ over time for some selected OTUs $j = 8, 34, 48$. Panel (e) has a scatterplot of $\hat{\tilde{r}}_{t,k}$ vs $\tilde{r}^{\mathrm{TR}}$. Panel (f) has a scatterplot of $\hat{s}$ vs s^{TR}. Dotes represent posterior mean estimates and blue vertical dotted lines 95% credible intervals. Red squares represent the true values.

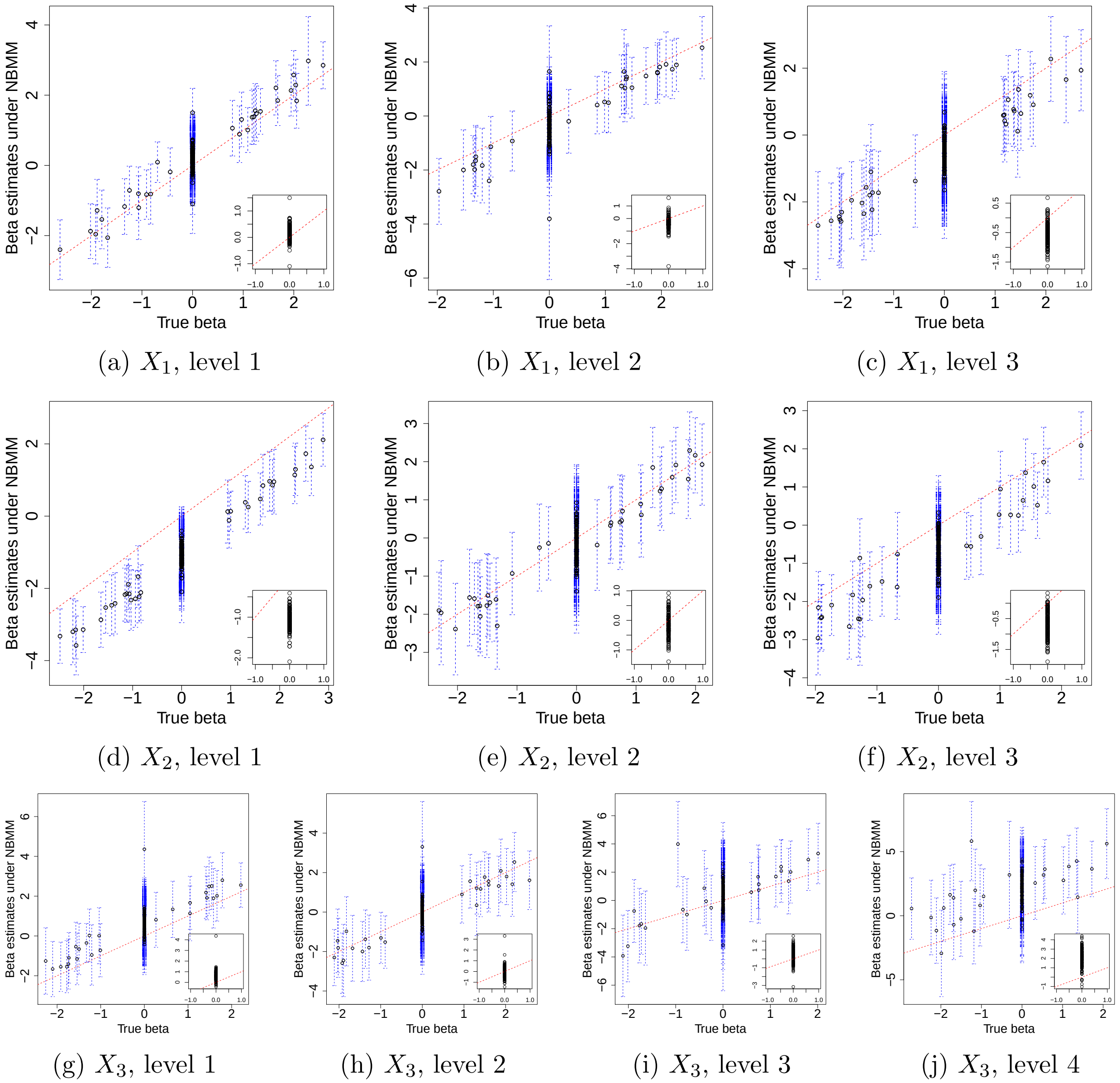

(a) X_1, level 1 (b) X_1, level 2 (c) X_1, level 3

(d) X_2, level 1 (e) X_2, level 2 (f) X_2, level 3

(g) X_3, level 1 (h) X_3, level 2 (i) X_3, level 3 (j) X_3, level 4

Figure 11: [Simulation 3 - NBMM] Comparison of the true values $\beta_{j,p}^{\mathrm{TR}}$ and their maximum likelihood estimates $\hat{\beta}_{j,p}^{\mathrm{NBMM}}$ for each covariate (p) under the negative binomial mixed model (NBMM). Dots and blue dashed lines represent $\hat{\boldsymbol{\beta}}^{\mathrm{NBMM}}$ and their 95% confidence intervals, respectively. The insert plot in each panel is a scatter plot of $\hat{\beta}_{j,p}^{\mathrm{NBMM}}$ and $\beta_{j,p}^{\mathrm{TR}}$ for (j,p) with $\beta_{j,p}^{\mathrm{TR}} = 0$.

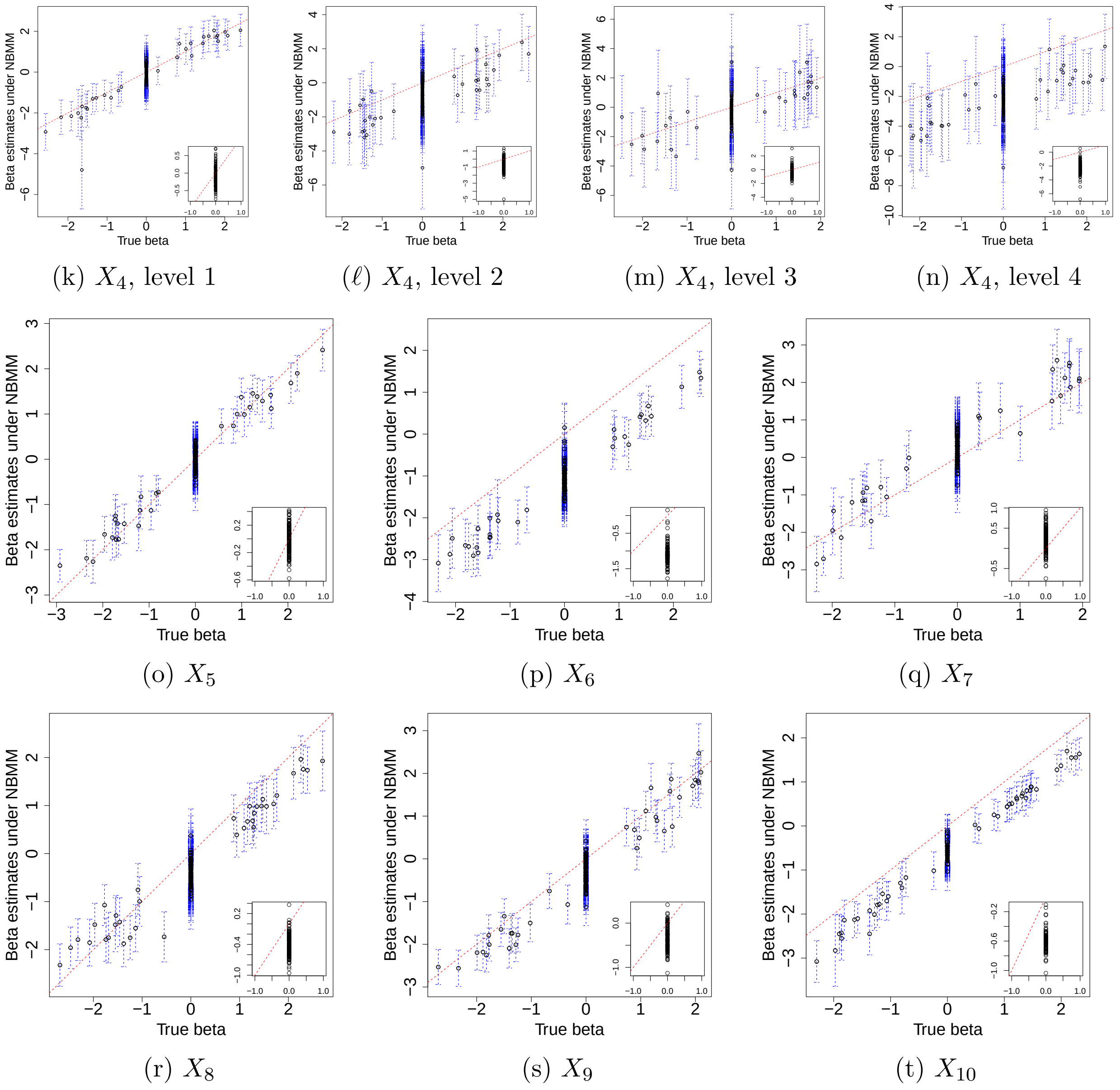

Figure 11 (continued)[Simulation 3 - NBMM] Comparison of the true values of $\beta_{j,p}$ $\beta_{j,p}^{\mathrm{TR}}$ and their maximum likelihood estimates $\hat{\beta}_{j,p}^{\mathrm{NBMM}}$ for each covariate (p) under the negative binomial mixed model (NBMM). Dots and blue dashed lines represent $\hat{\boldsymbol{\beta}}^{\mathrm{NBMM}}$ and their 95% confidence intervals, respectively. The insert plot in each panel is a scatter plot of $\hat{\beta}_{j,p}^{\mathrm{NBMM}}$ and $\beta_{j,p}^{\mathrm{TR}}$ for (j, p) with $\beta_{j,p}^{\mathrm{TR}} = 0$.

Categories	*Alexandrium*& *Dinophysis* (cell counts/L)	*Pseudo-nitzschia* (cell counts/L)	DA concentration (ng/ml)
None (0)	0 to 99	0 to 10000	< 0.01
Low (1)	100 to 500	1001 to 30000	$0.01 \leq X < 0.5$
Medium (2)	501 to 1000	30001 to 100000	$0.5 \leq X < 1.0$
High (3)	1001 to 10000	100001 to 300000	$1.0 \leq X < 5.0$
Very High (4)	$10001 \leq$	$300001 \leq$	$5.0 \leq X$

Table 1: [Ocean Microbiome Data] Ranges used to discretize the concentration levels of *Alexandrium* (Ax, X_1), *Dinophysis* (Dp, X_2), Pseudo-nitzchia (Pn, X_3), and domoic acid (DA, X_4).

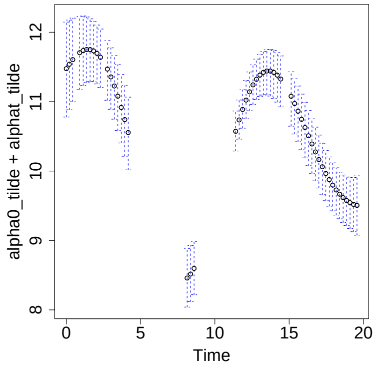

(a) $\tilde{\alpha}_{0,j} + \tilde{\alpha}_{t,j}$ for OTU 16

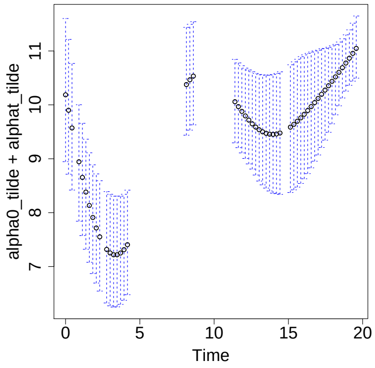

(b) $\tilde{\alpha}_{0,j} + \tilde{\alpha}_{t,j}$ for OTU 34

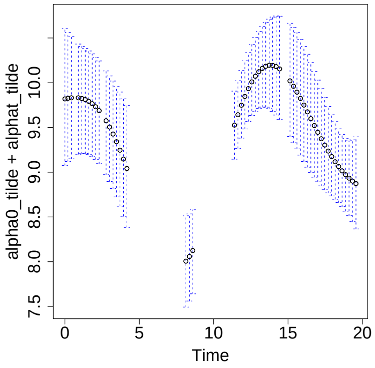

(c) $\tilde{\alpha}_{0,j} + \tilde{\alpha}_{t,j}$ for OTU 49

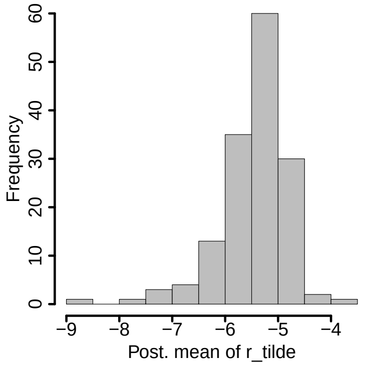

(d) $\hat{\tilde{r}}_{t_i,k}$

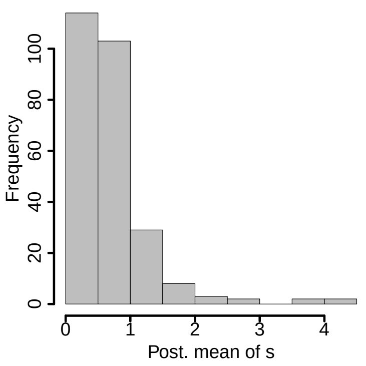

(e) $\hat{s}_j$

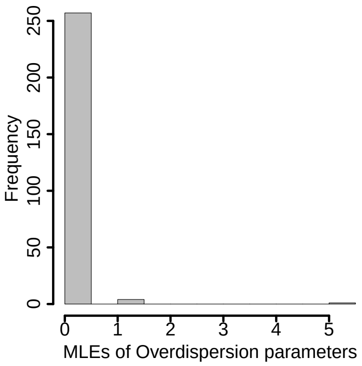

(f) $1/\hat{\theta}_j^{\text{NBMM}}$

Figure 12: [Ocean Microbiome Data - Proposed Model] Posterior inference on baseline expected counts normalized by the sample size factor $\tilde{\alpha}_{0,j} + \tilde{\alpha}_{t,j}$ over time are illustrated for OTUs $j = 16, 21, 34$ in panels (a)-(c), respectively. Dotes represent posterior mean estimates and blue vertical dotted lines 95% credible intervals. A histogram of the posterior mean estimates $\hat{r}_{t_i,k}$ of $r_{t_i,k}$ is shown in panel (d). Histograms of estimates of overdispersion parameters are illustrated in panel (e) and (f). The posterior means $\hat{s}_j$ of s_j under the proposed model and the MLEs $1/\hat{\theta}_j^{NBMM}$ under the negative binomial mixed model (NBMM) are used in panels (e) and (f), respectively.

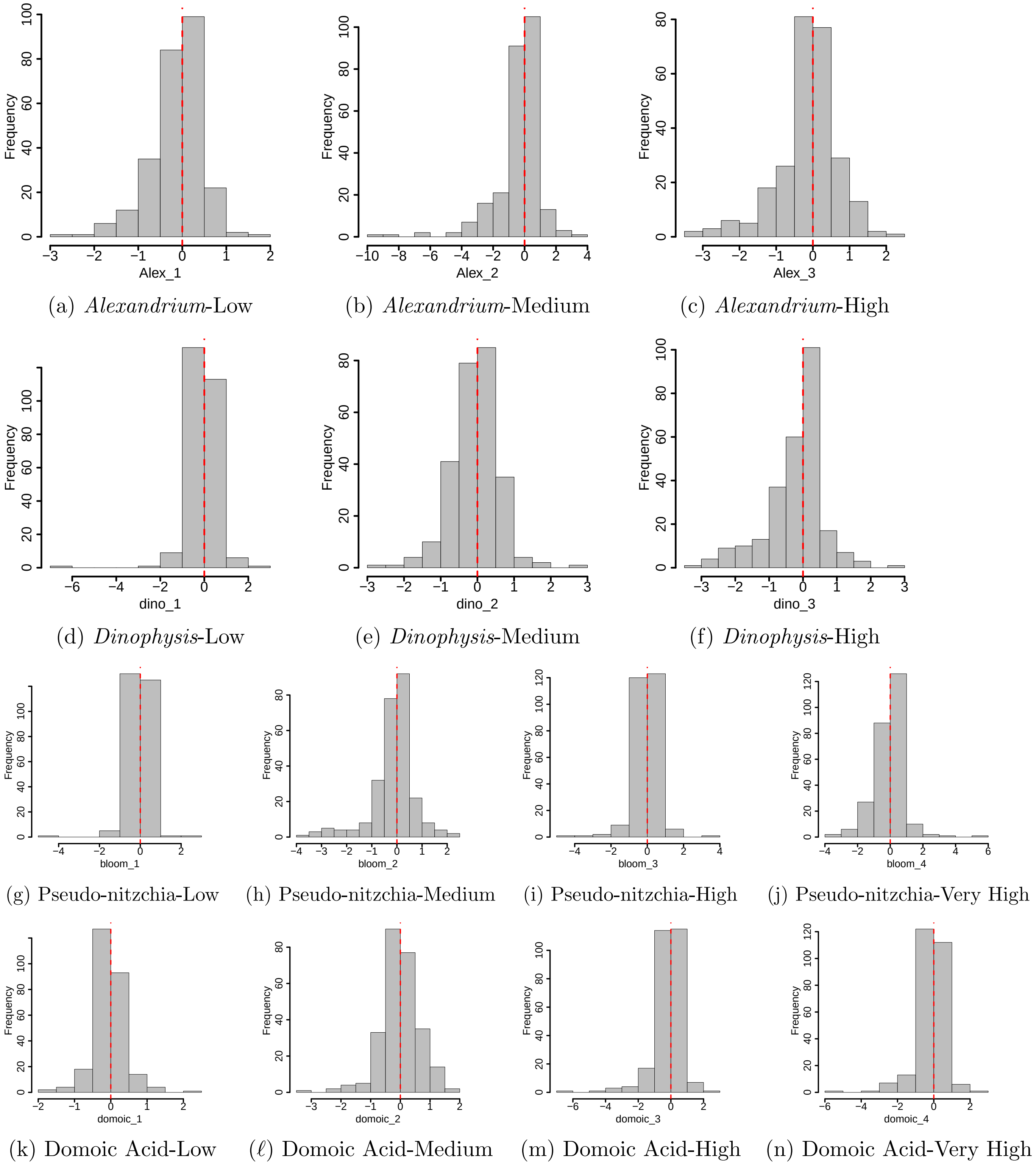

(a) *Alexandrium*-Low (b) *Alexandrium*-Medium (c) *Alexandrium*-High

(d) *Dinophysis*-Low (e) *Dinophysis*-Medium (f) *Dinophysis*-High

(g) Pseudo-nitzchia-Low (h) Pseudo-nitzchia-Medium (i) Pseudo-nitzchia-High (j) Pseudo-nitzchia-Very High

(k) Domoic Acid-Low (ℓ) Domoic Acid-Medium (m) Domoic Acid-High (n) Domoic Acid-Very High

Figure 13: [Ocean Microbiome Data - Proposed Model] Histograms of the posterior means of $\beta_{j,p}$ for each covariate (p).

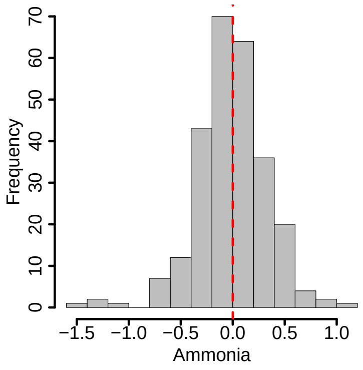

(o) Ammonia

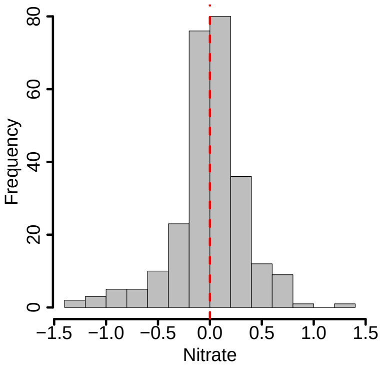

(p) Nitrate

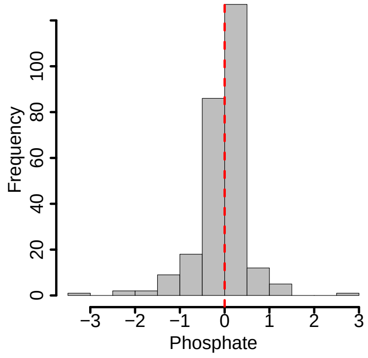

(q) Phosphate

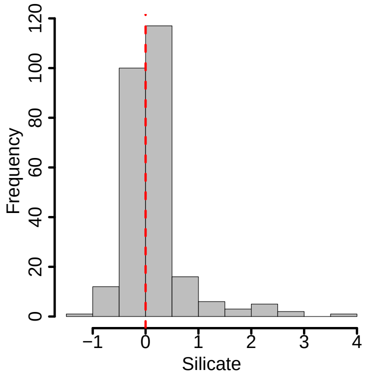

(r) Silicate

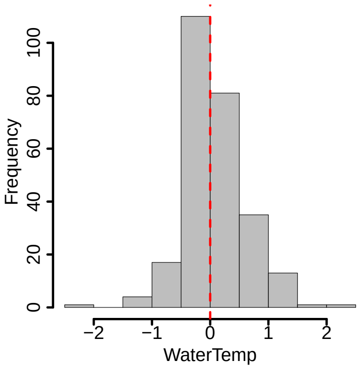

(s) Water temperature

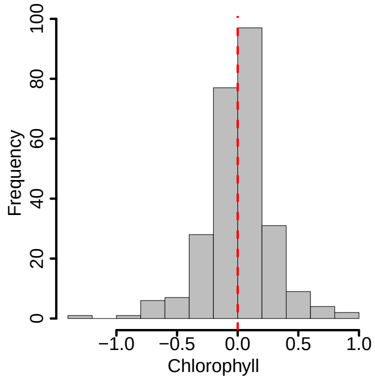

(t) Chlorophyll

Figure 13 (continued) [Ocean Microbiome Data - Proposed Model] Histograms of the posterior means of $\beta_{j,p}$ for each covariate (p).

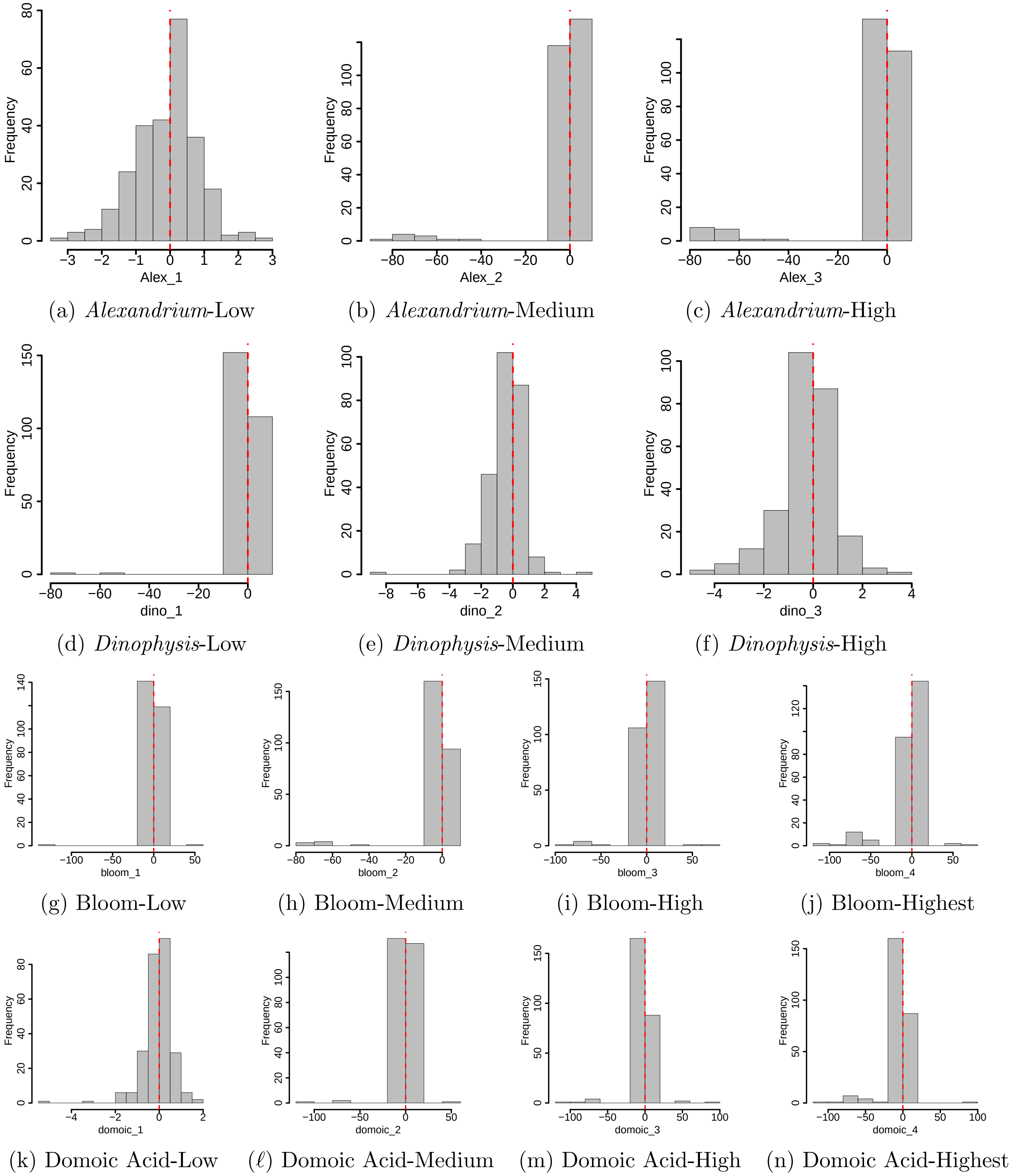

(a) *Alexandrium*-Low (b) *Alexandrium*-Medium (c) *Alexandrium*-High

(d) *Dinophysis*-Low (e) *Dinophysis*-Medium (f) *Dinophysis*-High

(g) Bloom-Low (h) Bloom-Medium (i) Bloom-High (j) Bloom-Highest

(k) Domoic Acid-Low (ℓ) Domoic Acid-Medium (m) Domoic Acid-High (n) Domoic Acid-Highest

Figure 14: [Ocean Microbiome Data - NBMM] Histograms of the maximum likelihood estimates $\hat{\beta}_{j,p}^{\mathrm{NBMM}}$ of $\beta_{j,p}$ under the negative binomial mixed model (NBMM) for each covariate (p).

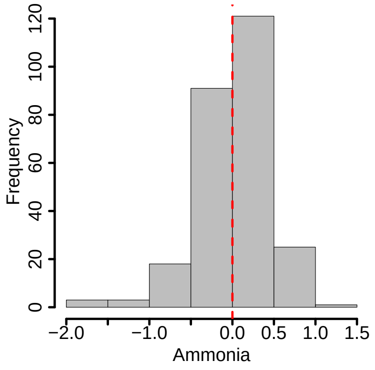

(o) Ammonia

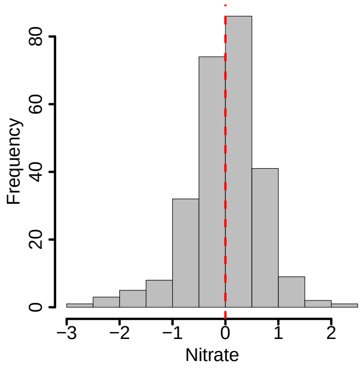

(p) Nitrate

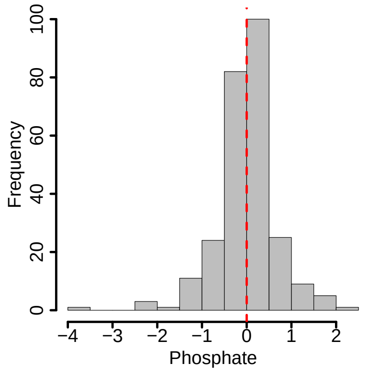

(q) Phosphate

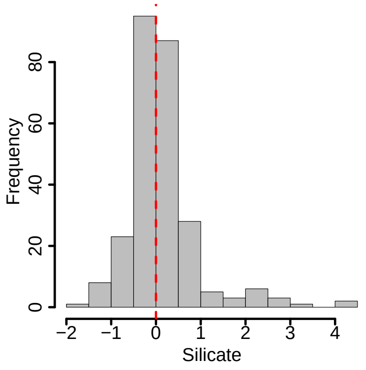

(r) Silicate

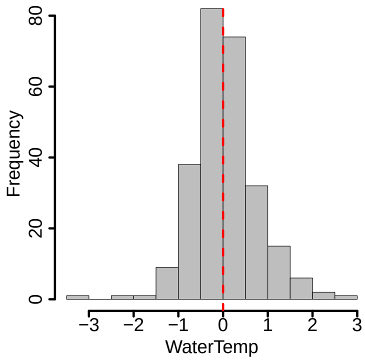

(s) Water temperature

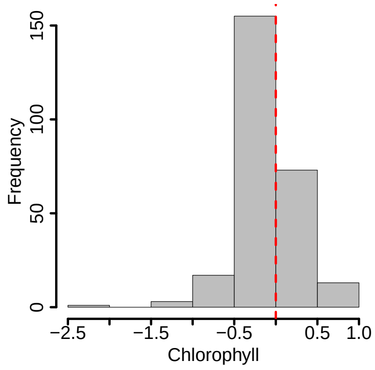

(t) Chlorophyll

Table 14 (continued) [Ocean Microbiome Data - NBMM] the maximum likelihood estimates $\hat{\beta}_{j,p}^{\text{NBMM}}$ of $\beta_{j,p}$ under the negative binomial mixed model (NBMM) for each covariate (p).

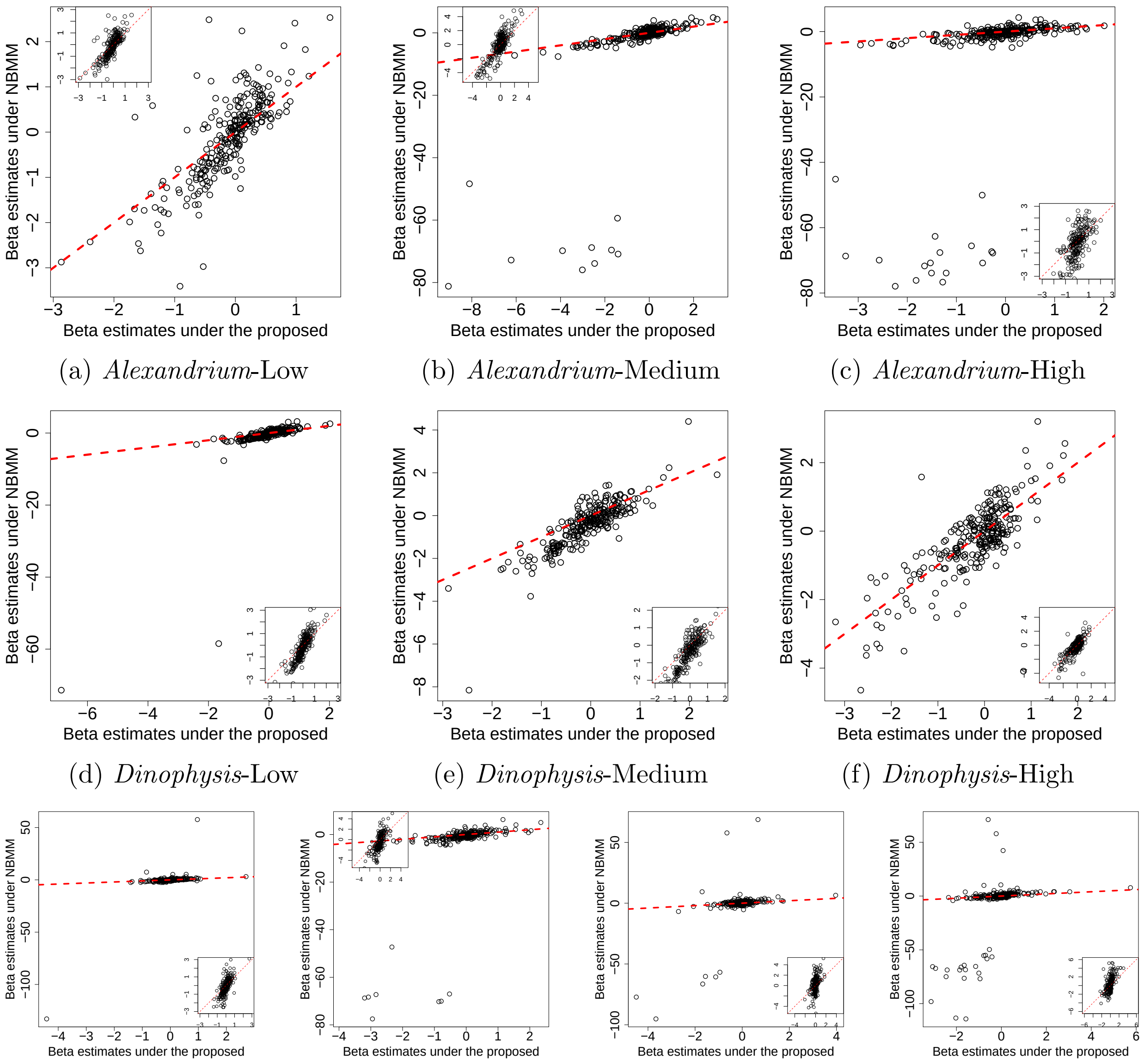

(a) *Alexandrium*-Low (b) *Alexandrium*-Medium (c) *Alexandrium*-High

(d) *Dinophysis*-Low (e) *Dinophysis*-Medium (f) *Dinophysis*-High

(g) Bloom-Low (h) Bloom-Medium (i) Bloom-High (j) Bloom-Highest

Figure 15: [Ocean Microbiome Data - Proposed Model vs NBMM] Comparison of the posterior mean estimates $\hat{\beta}_{j,p}$ under the proposed model and maximum likelihood estimates $\hat{\beta}^{\text{NBMM}}_{j,p}$ under the negative binomial mixed model (NBMM) for each covariate (p). The insert plot in each panel is a scatter plot for regions with small values of estimates.

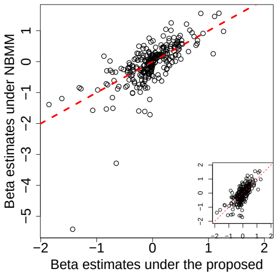

(k) Domoic Acid-Low

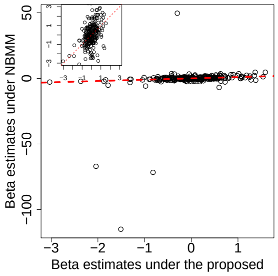

(ℓ) Domoic Acid-Medium

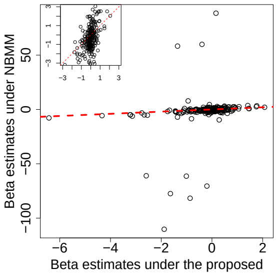

(m) Domoic Acid-High

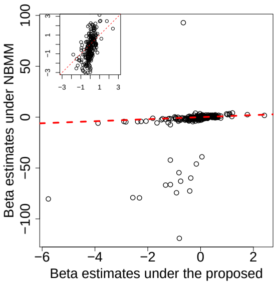

(n) Domoic Acid-Highest

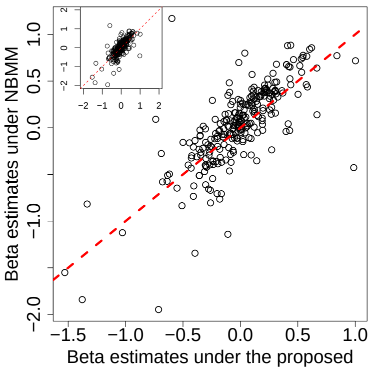

(o) Ammonia

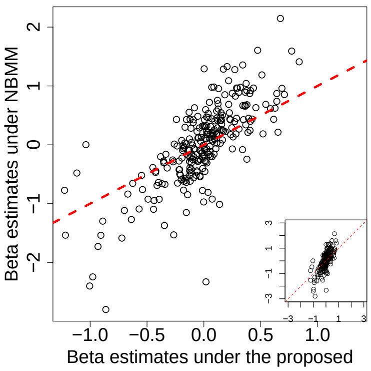

(p) Nitrate

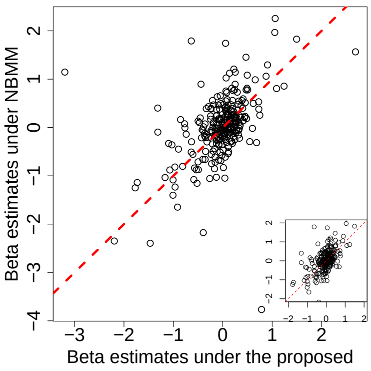

(q) Phosphate

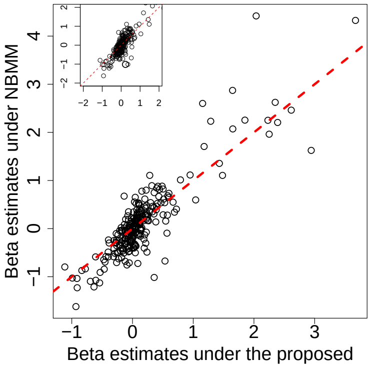

(r) X_8

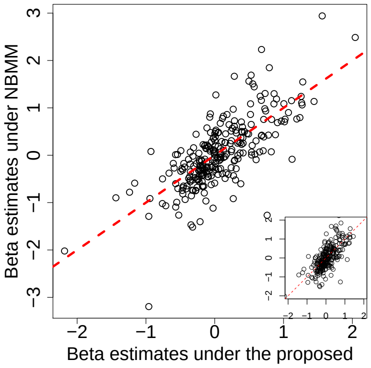

(s) X_9

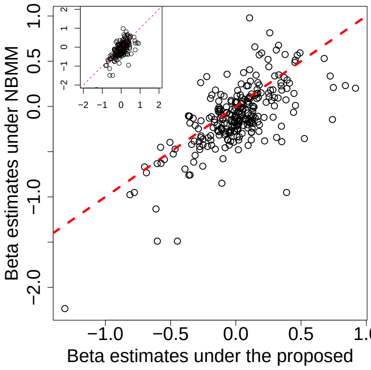

(t) X_{10}

Figure 15 (continued) [Ocean Microbiome Dat - Proposed Model vs NBMM] Comparison of the posterior mean estimates $\hat{\beta}_{j,p}$ under the proposed model and maximum likelihood estimates $\hat{\beta}_{j,p}^{\text{NBMM}}$ under the negative binomial mixed model (NBMM) for each covariate (p). The insert plot in each panel is a scatter plot for regions with small values of estimates.
